# Large-Scale Phylogenomic Analysis Reveals the Complex Evolutionary History of Rabies Virus in Multiple Carnivore Hosts

**DOI:** 10.1371/journal.ppat.1006041

**Published:** 2016-12-15

**Authors:** Cécile Troupin, Laurent Dacheux, Marion Tanguy, Claude Sabeta, Hervé Blanc, Christiane Bouchier, Marco Vignuzzi, Sebastián Duchene, Edward C. Holmes, Hervé Bourhy

**Affiliations:** 1 Institut Pasteur, Unit Lyssavirus Dynamics and Host Adaptation, WHO Collaborating Centre for Reference and Research on Rabies, Paris, France; 2 Institut Pasteur, Genomics Platform, Paris, France; 3 Agricultural Research Council, Onderstepoort Veterinary Institute, OIE Rabies Reference Laboratory, Pretoria, South Africa; 4 Institut Pasteur, Centre National de la Recherche Scientifique UMR 3569, Viral Populations and Pathogenesis Unit, Paris, France; 5 Marie Bashir Institute for Infectious Diseases and Biosecurity, Charles Perkins Centre, School of Life and Environmental Sciences and Sydney Medical School, The University of Sydney, Sydney, Australia; 6 Centre for Systems Genomics, University of Melbourne, Parkville, Victoria, Australia; Cornell University, UNITED STATES

## Abstract

The natural evolution of rabies virus (RABV) provides a potent example of multiple host shifts and an important opportunity to determine the mechanisms that underpin viral emergence. Using 321 genome sequences spanning an unprecedented diversity of RABV, we compared evolutionary rates and selection pressures in viruses sampled from multiple primary host shifts that occurred on various continents. Two major phylogenetic groups, bat-related RABV and dog-related RABV, experiencing markedly different evolutionary dynamics were identified. While no correlation between time and genetic divergence was found in bat-related RABV, the evolution of dog-related RABV followed a generally clock-like structure, although with a relatively low evolutionary rate. Subsequent molecular clock dating indicated that dog-related RABV likely underwent a rapid global spread following the intensification of intercontinental trade starting in the 15^th^ century. Strikingly, although dog RABV has jumped to various wildlife species from the order *Carnivora*, we found no clear evidence that these host-jumping events involved adaptive evolution, with RABV instead characterized by strong purifying selection, suggesting that ecological processes also play an important role in shaping patterns of emergence. However, specific amino acid changes were associated with the parallel emergence of RABV in ferret-badgers in Asia, and some host shifts were associated with increases in evolutionary rate, particularly in the ferret-badger and mongoose, implying that changes in host species can have important impacts on evolutionary dynamics.

## Author Summary

Zoonoses account for most recently emerged infectious diseases of humans, although little is known about the evolutionary mechanisms involved in cross-species virus transmission. Understanding the evolutionary patterns and processes that underpin such cross-species transmission is of importance for predicting the spread of zoonotic infections, and hence to their ultimate control. We present a large-scale and detailed reconstruction of the evolutionary history of rabies virus (RABV) in domestic and wildlife animal species. RABV is of particular interest as it is capable of infecting many mammals but, paradoxically, is only maintained in distinct epidemiological cycles associated with animal species from the orders *Carnivora* and *Chiroptera*. We show that bat-related RABV and dog-related RABV have experienced very different evolutionary dynamics, and that host jumps are sometimes characterized by significant increases in evolutionary rate. Among *Carnivora*, the association between RABV and particular host species most likely arose from a combination of the historical human-mediated spread of the virus and jumps into new primary host species. In addition, we show that changes in host species are associated with multiple evolutionary pathways including the occurrence of host-specific parallel evolution. Overall, our data indicate that the establishment of dog-related RABV in new carnivore hosts may only require subtle adaptive evolution.

## Introduction

Revealing how viruses jump species boundaries and establish productive infections in new hosts is key to understanding disease emergence. As most recent emerging and re-emerging viruses have RNA genomes [1], it is of central importance to understand the drivers of RNA virus evolution, diversification and cross-species transmission. Clearly, successful virus emergence has diverse causes, likely involving anthropogenic, social and environmental factors [2]. However, the capacity of the viral genome to vary and generate advantageous mutations is also an important element, enabling RNA viruses to exploit new niches, including novel host species, often more rapidly than DNA-based organisms [1, 3, 4]. One important manifestation of RNA virus evolution and diversification is the rate of evolutionary change (i.e. nucleotide substitution), with analyses of how this parameter varies by host species providing important information on the nature of virus-host interactions.

Disease emergence results from complex mechanisms that shape the ability of a virus to be maintained within its primary host species, then be serially transmitted to a new host species and initiate a pathologic process to cause disease [5]. As such, lyssaviruses (family *Rhabdoviridae*), the causative agents of rabies–an acute and almost invariably fatal encephalomyelitis in humans–represent an informative case study to examine the relationship between virus genetic diversity and disease emergence. In particular, the natural history of these zoonotic viruses provides an excellent model to study how replication in different host species alters the selection pressures that act on virus genomes. Lyssaviruses are single-stranded, negative-sense RNA viruses with a genome size of approximately 12 kb that encodes five proteins: the nucleoprotein (N), the phosphoprotein (P), the matrix protein (M), the glycoprotein (G) and the Large protein or polymerase (L). Currently, the lyssaviruses are classified into 14 species and one tentative species [6]. Like other RNA viruses, lyssaviruses exhibit high rates of mutation due to a lack of proofreading activity in the L protein [7]. Notably, although many mammalian species appear to be susceptible to lyssavirus infection, the virus is only able to establish sustained transmission networks in a relatively small number, indicating that there are major barriers to successful cross-species transmission [8–11].

One species of lyssavirus, rabies virus (RABV), is present worldwide and circulates in a diverse set of reservoir hosts among the mammalian orders *Chiroptera* and *Carnivora* [12]. Its natural evolution provides an illustrative example of multiple host switches, in turn enabling comparative studies of the evolutionary patterns, processes and dynamics associated with host adaptation. Previous studies demonstrated that RABV isolates fall into two major phylogenetic groups; the bat- and the dog-related RABV groups [8, 13, 14]. The ‘bat-related’ RABV group is confined to New World viruses circulating mainly among bats, as well as in some terrestrial carnivores such as skunks and raccoons [14–17]. In contrast, the ‘dog-related’ RABV group contains viruses circulating worldwide in dogs, as well as in wildlife carnivores in specific geographic areas such as foxes and raccoon dogs in Europe, foxes in the Middle East, raccoon dogs and ferret-badgers in Asia, skunks, foxes, coyotes and mongooses in the Americas, and mongooses in Africa [14, 16, 18–22]. Importantly, dogs are responsible for more than 99% of the human rabies cases worldwide [23] and are likely the main vector for the inter-species transmission of dog-related RABV.

Previous phylogenetic analyses have largely been performed on individual genes [13–19, 21, 24–29] with a few assessing the full-length viral genome [20, 30, 31]. In addition, most of these phylogenetic studies were performed on relatively small numbers of sequences originating from one specific geographical area and/or associated with a specific animal host [20, 22, 30, 32, 33]. Despite these limitations, these studies are consistent in showing that RABV is subject to strong purifying selection [10] coupled to geographical clustering that is occasionally disrupted by human mediated dispersion [13, 34, 35]. Recently, it was shown that nucleotide substitution rates in RABV vary markedly among those viruses infecting bats, such that rates in tropical and subtropical species were markedly higher than those from temporal bat species, perhaps reflecting a combination of host and environmental factors [36]. However, equivalent data for dog-related RABV are lacking. In addition, whether evolutionary rates in RABV vary among wild carnivores and domestic dogs is unknown, although studies in other systems have revealed that rates of RNA virus evolution may differ between wild and domestic animals [37]. Clearly, the large-scale analysis of RABV, particularly comprising full-length genome sequences, is needed to reveal the nature of the selection pressures associated with host switching. That the RABV genome encodes a limited number of proteins that necessarily have multifunctional roles [38], and hence potentially large-scale epistasis, also means that these selection pressures may be complex.

Herein we present the first phylogenetic study of RABV on a genome-wide and global scale, utilizing a data set of 321 whole-genome sequences sampled from 66 countries over a time period of 65 years, with the aim of inferring those evolutionary patterns and processes associated with host-switching. In particular, we compared RABV from wild carnivores and in domestic dogs with respect to selection pressures, evolutionary rates, and the time-scale of their evolutionary history. Importantly, the size of the data set allowed us to reveal any heterogeneity in evolutionary rates among RABV adapted to different primary hosts, and determine the complex evolutionary dynamics of RABV as it adapts to new hosts.

## Results

### Host and geographical clustering of RABV

**Fig 1:**
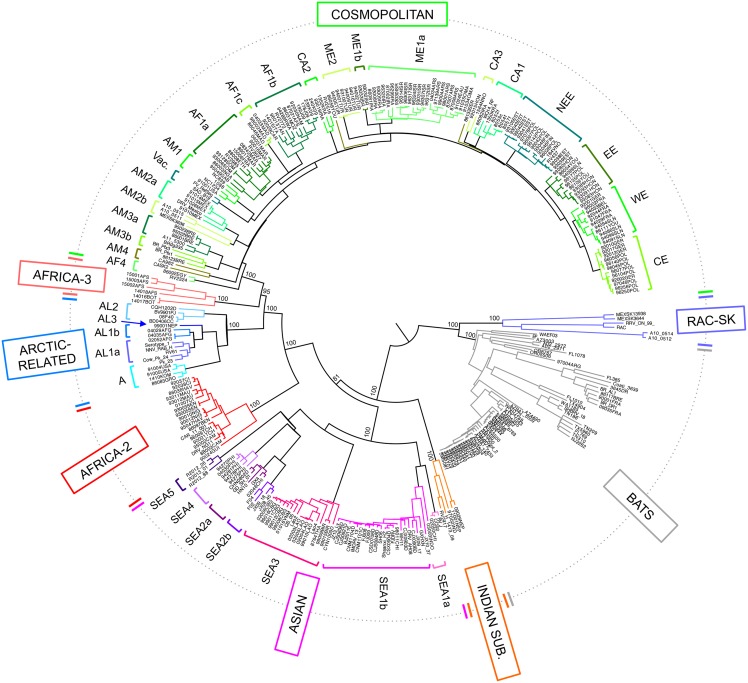
Maximum likelihood phylogeny of 321 RABV sequences from five concatenated genes. The major clades of RABV are indicated in boxes. The names of subclades and lineages defined for the Arctic-related, Asian and Cosmopolitan clades are detailed in S1 Table, with corresponding bootstrap values shown for major nodes. The tree is mid-point rooted for clarity only, and shows the division into bat-related RABV including the RAC-SK and bat clades, and dog-related RABV including the Africa-2, Africa-3, Arctic-related, Asian, Indian subcontinent and Cosmopolitan clades.

A phylogenetic analysis was performed on the (99%) full-length genome sequences of 321 RABV sequences sampled from 66 countries (S1 Fig, S1 Table). Of these viruses, 170 were newly sequenced as part of this study. As expected given the low levels of recombination in RABV, the topology of the maximum likelihood (ML) tree performed on the five concatenated RABV genes (Fig 1) was similar to that obtained for each individual gene (N, P, M, G and L genes) and for the concatenated non-coding sequence (S2 Fig). In particular, two major phylogenetic groups were apparent, corresponding to bat- and dog-related RABVs, each of which can be further subdivided into several major clades. This is consistent with previous analyses of smaller data sets and on individual RABV genes [13, 14, 16, 29].

The bat-related group contained two major clades, one including the bat RABVs circulating in the Americas, and the other (RAC-SK) comprising viruses from American skunks and raccoons (Fig 1). In turn, the RAC-SK group contained a number of ‘subclades’ corresponding to Mexican skunks (MeSK-1), North American raccoons (RAC) and South-Central skunks (SCSK) as previously described (S3 Fig) [14, 16, 17, 39].

Similarly, the dog-related group includes six major clades supported by high bootstrap values (S2 Table and Fig 1), and previously identified as the Africa-2, Africa-3, Arctic-related, Asian, Cosmopolitan and Indian subcontinent clades [13]. The phylogenetic analysis based on the five concatenated genes was particularly informative, allowing us to distinguish various subclades and lineages among these six major clades, with some of which are characterized for the first time here (Fig 1 and supplementary text).

Some of these clades and subclades are of particular interest. The SEA2 subclade contains viruses from China and is divided into two lineages, SEA2a and SEA2b, corresponding to isolates from dogs and ferret-badgers, respectively. Subclade SEA5 appears to be specific to RABV circulating in ferret-badgers in Taiwan, an epidemiological cycle that was only identified recently [21, 40, 41]. For the first time, we were also able to fully characterize full-length genome sequences of RABV isolates belonging to the Africa-3 clade (n = 6). These viruses circulating in Southern Africa are monophyletic and phylogenetically distinct from the other major RABV clades, particularly those circulating in Africa [19, 42, 43].

### Temporal dynamics and spread of the dog-related RABV

To determine the evolutionary dynamics of RABV, we first determined whether individual data sets contained sufficient temporal structure to undertake detailed molecular clock analyses by performing a regression of root-to-tip genetic distance against the year of sampling. Notably, no correlation between time and genetic divergence was found when the sequences of both bat- and dog-related RABV groups were analyzed together, indicating that there is extensive variation in the rate of RABV evolution among these taxa (and hence that they should not be combined in molecular clock studies) (S4A Fig). In addition, no temporal structure was observed when the sequences of bat-related RABV were analyzed separately, indicating that this subset of viruses is not evolving uniformly (S4B Fig) as noted previously [36]. However, a clear association between genetic distance and time (i.e. a molecular clock) was observed for the dog-related group alone (S4C Fig), allowing us to estimate substitution rates, and hence times to common ancestry, more precisely in this cluster using a Bayesian approach.

**Fig 2:**
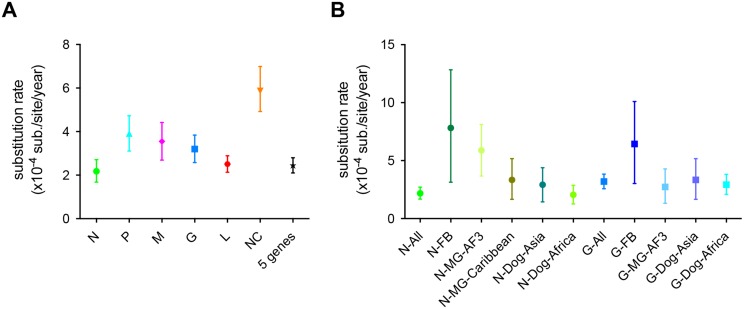
Evolutionary rates of RABV genes in the dog-related group. (**A**) Rates of nucleotide substitution per site, per year were estimated for each RABV gene: nucleoprotein (N), phosphoprotein (P), matrix (M), glycoprotein (G) and polymerase (L), for the concatenated non-coding regions (NC) and for the five concatenated RABV genes (5 genes). Both the mean and the 95% highest posterior density (HPD) values on the rate are shown. (**B**) Substitution rates in the N and G genes of the dog-related group RABV, a sub-set of RABV circulating in mongooses (MG) in Africa-3 clade and in the Caribbean, in ferret-badgers (FB) in Asia, and in dogs in Asia and Africa. Note the different y-axes (rates) in both cases.

The mean rate of evolutionary change in the dog-related RABV was estimated to be 2.44 x 10^−4^ subs/site/year (95% HPDs of 2.10–2.80 x 10^−4^ subs/site/year) for the five concatenated genes. Importantly, we were also able to compare the substitution rate of each RABV gene and of the concatenated non-coding regions from the same genomic sequence data set. These estimates varied in the following ascending order: N, L, G, M and P (Fig 2A). However, only the P gene had a nucleotide substitution rate considerably higher than those of N and L genes. As expected, the evolutionary rate in the non-coding regions was significantly higher than those of the coding regions, indicative of weaker selective constraints.

**Fig 3:**
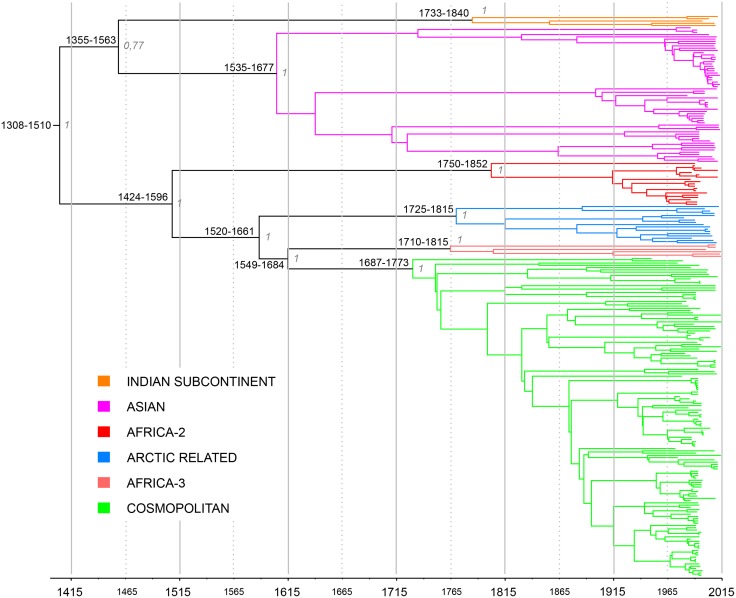
Maximum clade credibility phylogeny of 248 dog-related RABV utilizing five concatenated genes. Tip times represent the time (year) of sampling. Bayesian estimates of divergence time are also shown. Upper and lower limits of the 95% highest posterior density (HPD) estimates and the posterior probability values are shown for major nodes.

The estimation of a reliable substitution rate allowed us to determine the mean times to common ancestry (TMRCA) for each RABV clade, subclade and lineage defined above (Fig 3; estimates for different sub-clades shown in S3 Table and discussed in the Supplementary text). For this analysis we utilized the concatenated coding genes as these had the lowest variance. Notably, these TMRCA estimates exhibited less uncertainty than previous studies performed on N and/or G genes alone [13, 25, 27, 29, 43, 44]. Briefly, the TMRCA of the dog-related RABV group was estimated to be approximately between 1308–1510 (95% HPD; mean of 1404). Within this group, the Indian subcontinent clade branched basally and appeared to diversify between 1733–1840 (mean of 1785). The TMRCA of the Asian clade was estimated to be between 1535–1677 (mean of 1604), which is in accordance with other studies [44]. The emergence of the Africa 2 clade was estimated to be between 1750–1852 (mean of 1802), similar to the mean TMRCA found in a previous study conducted on complete N and G genes [27]. The Arctic-related clade appeared between 1725–1815 (mean of 1770), slightly earlier than previously estimated [25], while the Africa-3 clade emerged between 1710–1815 (mean of 1756) in accordance with another study [43]. Finally, for the first time, we estimate that the TMRCA for the Cosmopolitan clade existed between 1687–1773 (mean of 1730).

### Variation in evolutionary rates among hosts and selection pressures along the RABV genome

The root-to-tip regression analysis also revealed that different groups of dog-related RABV have seemingly evolved at different rates (S4C Fig), with a number appearing as distinct outliers. Interestingly, these outliers were confined to RABV circulating in mongooses in Africa (Africa-3 clade) and in ferret-badgers in Asia (the SEA5 subclade and SEA2b lineage), suggesting that they might represent species-specific variation. To address this, we compared the evolutionary rates of these clusters to both the entire dog-related RABV group and to subsets of this group representing dog-related viruses circulating in Africa and Asia, and to mongoose viruses circulating in the Caribbean. For this analysis we focused on the N and G genes as they comprise the largest data sets. This analysis revealed that the N gene of those viruses circulating in ferret-badgers in Asia (n = 81) and in mongooses in Africa (n = 47) evolved between 2–4 times more rapidly than those of the whole dog-related group (n = 248), at rates of 7.82 x 10^−4^ subs/site/year (95% HPD 3.14–12.83 x 10^−4^ subs/site/year) and 5.88 x 10^−4^ subs/site/year (95% HPD 3.67–8.11 x 10^−4^ subs/site/year), respectively (Fig 2B). Importantly, these estimates and their associated uncertainty do not overlap with those for the dog-related group as a whole. This finding is confirmed using smaller subsets of dog-related RABV from more closely related geographically settings in Asia and in Africa (Fig 2B). Interestingly, the rate of RABV evolution in mongooses in Africa is two times higher than that of RABVs from mongooses in the Caribbean (i.e. Puerto Rico, Cuba and Grenada) that belong to the Cosmopolitan clade (Fig 2B). Although less rate variation was observed in the G gene, RABV associated with ferret-badgers in Asia still evolved considerably more rapidly than those obtained with the different subsets of dog-related RABV (Fig 2B). These results were confirmed by using different nucleotide substitution models and a hierarchical phylogenetic model approach (S4 Table) [45, 46].

To determine if the variation in rates of evolutionary change might result from differing selection pressures, we first compared the ratio of nonsynonymous (*d*_*N*_*)* to synonymous *(d*_*S*_*)* substitutions per site. This analysis was performed on each of the five RABV genes of the two major RABV groups. For each gene, the *d*_*N*_*/d*_*S*_ ratios of the bat- and dog-related groups are very similar (and very low) and followed the same ascending order between genes: N, L, M, G and P genes (Table 1). Furthermore, we explored the number of positively selected sites using several different approaches (SLAC, FUBAR and FEL) [47, 48]. In each of the two major RABV groups, one position was identified as positively selected by at least two of these methods: positions 496 and 484 in the G protein for the bat- and dog-related groups, respectively (Table 1). Interestingly, the *d*_*N*_*/d*_*S*_ of the N and G genes for the branches leading to sequences found to be outliers in the analysis of evolutionary rates (Africa-3 clade and ferret-badgers in Asia) were 1.4 to 4.7 times higher than those of dog-related RABV data sets used as controls (S4C Fig and S5 Table), but still relatively low. Together, these results are generally indicative of strong purifying selection among all sites and branches of the RABV phylogeny.

**Table 1:** Selection pressures in five genes from bat- and dog-related RABVs.

Data set	Gene	*d*_*N*_*/d*_*S*_	SLAC^a^	FEL^a^	MEME-internal^a^	FUBAR^b^
Bat–related RABV (n = 67)	N	0.045	-	-	ND	-
	P	0.139	-	-	ND	-
	M	0.063	-	-	ND	-
	G	0.133	496	175, 496	ND	-
	L	0.044	-	-	ND	-
Dog-related RABV (n = 254)	N	0.032	-	-	436	-
	P	0.126	-	-	55, 154, 265	-
	M	0.068	-	-	**-**	-
	G	0.099	484	484	198, 476	484
	L	0.038	-	-	430, 681, 2091	-

*d*_*N*_*/d*_*S*_ ratios are calculated using SLAC

Putatively positively selected codons identified by more than one method are underlined

^a^ Codons with p-value < 0.05

^b^ Codons with posterior of probability > 0.95

ND: not done

To investigate selection pressures in greater detail we utilized a modified MEME analysis that considered internal branches of the tree only (as external branches often contain transient deleterious nonsynonymous substitutions yet to be removed by purifying selection) [49]. Using this MEME-internal analysis, we identified nine positions to be under positive selection (N436, P55, P154, P265, G198, G476, L430, L681, L2091). In addition, position G484 that was identified as positively selected using SLAC, FUBAR and FEL was not significant (at the p<0.05 level) in the MEME-internal analysis (p-value = 0.084).

Finally, it was also clear that specific amino acid substitutions characterized RABV circulating in mongooses in Africa (Africa-3 clade) and in ferret-badgers in Asia (SEA5 subclade and SEA2b lineage) (S6 Table). Four substitutions were specific (i.e. not present in any other dog-related RABV sequences) to mongoose RABV: two in the nucleoprotein, from Asp to Asn at codon position 88 (Asp-N88-Asn) and Leu-N108-Ile, and two in the glycoprotein–Ser-G223-Asn and Pro-G386-Ser. The case of the ferret-badger was more interesting as the host jump to this species from dogs has occurred independently in the SEA5 and SEA2b clades, allowing us to determine whether cross-species transmission in this case is associated with parallel viral evolution. This analysis revealed that two amino acid substitutions were common to all ferret-badger viruses across both clades: Leu-N374-Ser and Lys-L200-Arg. The Leu-N374-Ser substitution is particularly noteworthy as it only occurs in the ferret-badger, this residue is normally highly conserved in RABV, and Leu-to-Ser is a non-conservative amino acid change. Hence, we suspect that Leu-N374-Ser, and perhaps Lys-L200-Arg, facilitate RABV adaptation to ferret-badgers. Notably, neither of these sites was found to be subject to positive selection using the methods employed here (Table 1).

## Discussion

The central aim of this study was to determine whether the patterns and processes of RABV evolution vary between viruses sampled from different host species reflect the impact of cross-species transmission. To that end we present the largest phylogenomic analysis of RABVs circulating worldwide performed to date. Although the topology of the RABV phylogeny is similar to those obtained previously [13, 14, 16, 29], it clearly presents a more comprehensive and precise reconstruction of evolutionary history of this virus. In particular, the analysis of the five concatenated genes allowed us to obtain a finer-scale dating of the emergence of the major clades with narrower confidence intervals than obtained previously [13, 25, 27, 29, 43, 44].

RABV undergoes relatively frequent cross-species transmission [8, 11, 13, 18], which provides an opportunity to determine whether host jumping impacts rates of evolutionary change. Notably, we found no correlation between root-to-tip genetic distance and sampling time in the bat-related RABV group, nor when combined with dog-related RABV group, indicating that these viruses have not evolved in a clock-like manner, with substantial rate variation already observed in bat-associated RABV [36]. In contrast, a strong association between genetic divergence and time (i.e. a molecular clock) was observed within the dog-related RABV group, with a mean evolutionary rate of 2.44 x 10^−4^ subs/site/year (95% HPDs of 2.10–2.80 x 10^−4^ subs/site/year) for the five concatenated genes. This estimate is evidently more precise than those determined previously [13, 25, 27, 30, 44, 50–53].

Despite the relative rate constancy in the dog-related RABV, it was striking that some of the clades or sub-clades have experienced substantially higher rates of nucleotide substitution. In particular, viruses circulating in ferret-badgers in Asia (mainland China and Taiwan) and in mongooses in Africa have evolved at least twice as rapidly as those of the dog-related group. Although there is some uncertainty in these rate estimates, they do not overlap with the estimates for the entire dog-related RABV group. Determining the evolutionary basis to this rate variation is more complex. Changes in evolutionary rate could only be driven either by changes in background mutation rate (which we consider unlikely to differ between dog-related RABV) or, more likely, by changes in the population size and/or incubation time that may vary among different animal hosts [36]. It is also possible that the evolutionary rates estimated here have been impacted by time-dependency, such that they are elevated toward the present (*i*.*e*. in closely related sequences sampled recently) due to the presence of transient deleterious mutations that have yet to be removed by purifying selection [54]. However, while this may in part explain the high rate in the recently sampled RABV from ferret-badgers, it is unlikely to explain the higher evolutionary rate in mongoose RABV whose evolutionary history sampled here covers a longer time period. In the case of the ferret-badgers, two amino acid changes (Leu-N374-Ser and Lys-L200-Arg) have evolved in parallel in the two clades associated which is compatible with the occurrence of adaptive evolution, and which have in turn elevated the nucleotide substitution rate. That these two sites were not detected in analyses of *d*_*N*_*/d*_*S*_ suggests that these methods may have limitations when identifying adaptive evolution involving limited amounts of amino acid change.

Our analysis also showed that the nucleotide substitution rate varied markedly according to the gene analyzed in the ascending order: N, L, G, M and P. As expected, the two proteins often described as more conserved for RABV—N and L—exhibited the lowest rates, as well as the lowest *d*_*N*_*/d*_*S*_ ratios, indicating that they are subject to the strongest purifying selection. Notably, the highest substitution rate and *d*_*N*_*/d*_*S*_ was observed in the P protein, perhaps reflecting the weak structural organization of the C-term part of this protein [55, 56].

The presence of relatively constant molecular clock also enabled us to provide a more robust time-scale for the evolution of the principal geographical clusters of dog-related RABV (Fig 3, S3 Table). Accordingly, we estimate that the most recent ancestor of all dog-related RABV dates to between 1308 and 1510. Consequently, any older canid RABV lineages, proposed to have circulated in the Middle-East more than 2000 years ago [57, 58], have not survived to be sampled in the current study. Interestingly, the timing of the most recent ancestor of all dog-related RABV circulating to date coincides with the development of the world's first truly global trade network following the explorations of Columbus, Vasco da Gama and Zheng He, commissioned by the Spanish, the Portuguese and the Chinese Ming Dynasty, respectively. This age of exploration and colonization contributed to the establishment of new long distance commercial practices and transoceanic shipping services between 1450 and 1750 [59]. The concomitant dissemination of RABV during this period, probably by dogs travelling by boats with their owners, therefore provides a powerful example of the early human-mediated dissemination of a zoonotic disease. In addition, all the ancestors of the major clades found circulating today in North and South America, Africa, Asia and Europe originated between 1687 and 1840 at the apogee of this international trade and colonization process [59]. This is further exemplified by the global spread of the Cosmopolitan clade.

A fundamental question in evolutionary virology is how and why some viruses are seemingly better able to jump species boundaries than others. A compelling theory is that the more closely related the host species in questions, the greater the chance of successful transmission [9, 60, 61]. However, it is unclear how strictly this theory holds for RABV [11], and our results confirm species jumps of RABV among animal species of the order *Chiroptera*, and from bats to striped skunks (*Mephitis mephitis*) [14, 62, 63]. In addition, there is also clearly a geographic component to cross-species transmission as bat-related RABVs are only found in the Americas. More notably, our study clearly confirms that although spill-over infections from wildlife species to dog take place, species jumps involving dog-related RABVs generally occur *from* dogs to wildlife species of the order *Carnivora;* not only to the family *Canidae* (dog, red fox, raccoon dog), but also to more distant species belonging to the families *Mustelidae* (ferret-badger), *Herpestidae* (mongoose) and *Mephetidae* (skunk) (S5 Fig) [13, 18, 42, 43]. These changes in primary animal host species have occurred independently in different localities and at different times during RABV evolution. Further, some carnivore species, notably skunks, are infected by RABV of both dog and bat origin [14, 16, 39].

Revealing the respective roles of genetic drift and the selection of advantageous mutations in shaping the genetic diversity of RABV, particularly during host shifts, is a central evolutionary question. There is currently no definitive data on whether dog-related RABV emergence requires active adaptive evolution (i.e. positive selection) to in a new host species, or whether it is largely a chance process involving ecological factors facilitating the transmission of a viral strain with the pre-existing necessary genetic characteristics [64]; the latter has been proposed for the repeated outbreaks of bat-related RABV in striped skunks and gray foxes in Arizona [14] and of gray foxes due to skunk-associated RABV in California [65]. Our analysis showed that the dog-related RABV group is subject to strong purifying selection, and when positive selection did occur on internal branches of the phylogenetic tree it was not obviously associated with host jumping. As noted above, however, the failure to detect positive selection in the case of ferret-badger RABV despite the occurrence of parallel evolution suggests that these methods may suffer from false-negatives.

Successful cross-species transmission is a complex ecological and evolutionary process, beginning with exposure and contact between the two species, followed by the successful infection of the new host species, and potentially host-adaptive evolution to enable long-term sustained transmission [66, 67]. However, due to complex interactions among the five viral proteins and with their cellular counterparts, including epistasis [68], it is often difficult to clearly determine which mutations are advantageous or fixed by genetic drift. Moreover, some mutations in the RABV P protein can improve the modulation of the innate immune response of the host but reduce replication efficiency [69]. That two amino acid changes have evolved in parallel in the ferret-badger alone suggests that they have played a role in host adaptation. Further, it is possible that some of the other amino acid substitutions that define individual viral clades associated with different host species represent host-adaptive sites that have not been identified as positively selected through simple analyses of *d*_*N*_*/d*_*S*_. Clearly, additional large-scale analyses of RABV based on full-length genome sequences, extending that presented here, followed by linked experimental studies including generation of mutant RABVs by reverse genetics and phenotypic testing, are needed to reveal the nature of complex evolutionary processes that occur during host switching.

In conclusion, RABV is capable of infecting many mammals but paradoxically is maintained in distinct epidemiological cycles associated with animals almost exclusively from the orders *Carnivora* and *Chiroptera*. This strict association between RABV and host-species most likely arose from a combination of historical human-mediated spread of RABV and jumps into new primary host species. These data also suggest that the establishment of dog-related RABV in new carnivore hosts may only require subtle adaptive evolution as demonstrated by parallel evolution in the ferret-badger. Evidently, along with more defined analyses of individual mutations, additional studies are needed to determine the role played by the frequency of exposure, animal host behavior, density of the recipient species, duration of incubation and optimum infectious doses in cross-species transmission.

## Materials & Methods

### Samples

A total of 321 complete genome sequences of RABV isolates were analysed, originating from a wide variety of host species and collected in 66 countries between 1950 and 2015. Details of these isolates are described in S1 Table and S1 Fig. Among these genome sequences, 170 came from the archives of the World Health Organization Collaborative Center for Reference and Research on Rabies, or from the National Reference Centre for Rabies, both located at Institut Pasteur, Paris, France. These samples were newly sequenced as part of this study. These data were combined with 151 full-length genome sequences extracted from GenBank and selected to be representative of the overall phylogenetic diversity of RABV.

### RNA extraction and next-generation sequencing

Total RNA was extracted using Trizol (Ambion) according to the manufacturer’s instructions from primary brain samples or after an amplification passage on suckling mouse brain. RNA was then reverse transcribed using Superscript III reverse transcriptase with random hexamers (Invitrogen) according to manufacturer’s instructions. The complete viral genome (excluding the 3’ and 5’ extremities, corresponding to the leader and the trailer regions, respectively) of 160 isolates was amplified with six overlapping PCR fragments by using the Phusion polymerase (ThermoFisher). Details of primers are given in S7 Table. After electrophoresis, each PCR fragment was independently purified using the NucleoSpin Gel and PCR clean-up kit (Macherey-Nagel) and quantified using Picogreen dsDNA quantification kit (Invitrogen). For each sample, all six PCR fragments were pooled with equimolar proportions to obtain 500 ng of dsDNA.

Different protocols were used for the preparation of libraries and next-generation sequencing on Illumina platforms (NextSeq 500, HiSeq2000, HiSeq2500 or MiSeq platforms), depending on the isolates considered (details provided in S1 Table). Briefly, three different protocols were used: (i) dsDNA was fragmented by ultrasound with Bioruptor (Diagenode), libraries were prepared using NEXTflex PCR-Free DNA-Seq kit (Bioo Scientific), and then sequenced using an 100 or 150 nucleotides single-end strategy on the HiSeq2500 platform or a 2 x 300 nucleotides paired-end strategy on the MiSeq platform, (ii) dsDNA was fragmented by NEBNext dsDNA fragmentase (New England Biolabs), libraries were prepared using NEBNext Ultra DNA Library Prep kit (New England Biolabs) and sequenced using an 100 nucleotides single-end strategy on the NextSeq500 platform, and (iii) dsDNA libraries were constructed using Nextera XT kit (Illumina) and sequenced using a 2 x 150 nucleotides paired-end strategy on the NextSeq500 platform. For nine remaining isolates (S1 Table), the viral RNAs were reverse transcribed using Superscript III reverse transcriptase (Invitrogen) and then amplified using the whole-transcription amplification (WTA) protocol (QuantiTect Whole Transcriptome kit; Qiagen) as previously described [70]. dsDNA was fragmented by ultrasound, libraries were prepared using TruSeq protocol (Illumina) and sequenced using an 100 nucleotides single-end strategy on the HiSeq2000 platform. Finally, the sequence of 09035FRA was determined using a shotgun base approach [31].

### Genome sequence analyses

All reads were pre-processed to remove low-quality or artifactual bases. Library adapters, PCR primers used for amplification of the genome, and base pairs occurring at 5’ and 3’ ends with a Phred quality score <25 were trimmed using AlienTrimmer as implemented in Galaxy [71–74] (https://research.pasteur.fr/en/tool/pasteur-galaxy-platform/). Reads with lengths of less than half of the original read after these pre-processing steps or those containing >20% of bp with a Phred score of <25 were discarded. The filtered reads were then mapped to complete genome sequences specific for each RABV clade obtained from GenBank using the CLC Genomics Assembly Cell (http://www.clcbio.com/products/clc-assembly-cell/) implemented in Galaxy. The majority nucleotide (>50%) at each position with a minimum of coverage of 200 was used to generate the consensus sequence.

All consensus sequences were manually inspected for accuracy, such as the presence of intact open reading frames, using BioEdit (http://www.mbio.ncsu.edu/bioedit/bioedit.html). A sequence alignment of the 170 newly sequenced genomes combined with the 151 complete genome sequences from GenBank was constructed using ClustalW2 with default parameters [75] (http://www.ebi.ac.uk/Tools/msa/clustalw2/) implemented in Galaxy and manually adjusted when necessary. Sequence alignments of individual RABV genes (N, P, M, G and L genes) and concatenated non-coding regions (from the stop codon in N to the initiation codon of L) were also generated. All the full-length genome sequences generated in the present study have been submitted to GenBank (S1 Table).

### Phylogenetic analysis

We used jModelTest2 [76, 77] to determine the best-fit model of nucleotide substitution according to the Bayesian Information Criterion. This revealed that the general time reversible model with proportion of invariable sites plus gamma-distributed rate heterogeneity (GTR+I+Γ_4_) was optimal for all the RABV data sets compiled here. Phylogenetic trees using the different data sets (i.e. individual genes, concatenated genes or non-coding regions) were then estimated using the maximum likelihood (ML) method available in PhyML 3.0 [78] utilizing SPR branch-swapping. The robustness of individual nodes on the phylogeny was estimated using 1,000 bootstrap replicates for the five concatenated gene data set, and using the approximate likelihood ratio test (aLRT) with SH-like supports for each individual RABV gene as well as the concatenated non-coding region data set [79].

### Estimates of RABV evolutionary dynamics and time-scale

To determine the degree of clock-like structure in each data set we employed root-to-tip linear regression as available in the TempEst program [80]. For those data sets with sufficient phylogenetic structure we then inferred a maximum clade credibility (MCC) tree using the Bayesian Markov chain Monte Carlo (MCMC) method available in the BEAST v1.8 package [81] by incorporating information on sampling time (year) of the dog-related RABV group (isolates for which the date of sampling was unavailable and vaccine strains were excluded). Posterior probability values provided an assessment of the degree of support for each node on the tree. This analysis utilized the GTR+I+Γ_4_ model of nucleotide substitution, a relaxed (uncorrelated log-normal) molecular clock and the constant population size model as a coalescent prior. Ten independent MCMC analyses were run for 100 million steps and sampled every 10,000 states. The log and tree files of each MCMC chains were combined using Logcombiner v1.8.2 (http://tree.bio.ed.ac.uk/software/beast/), with a burn-in of 10%. The convergence of each parameter in this combined file was checked using TRACER v1.6 (http://tree.bio.ed.ac.uk/software/tracer/) and indicated by an effective sample size >200. The MCC tree was obtained using TreeAnnotator v1.8.2 (http://tree.bio.ed.ac.uk/software/beast/). Additional analyses were performed utilizing the GMRF Bayesian Skyride [82] and Bayesian SkyGrid [83] demographic models, and gave similar results.

Based on the BEAST analysis, we also estimated the rate of nucleotide substitution per site, per year (see below) and the time of most recent common ancestor (TRMCA) for host-specific clusters of sequences. The degree of statistical uncertainty in each parameter estimate was given by the 95% highest posterior density (HPD) values.

The root-to-tip regression analysis performed on the 248 sequences of the dog-related RABV group revealed a number of clear outlier taxa characterized by anomalously high evolutionary rates (S4C Fig). These outliers belong to three clades or sub-clades: the Africa-3 clade that is specific to mongooses in Southern Africa, and the SEA5 and SEA2b subclades that are confined to viruses from ferret-badgers in Taiwan and China, respectively. To further assess if there are considerable differences in evolutionary rate in these clades, we performed additional analyses on the N and the G proteins for which relatively large numbers of sequences were available on GenBank. We therefore collected from GenBank an additional 41 N and 26 G sequences from the Africa-3 clade, and an additional 72 N and 71 G sequences from the ferret-badger in Taiwan and China (S8 Table). These data sets were compared to the N and G sequences of the dog-related RABV group (n = 248) and two RABV subsets corresponding to viruses circulating in dogs in Asia (n = 51) and in Africa (n = 46). As the Africa-3 clade is specific to the mongoose, we also estimated the evolutionary rate of RABV circulating in mongooses in the Caribbean region, for which we constructed a data set of 64 N sequences (no G sequences were available). As the ferret-badger data set was small, covered a relatively short time-range, and comprised two groups sampled during different time periods, it was unfortunately impossible to analyse the evolutionary dynamics in these two groups separately.

Estimates of nucleotide substitution rate of each data set were performed using BEAST as described above. Preliminary analysis on the N and G gene data sets using different nucleotide substitutions models (GTR+I+Γ_4_ or GTR+I), strict or relaxed (uncorrelated log-normal) molecular clocks, constant population size or Bayesian skyline coalescent priors gave similar results. Therefore, all analyses were performed using the GTR+I+Γ_4_ substitution model, a relaxed (uncorrelated log-normal) molecular clock, and a constant population size.

Finally, to assess the robustness of our rate estimates we also utilized and hierarchical phylogenetic models [46]. This analysis considered the lineages of the N and G genes defined previously (i.e. those viruses circulating in ferret-badgers, in mongooses in Southern Africa and the Caribbean, and in dogs in Africa and Asia) which we treated as data partitions. To be as robust as possible we used two substitution models–SRD06 [45] and GTR+I+Γ_4_. For the SRD06 model we specified hyperprior distributions to govern κ (the relative rate of transitions to transversions) and the shape parameter, α, of the Γ-distribution among the first and second, and third codon positions. In the case of the GTR+I+Γ_4_ model we linked each of the six rate parameters of the substitution matrix, α, and the proportion of invariable sites (I). Importantly, for both substitution models we set separate uncorrelated lognormal relaxed clock models and constant-size coalescent tree priors for each of the partitions, which is appropriate because they involve different taxa.

### Analysis of selection pressures

To reveal the selection pressures acting on the RABV genome we compared the numbers of nonsynonymous (*d*_*N*_) and synonymous (*d*_*S*_) substitutions per site for the different RABV genes and phylogenetic clusters using the Single Likelihood Ancestor Counting (SLAC), Fixed Effect Likelihood (FEL), the internal branch Mixed Effects of Model Evolution (MEME-internal; *Kosakovsky Pond SL*, *personal communication*) and the Fast Unbiased Bayesian Approximation (FUBAR) models [47–49]. Only codon positions with a p-value < 0.05 for the SLAC, FEL and MEME models and with a posterior of probability > 0.95 for the FUBAR method were considered as containing evidence for positive selection. For each data set and gene, the best-fit model of nucleotide substitution model was determined using the model selection tool available on the DATAMONKEY server [84, 85].

## Supporting Information

S1 Text Description of each dog-RABV clade, subclade and lineage identified in Fig 1 and their TRMCA estimates. (DOCX)

S1 Fig Geographic distribution of the 321 RABV isolates analyzed in the study. (**A**) Triangles and dots represent the bat- and dog-related RABV, respectively, with sizes proportional to the number of isolates as indicated in the legend. (**B**) The different colors represent the major clades in the bat- and dog-related RABV groups.
(PDF)

S2 Fig Comparison of the maximum likelihood phylogenies of 321 RABV sequences representing the N, P, M, G, L genes and concatenated non-coding regions. The ML trees are mid-point rooted and aLRT values are shown for each clade, subclade and lineage named according to Fig 1 and the nucleoprotein (A), phosphoprotein (B), matrix (C), glycoprotein (D), polymerase (E) genes and concatenated non-coding regions (F) are shown separately.
(PDF)

S3 Fig Maximum likelihood phylogeny of 67 bat-related RABV sequences representing five concatenated genes. The tree is mid-point rooted with bootstrap values shown for each major subclades. The subclade names are given according to Kuzmin *et al*., (2012) [14]. Subclade abbreviations: SCSK–South-Central skunk; RAC–North-American Raccoon; MexSK-1 –Mexican skunk, variant 1; EF-W1 and EF-W2 –*Eptesicus fuscus*, in western USA; MYu–*Myotis yumanensis*; LX–*Lasiurus xanthinus*; LS–*Lasiurus seminolus*; LC–*Lasiurus cinereus*; LB–*Lasiurus borealis*; PS–*Perimyotis subflavus*; LN–*Lasionycteris noctivagans*; LI–*Lasiurus intermedius*; TB–*Tadarida brasiliensis*; DR–*Desmodus rotundus*; MYsp–*Myotis* spp; PH–*Parastrellus hesperus*; AP–*Antrozous pallidus*; EF–*Eptesicus fuscus*, in eastern and central USA.
(PDF)

S4 Fig Root-to-tip regression of genetic distances against the year of sampling for five concatenated RABV genes. The root-to-tip regressions were obtained using TempEst [80], on (**A**) a combined bat- and dog-related RABV data set (n = 315), (**B**) bat-related RABV data set (n = 67), and (**C**) dog-related RABV data set (n = 248) (isolates for which the date of sampling were unavailable and vaccine strains were excluded). The red circle indicates a number of outlier strains characterized by anomalously high rates (employed an arbitrary cut-off). The inferred rate of nucleotide substitution corresponding to the slope and the correlation coefficient are also indicated.
(PDF)

S5 Fig Maximum likelihood tree of 321 RABV from the five concatenated genes labelled by host species. Tip names are colored according to the isolation species of each virus, dog in black, bat in grey, ferret-badger in magenta, mongoose in orange, human in red, other carnivores in green, herbivores and/or omnivores in blue and vaccine strains in purple. The major clades of RABV are indicated in boxes like in Fig 1. The names of subclades and lineages defined for the Arctic-related, Asian and Cosmopolitan clades are detailed in S1 Table. The tree is mid-point rooted for clarity only.
(PDF)

S1 Table List of viruses used in the full-length genome analyses. (DOCX)

S2 Table Bootstrap values of nodes corresponding to all clades, subclades and lineages of the dog-related group defined in Fig 1. (DOCX)

S3 Table Evolutionary characteristics of dog-related RABV group clades, subclades and lineages. (DOCX)

S4 Table Substitution rates of the N and G genes among different host species in the dog-related RABV group. (DOCX)

S5 Table Selection pressures in the N and G genes among different host species in the dog-related RABV group. (DOCX)

S6 Table Amino acid substitutions specific to mongoose-related RABV (Africa-3 clade) or ferret-badger-related RABV (SEA5 subclade and the SEA2b lineage). (DOCX)

S7 Table List of primers used in this study. (DOCX)

S8 Table List of additional nucleotide and glycoprotein sequences used to estimate the evolution rates among different hosts. (DOCX)
