# Supplementary material for: Large-Scale Phylogenomic Analysis Reveals the Complex Evolutionary History of Rabies Virus in Multiple Carnivore Hosts
Source: PLoS Pathog. 2016 Dec 15;12(12):e1006041. doi: 10.1371/journal.ppat.1006041 (PMC5158080; doi:10.1371/journal.ppat.1006041)
Supplement: S1 Text — (DOCX) [file ppat.1006041.s001.docx]

**Supplementary text**

**Results**

**Host and geographical clustering of RABV**

Of particular note from our analysis was the Indian-subcontinent clade (n=5) that occupied a basal position in the phylogenetic tree among the dog-related RABV group [1]. This clade is clearly a geographical variant and contains lineages from Nepal, India and Sri Lanka [2].

The RABV from the Asian clade (n=61) fell into five phylogenetic subclades, denoted Southeast Asia (SEA) 1 to 5, of which SEA1, 2 and 3 have been previously described [3]. The SEA1 subclade included isolates from China and Indonesia divided into two phylogenetic lineages, named SEA1a and SEA1b, respectively. The SEA2 subclade contained viruses from China is also divided into two lineages, SEA2a and SEA2b, corresponding to isolates from dogs and ferret-badgers, respectively. The SEA3 subclade included viruses from Cambodia, the South-Western China, Lao, Myanmar and Thailand [3]. Our study also allowed the identification of SEA4 subclade, including viruses from the Philippines previously associated with SEA2 subclade [3]. Finally, subclade SEA5 appears to be specific of RABV circulating in ferret-badgers, an epidemiological cycle that was recently identified in Taiwan [4-6].

The RABV from the Arctic-related clade (n=17) were representative of the subclades described previously [2], with subclades Arctic-like (AL) 1 including lineages 1a and 1b, AL2, AL3 and members of the Arctic (A) subclade.

Two clades of RABV appeared to be specific to Africa. The Africa-2 clade (n=19), includes RABV isolates from Central and Western Africa (Guinea, Benin, Cameroon, Central African Republic, Chad, Nigeria, Burkina-Faso, Niger, Senegal, Ivory Coast and Mauritania) as described previously [7] (Fig. S1B). Here, for the first time, we were able to fully characterize full-length genome sequences of RABV isolates belonging to the Africa-3 clade (n=6). These viruses circulating in Southern Africa are monophyletic and phylogenetically distinct from the other RABV major clades, in particular those circulating in Africa [8-10].

The Cosmopolitan clade (n=141) represents the largest group analyzed in terms of geographical distribution and phylogenetic diversity. Two phylogenetic subclades, Africa 1 (AF1) and Africa 4 (AF4), were clearly identified as circulating in the African continent. AF1 can be further subdivided in AF1a lineage including viruses from Algeria, Ethiopia, Gabon, Nigeria, Morocco and Somalia, AF1b lineage including viruses from Central African Republic, Kenya, Mozambique, Namibia, South Africa and Tanzania, and the AF1c lineage encompassing viruses from Madagascar, in accordance with previous work [11, 12]. The AF4 subclade comprises viruses from Africa (Egypt) and the Middle East (Israel), as initially described by David and colleagues [13].

Viruses belonging to the Cosmopolitan clade and circulating in the Americas are divided into four subclades, consistent with previous findings [14]: (i) the AM1 subclade including viruses circulating in skunks and woodchucks in the USA, (ii) the AM2 subclade containing viruses from Mexico that can be further subdivided into two lineages, AM2a and 2b, circulating in dog and wildlife, respectively, (iii) the AM3 subclade with lineages AM3a and 3b encompassing RABV infecting dog and foxes in Brazil, respectively, and (iv) the AM4 subclade including RABV circulating in skunks in California. The two vaccine strains SAD-B19 and PV were clustered together and were close to the AM1 subclade.

Three new subclades circulating in Central Asia (CA) were characterized here for the first time within the Cosmopolitan clade. These subclades encompass viruses originating in China, Iran and Russia for CA1, from Iraq, Iran and Russia for CA2, and a cluster of viruses that was named as CA3, although its precise geographical distribution (Hungary and Russia) at the border of Europe and Central Asia remains uncertain.

The analysis of RABV circulating in the Middle East confirms that they belong to the Cosmopolitan clade and can be divided into three subclades named AF4 (see above), Middle East 1 (ME1) and ME2, consistent with previous findings [13, 15]. ME1 corresponds to isolates from Israel, Iran, Saudi Arabia, Sultanate of Oman, and United Arab Emirates. ME2 includes isolates from Turkey.

Those RABV circulating in Europe all belonged to the Cosmopolitan clade. RABV isolates from foxes and raccoon-dogs were subdivided into four subclades, Central (CE), Eastern (EE), Western (WE) and North-Eastern (NEE) Europe, as described previously [16].

**Temporal dynamics and spread of the dog-related RABV**

Within the dog-related RABV, the Indian subcontinent clade appeared to diversify between 1733-1840 (mean of 1785). The TMRCA of the Asian clade was estimated to be between 1535-1677 (mean of 1604), which is in accordance with other studies [17, 18]. Similarly, the SEA1, 2, 3 and 4 subclades appeared around 1677-1795, 1830-1898, 1876-1920 and 1904-1946, respectively, and the most recent Asian subclade (SEA5) diverged from 1939-1973.

The emergence of the Africa 2 clade was estimated to be between 1750-1852 (mean of 1802), similar to the mean TMRCA found in a previous study conducted on complete N and G genes [7].

The Arctic-related clade appeared between 1725-1815 (mean of 1770), slightly earlier than previous estimates [2]. The TMRCA of subclades A, AL1, 2 and 3 were estimated to be between 1929-1954, 1901-1935, 1852-1921 and 1856-1906, respectively. These estimates differ from those obtained previously, particularly for AL1 and AL3, probably due to low number of samples for each subclade.

The Africa-3 clade emerged around between 1710-1815 (mean of 1756) in accordance with a previous study [10].

For the first time, we estimate that the TMRCA for the Cosmopolitan clade appeared between 1687-1773 (mean of 1730), with the emergence of the two African subclades AF1 and AF4 dated to approximately 1830-1877 and 1923-1940, respectively. Interestingly, the initial or a series of introductions is observed in three different geographic areas in America (Mexico, Brazil and California) around 1710-1790, which soon diverged into three subclades (AM2, 3, 4) and another in the North-Eastern territories of the present USA between 1767 and 1832 that led into the AM1 subclade. The subclades CA1, CA2, CA3 from Central Asia and ME1 and ME2 from Middle-East, emerged around 1936-1957, 1927-1960, 1926-1956, 1905-1933 and 1984-1989, respectively. Finally, the emergence of fox and raccoon dog rabies in Europe was estimated around 1877-1912, which is older than previously thought [16]. This first emergence occurs in the countries of North-Eastern Europe and most likely a second emergence gradually colonized the rest of Europe (central, south and west of Europe) between 1902 and 1929.

**References**

1. Bourhy H, Reynes JM, Dunham EJ, Dacheux L, Larrous F, Huong VT, et al. The origin and phylogeography of dog rabies virus. The Journal of general virology. 2008;89(Pt 11):2673-81. doi: 10.1099/vir.0.2008/003913-0. PubMed PMID: 18931062; PubMed Central PMCID: PMC3326349.

2. Pant GR, Lavenir R, Wong FY, Certoma A, Larrous F, Bhatta DR, et al. Recent emergence and spread of an Arctic-related phylogenetic lineage of rabies virus in Nepal. PLoS neglected tropical diseases. 2013;7(11):e2560. doi: 10.1371/journal.pntd.0002560. PubMed PMID: 24278494; PubMed Central PMCID: PMC3836727.

3. Guo Z, Tao X, Yin C, Han N, Yu J, Li H, et al. National borders effectively halt the spread of rabies: the current rabies epidemic in China is dislocated from cases in neighboring countries. PLoS neglected tropical diseases. 2013;7(1):e2039. doi: 10.1371/journal.pntd.0002039. PubMed PMID: 23383359; PubMed Central PMCID: PMC3561166.

4. Chang JC, Tsai KJ, Hsu WC, Tu YC, Chuang WC, Chang CY, et al. Rabies Virus Infection in Ferret Badgers (Melogale moschata subaurantiaca) in Taiwan: A Retrospective Study. Journal of wildlife diseases. 2015;51(4):923-8. doi: 10.7589/2015-04-090. PubMed PMID: 26267459.

5. Chiou HY, Hsieh CH, Jeng CR, Chan FT, Wang HY, Pang VF. Molecular characterization of cryptically circulating rabies virus from ferret badgers, Taiwan. Emerging infectious diseases. 2014;20(5):790-8. doi: 10.3201/eid2005.131389. PubMed PMID: 24751120; PubMed Central PMCID: PMC4012806.

6. Tsai KJ, Hsu WC, Chuang WC, Chang JC, Tu YC, Tsai HJ, et al. Emergence of a sylvatic enzootic formosan ferret badger-associated rabies in Taiwan and the geographical separation of two phylogenetic groups of rabies viruses. Veterinary microbiology. 2016;182:28-34. doi: 10.1016/j.vetmic.2015.10.030. PubMed PMID: 26711025.

7. Talbi C, Holmes EC, de Benedictis P, Faye O, Nakoune E, Gamatie D, et al. Evolutionary history and dynamics of dog rabies virus in western and central Africa. The Journal of general virology. 2009;90(Pt 4):783-91. doi: 10.1099/vir.0.007765-0. PubMed PMID: 19264663.

8. Davis PL, Rambaut A, Bourhy H, Holmes EC. The evolutionary dynamics of canid and mongoose rabies virus in Southern Africa. Archives of virology. 2007;152(7):1251-8. doi: 10.1007/s00705-007-0962-9. PubMed PMID: 17401615.

9. Nel LH, Sabeta CT, von Teichman B, Jaftha JB, Rupprecht CE, Bingham J. Mongoose rabies in southern Africa: a re-evaluation based on molecular epidemiology. Virus research. 2005;109(2):165-73. doi: 10.1016/j.virusres.2004.12.003. PubMed PMID: 15763147.

10. Van Zyl N, Markotter W, Nel LH. Evolutionary history of African mongoose rabies. Virus research. 2010;150(1-2):93-102. doi: 10.1016/j.virusres.2010.02.018. PubMed PMID: 20214938.

11. Andriamandimby SF, Heraud JM, Ramiandrasoa R, Ratsitorahina M, Rasambainarivo JH, Dacheux L, et al. Surveillance and control of rabies in La Reunion, Mayotte, and Madagascar. Vet Res. 2013;44:77. doi: 10.1186/1297-9716-44-77. PubMed PMID: 24016204; PubMed Central PMCID: PMCPMC3848982.

12. Kissi B, Tordo N, Bourhy H. Genetic polymorphism in the rabies virus nucleoprotein gene. Virology. 1995;209(2):526-37. doi: 10.1006/viro.1995.1285. PubMed PMID: 7778285.

13. David D, Hughes GJ, Yakobson BA, Davidson I, Un H, Aylan O, et al. Identification of novel canine rabies virus clades in the Middle East and North Africa. The Journal of general virology. 2007;88(Pt 3):967-80. doi: 10.1099/vir.0.82352-0. PubMed PMID: 17325371.

14. Kuzmin IV, Shi M, Orciari LA, Yager PA, Velasco-Villa A, Kuzmina NA, et al. Molecular inferences suggest multiple host shifts of rabies viruses from bats to mesocarnivores in Arizona during 2001-2009. PLoS pathogens. 2012;8(6):e1002786. doi: 10.1371/journal.ppat.1002786. PubMed PMID: 22737076; PubMed Central PMCID: PMC3380930.

15. David D, Dveres N, Yakobson BA, Davidson I. Emergence of dog rabies in the northern region of Israel. Epidemiology and infection. 2009;137(4):544-8. doi: 10.1017/S0950268808001180. PubMed PMID: 18796169.

16. Bourhy H, Kissi B, Audry L, Smreczak M, Sadkowska-Todys M, Kulonen K, et al. Ecology and evolution of rabies virus in Europe. The Journal of general virology. 1999;80 ( Pt 10):2545-57. doi: 10.1099/0022-1317-80-10-2545. PubMed PMID: 10573146.

17. Gong W, Jiang Y, Za Y, Zeng Z, Shao M, Fan J, et al. Temporal and spatial dynamics of rabies viruses in China and Southeast Asia. Virus research. 2010;150(1-2):111-8. doi: 10.1016/j.virusres.2010.02.019. PubMed PMID: 20214936.

18. Zieger U, Marston DA, Sharma R, Chikweto A, Tiwari K, Sayyid M, et al. The phylogeography of rabies in Grenada, West Indies, and implications for control. PLoS neglected tropical diseases. 2014;8(10):e3251. doi: 10.1371/journal.pntd.0003251. PubMed PMID: 25330178; PubMed Central PMCID: PMCPMC4199513.
