# Supplementary figures and images for: Large-Scale Phylogenomic Analysis Reveals the Complex Evolutionary History of Rabies Virus in Multiple Carnivore Hosts
Source: PLoS Pathog. 2016 Dec 15;12(12):e1006041. doi: 10.1371/journal.ppat.1006041 (PMC5158080; doi:10.1371/journal.ppat.1006041)

A

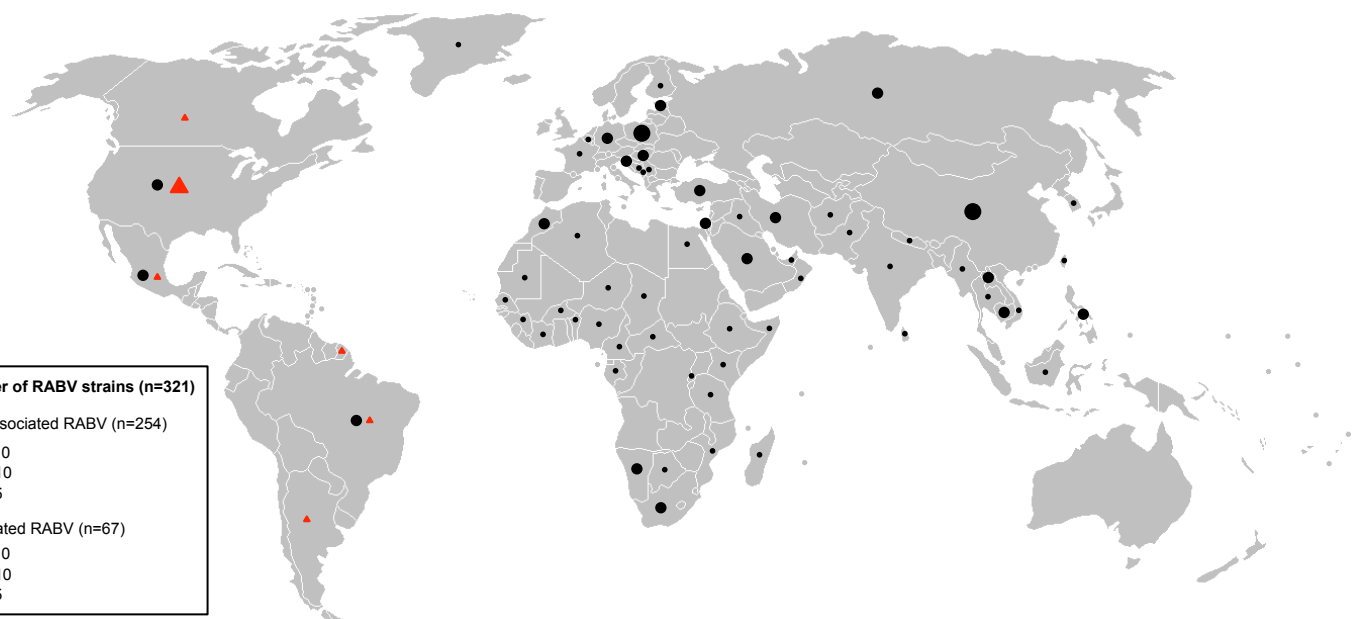

B

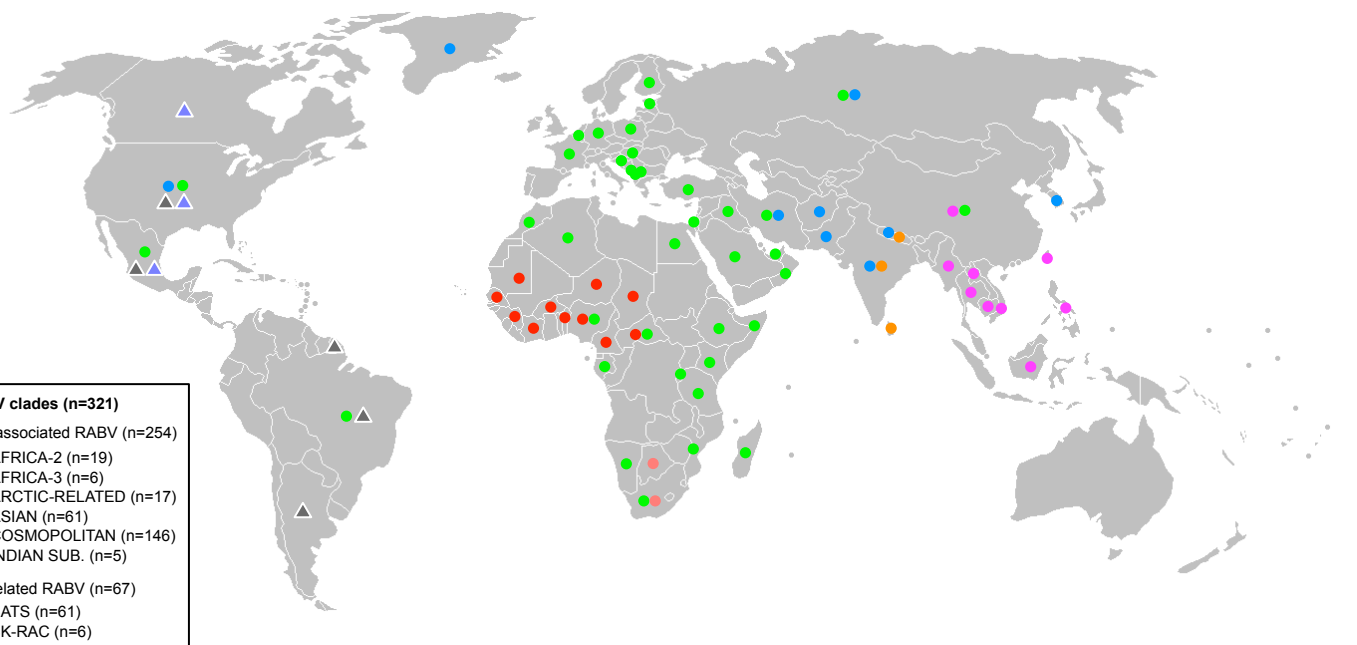

Supplement: S1 Fig — (A) Triangles and dots represent the bat- and dog-related RABV, respectively, with sizes proportional to the number of isolates as indicated in the legend. (B) The different colors represent the major clades in the bat- and dog-related RABV groups. (PDF) [file ppat.1006041.s002.pdf]

[illegible]

C

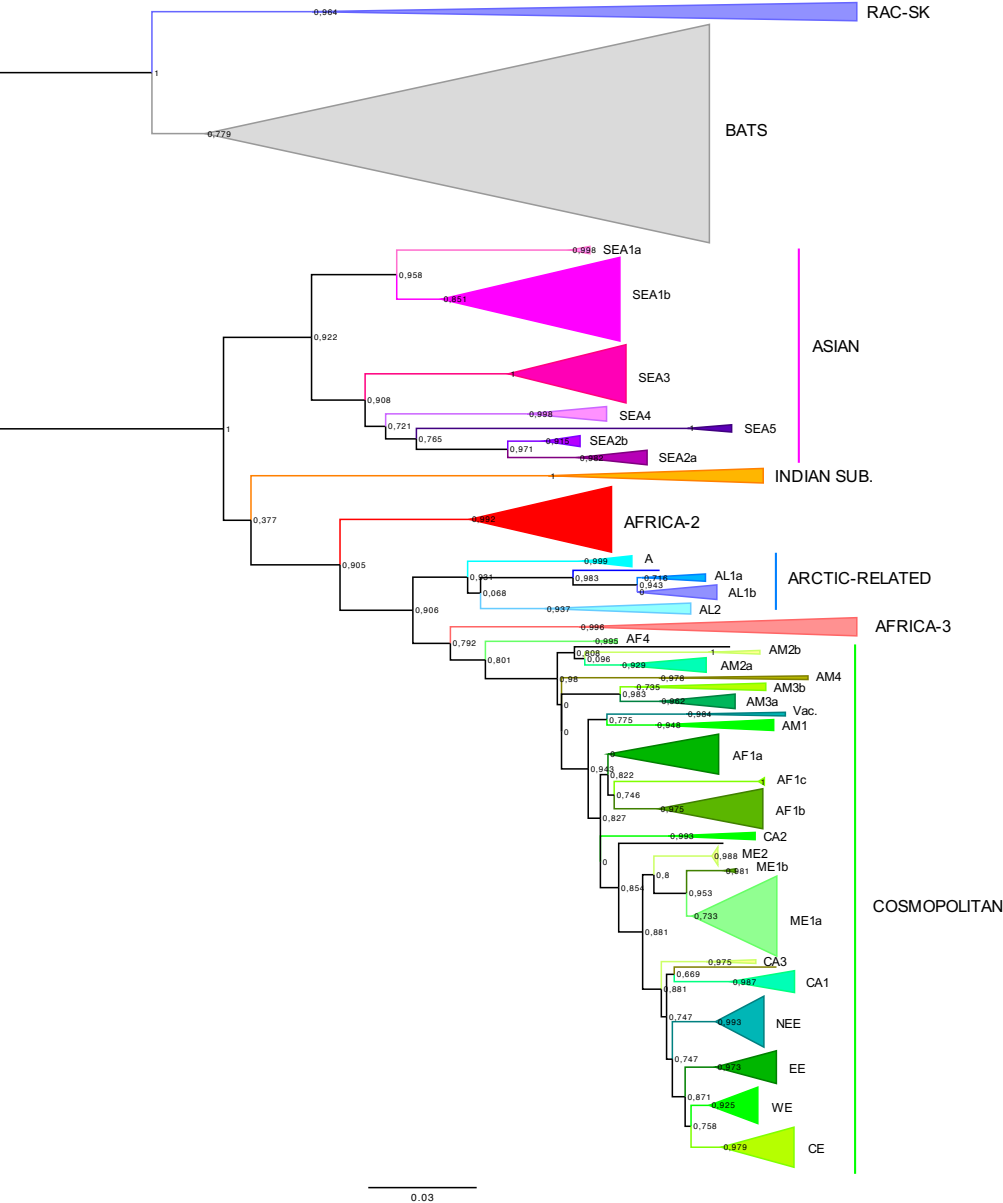

D

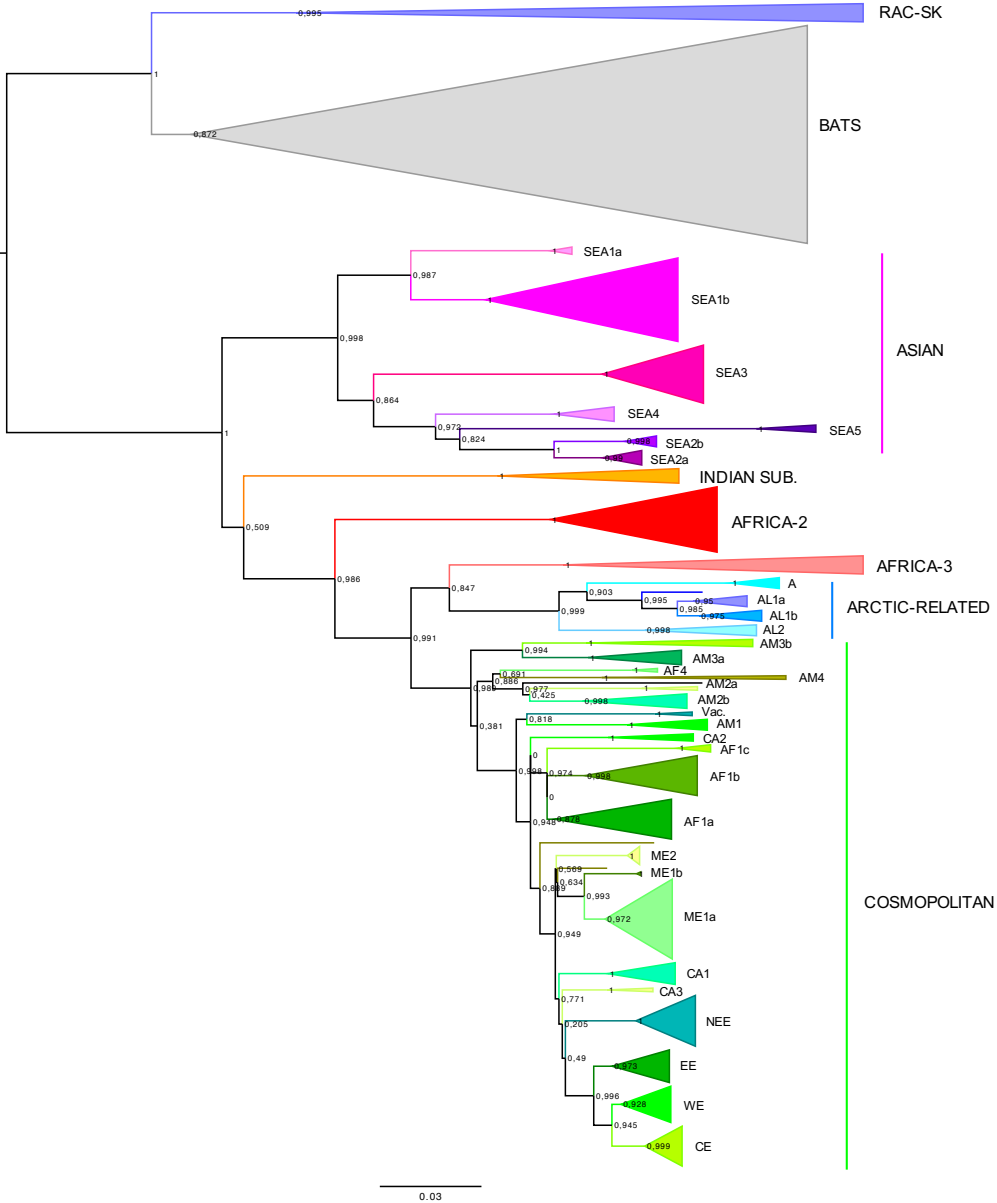

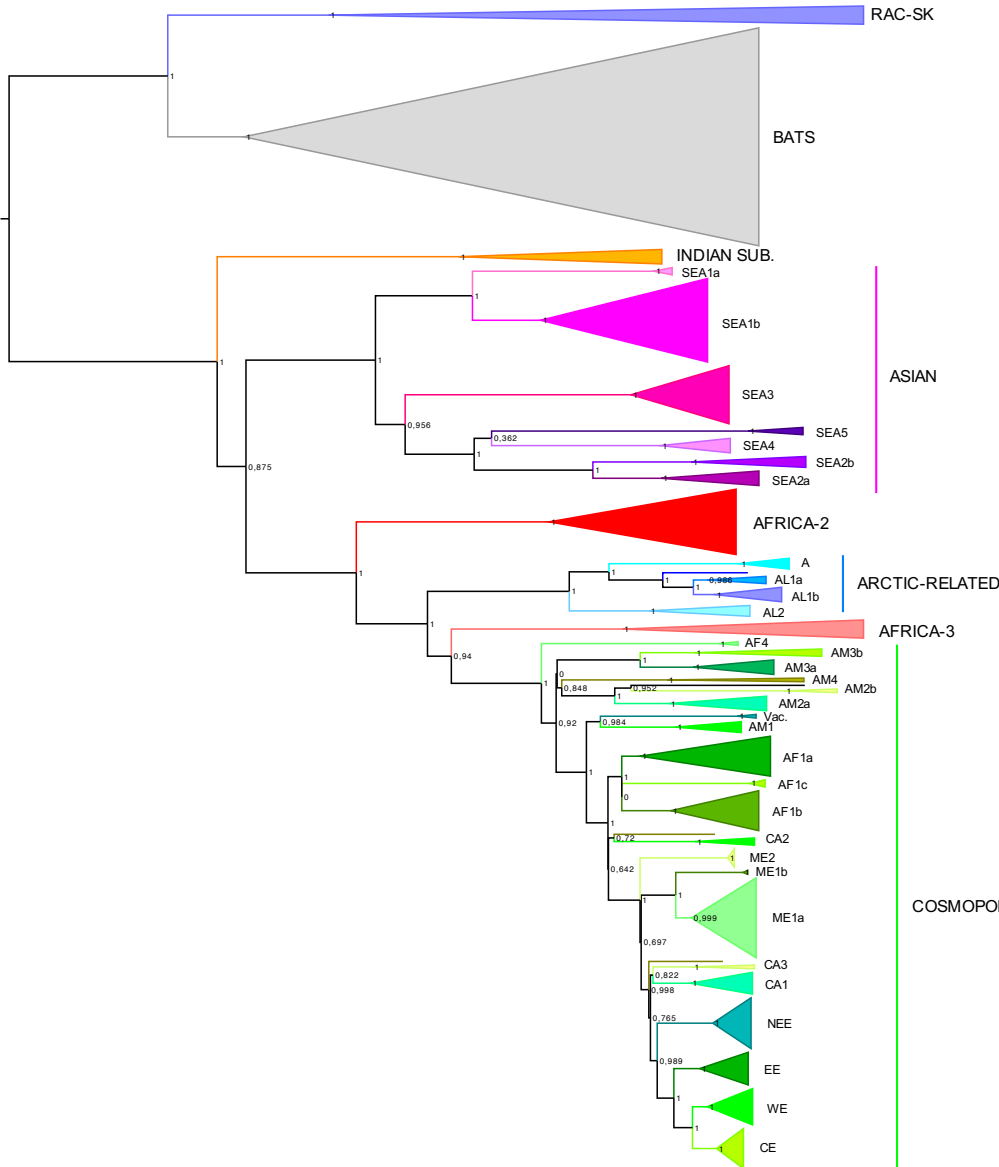

0.03

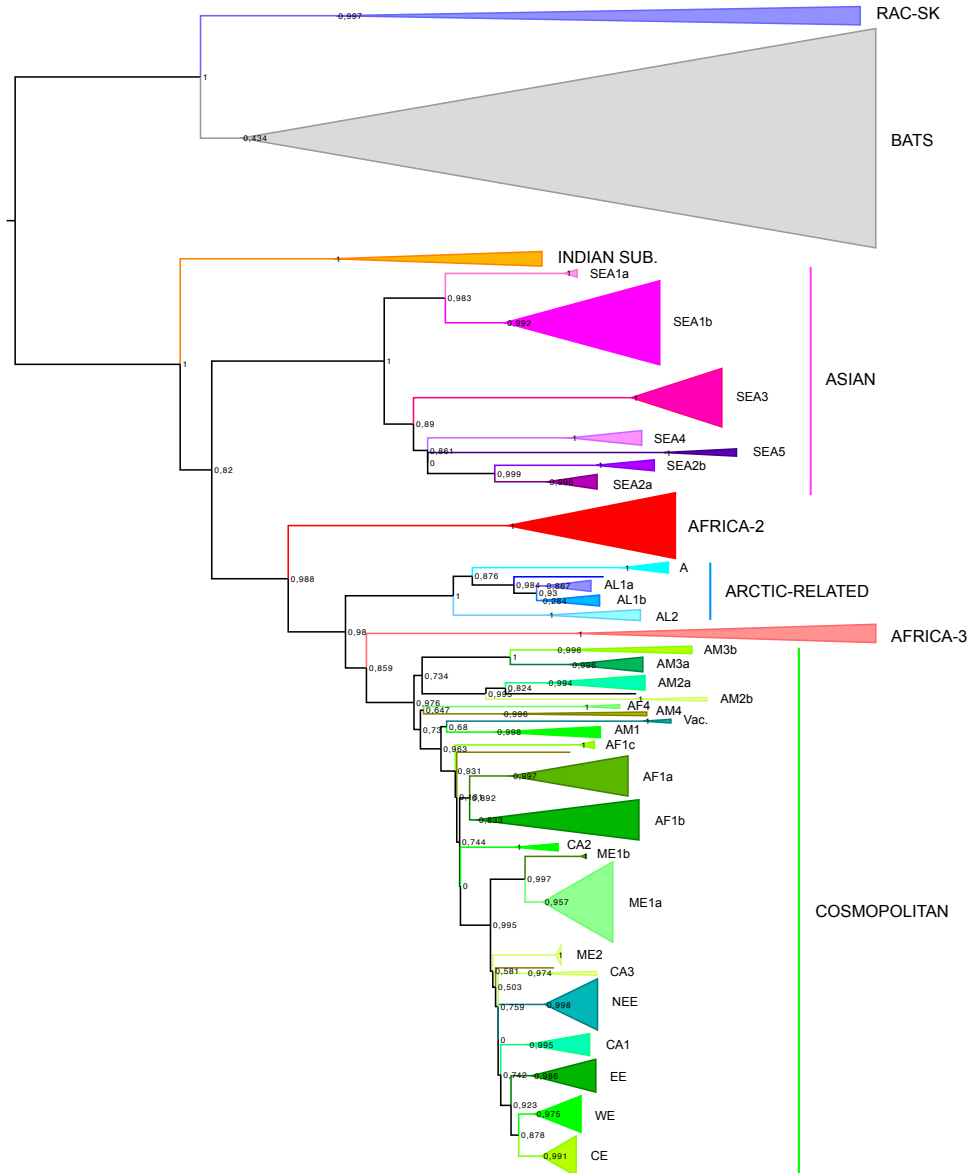

0.04

Supplement: S2 Fig — The ML trees are mid-point rooted and aLRT values are shown for each clade, subclade and lineage named according to Fig 1 and the nucleoprotein (A), phosphoprotein (B), matrix (C), glycoprotein (D), polymerase (E) genes and concatenated non-coding regions (F) are shown separately. (PDF) [file ppat.1006041.s003.pdf]

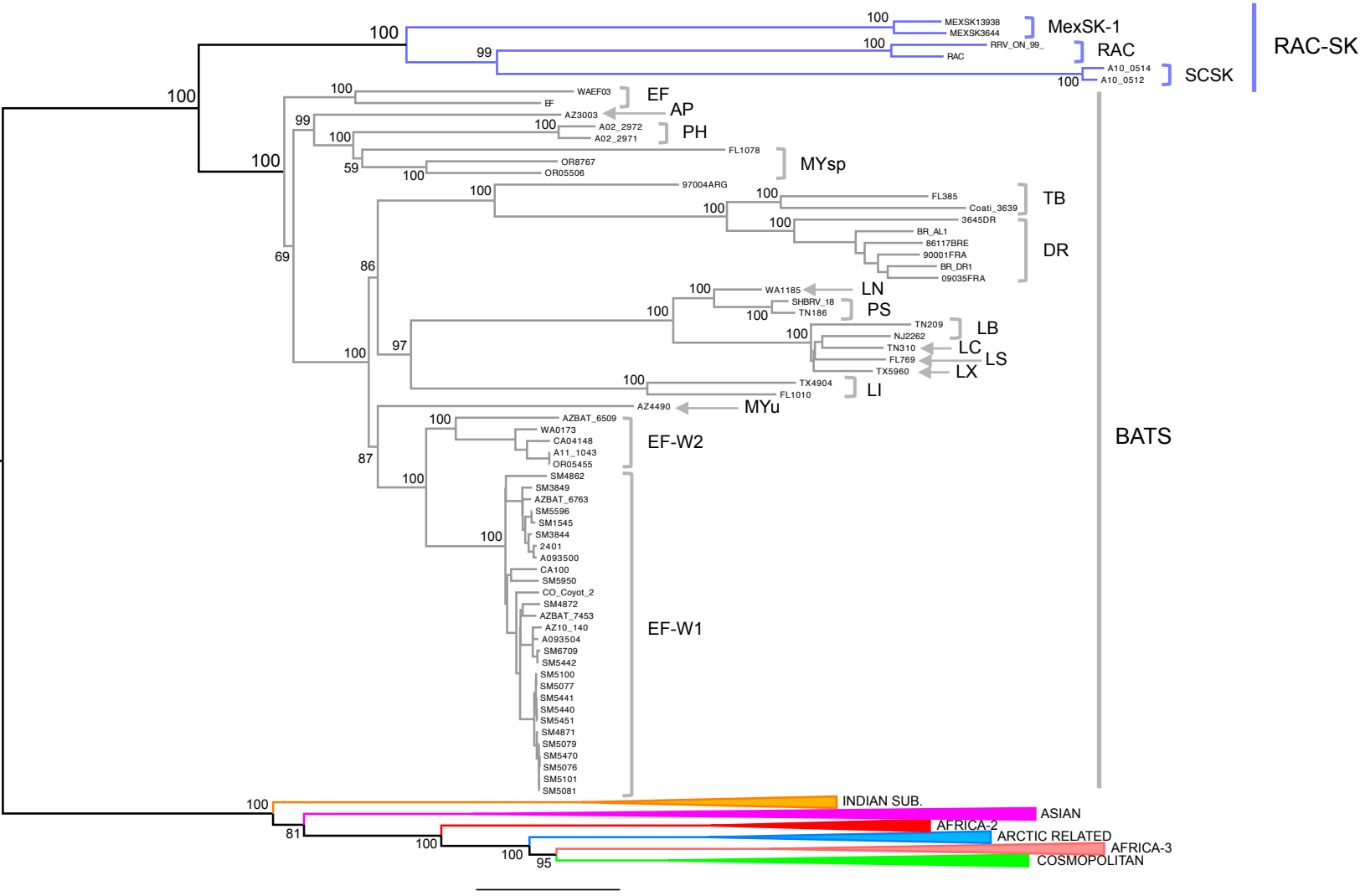

Supplement: S3 Fig — The tree is mid-point rooted with bootstrap values shown for each major subclades. The subclade names are given according to Kuzmin et al., (2012) [14]. Subclade abbreviations: SCSK–South-Central skunk; RAC–North-American Raccoon; MexSK-1 –Mexican skunk, variant 1; EF-W1 and EF-W2 –Eptesicus fuscus, in western USA; MYu–Myotis yumanensis; LX–Lasiurus xanthinus; LS–Lasiurus seminolus; LC–Lasiurus cinereus; LB–Lasiurus borealis; PS–Perimyotis subflavus; LN–Lasionycteris noctivagans; LI–Lasiurus intermedius; TB–Tadarida brasiliensis; DR–Desmodus rotundus; MYsp–Myotis spp; PH–Parastrellus hesperus; AP–Antrozous pallidus; EF–Eptesicus fuscus, in eastern and central USA. (PDF) [file ppat.1006041.s004.pdf]

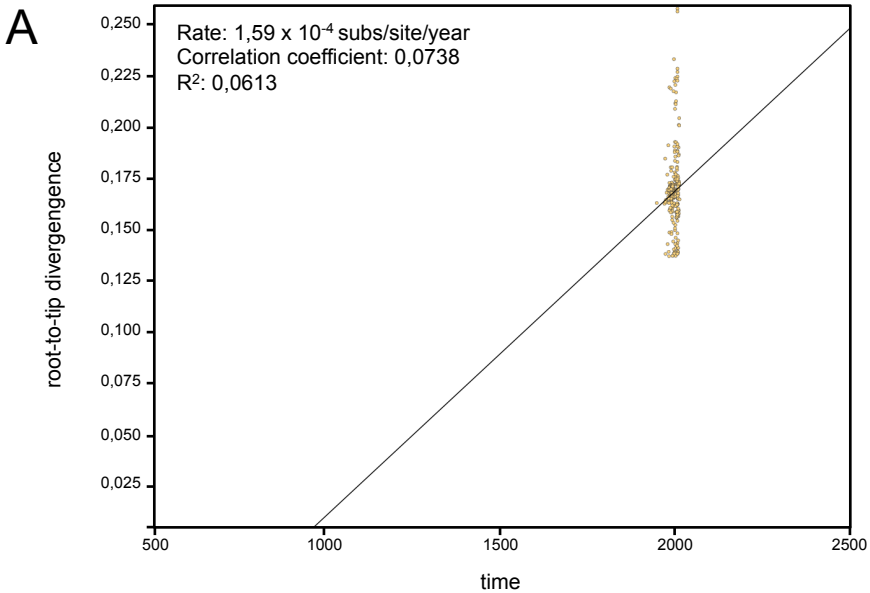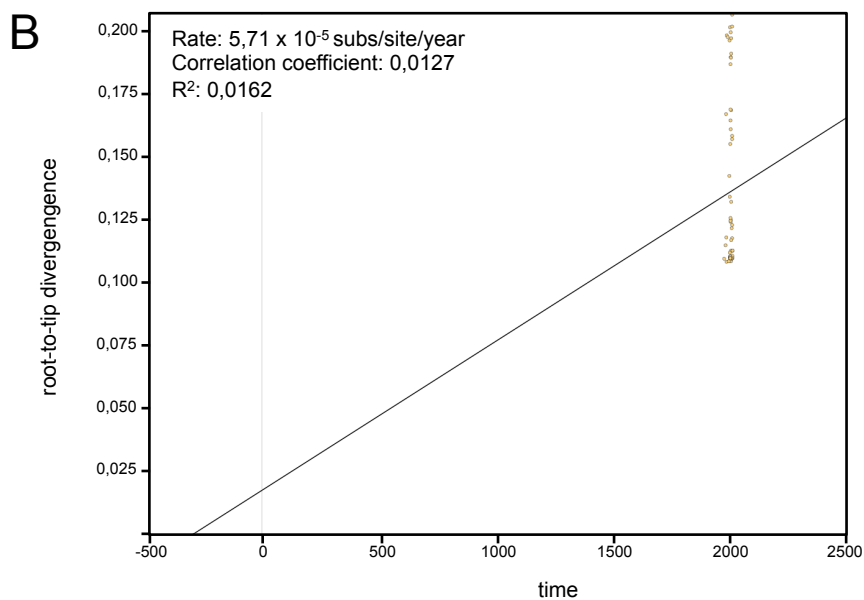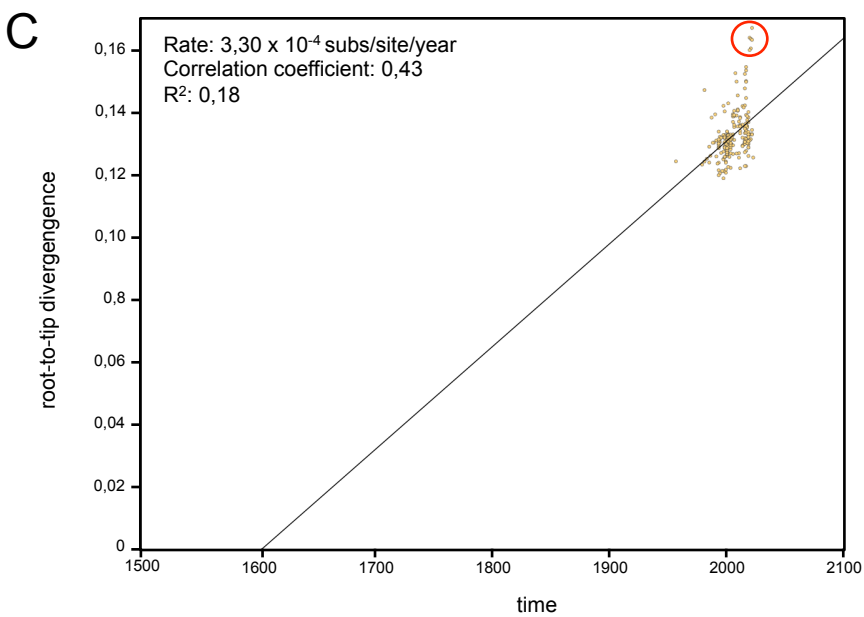

Supplement: S4 Fig — The root-to-tip regressions were obtained using TempEst [80], on (A) a combined bat- and dog-related RABV data set (n = 315), (B) bat-related RABV data set (n = 67), and (C) dog-related RABV data set (n = 248) (isolates for which the date of sampling were unavailable and vaccine strains were excluded). The red circle indicates a number of outlier strains characterized by anomalously high rates (employed an arbitrary cut-off). The inferred rate of nucleotide substitution corresponding to the slope and the correlation coefficient are also indicated. (PDF) [file ppat.1006041.s005.pdf]

COSMOPOLITAN

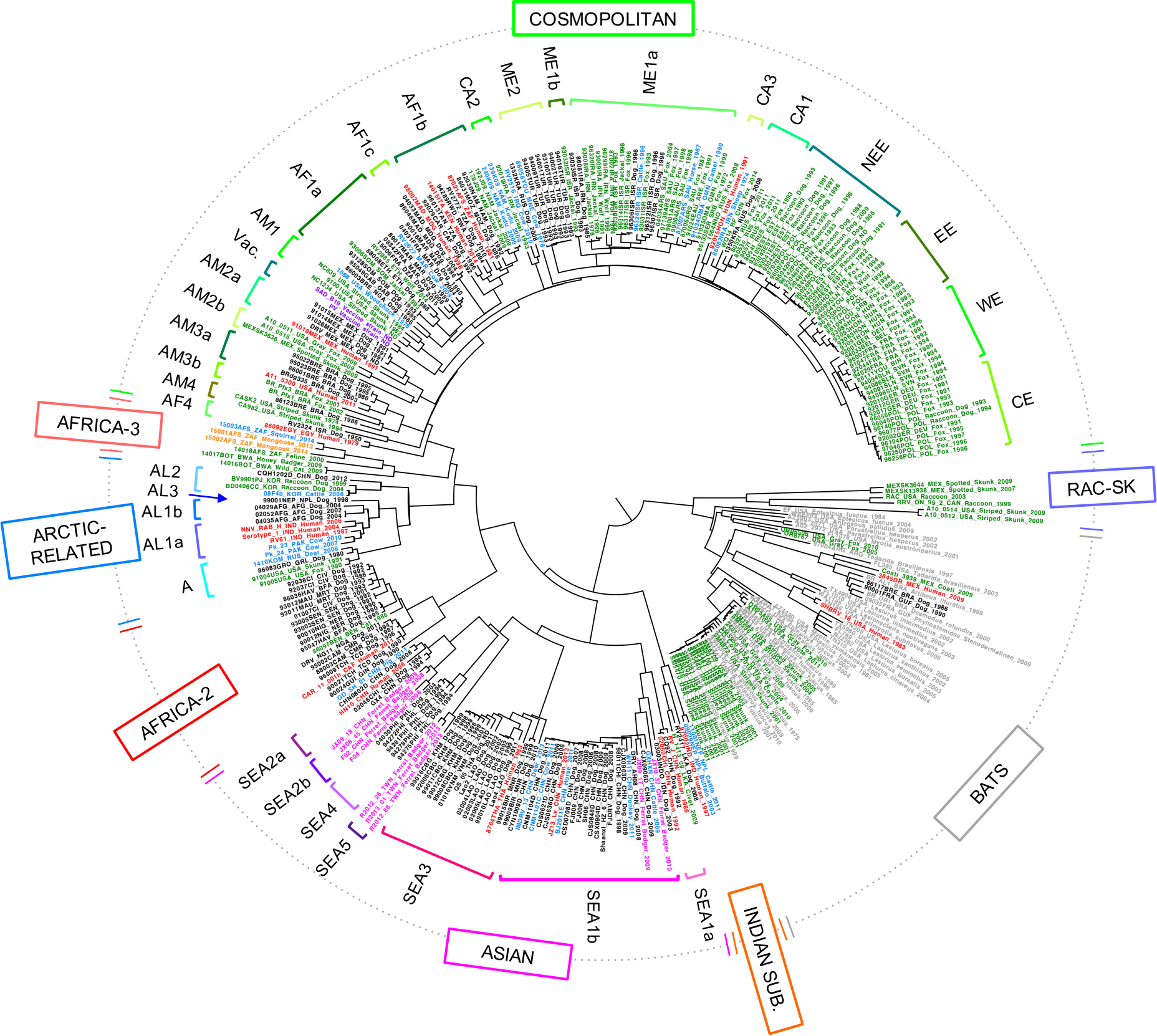

Supplement: S5 Fig — Tip names are colored according to the isolation species of each virus, dog in black, bat in grey, ferret-badger in magenta, mongoose in orange, human in red, other carnivores in green, herbivores and/or omnivores in blue and vaccine strains in purple. The major clades of RABV are indicated in boxes like in Fig 1. The names of subclades and lineages defined for the Arctic-related, Asian and Cosmopolitan clades are detailed in S1 Table. The tree is mid-point rooted for clarity only. (PDF) [file ppat.1006041.s006.pdf]
