# Supplementary material for: Large-Scale Phylogenomic Analysis Reveals the Complex Evolutionary History of Rabies Virus in Multiple Carnivore Hosts
Source: PLoS Pathog. 2016 Dec 15;12(12):e1006041. doi: 10.1371/journal.ppat.1006041 (PMC5158080; doi:10.1371/journal.ppat.1006041)
Supplement: S1 Table — (DOCX) [file ppat.1006041.s007.docx]

**Table S1: List of viruses used in the full-length genome analyses.**

| **Country** | **Virus Name** | **Species** | **Year** | **Phylogenetic clade - subclade** | **Source** | **Accession number** | **Reference** |
| --- | --- | --- | --- | --- | --- | --- | --- |
| Afghanistan | 02052AFG | Dog  (*Canis familiaris*) | 2002 | Arctic-related – AL1b | Original brain | KX148225^a^ | This study |
| Afghanistan | 04029AFG | Dog  (*Canis familiaris*) | 2004 | Arctic-related – AL1b | Original brain | KX148227^a^ | This study |
| Afghanistan | 04035AFG | Dog  (*Canis familiaris*) | 2002 | Arctic-related – AL1b | Original brain | KX148226^a^ | This study |
| Algeria | 15006FRA | Dog  (*Canis familiaris*) | 2015 | Cosmopolitan – AF1a | Original brain | KX148197^b^ | This study |
| Argentina | 97004ARG | Bat  (*Tadarida brasiliensis*) | 1997 | Bat - TB | Mouse | KX148269^c^ | This study |
| Belgium | 94033BEL | Red fox  (*Vulpes vulpes*) | 1994 | Cosmopolitan - WE | Original brain | KX148125^b^ | This study |
| Benin | 86097BEN | Cat  *(Felis catus)* | 1986 | Africa-2 | Original brain | KX148107^d^ | This study |
| Bosnia and Herzegovina | 86054YOU | Wolf  *(Canis lupus)* | 1986 | Cosmopolitan - EE | Mouse | KX148145^e^ | This study |
| Bosnia and Herzegovina | 86111YOU | Red fox  (*Vulpes vulpes*) | 1986 | Cosmopolitan - WE | Mouse | KX148133^f^ | This study |
| Botswana | 14016BOT | Wildcat  (*Felis silvestris*) | 2009 | Africa-3 | Original brain | KX148218^e^ | This study |
| Botswana | 14017BOT | Honey badger  *(Mellivora capensis)* | 2009 | Africa-3 | Original brain | KX148219^e^ | This study |
| Brazil | 86001BRE | Dog  (*Canis familiaris*) | 1986 | Cosmopolitan – AM3a | Original brain | KX148216^e^ | This study |
| Brazil | 86117BRE | Dog  (*Canis familiaris*) | 1986 | Bat - DR | Original brain | KX148109^d^ | This study |
| Brazil | 86123BRE | Dog  (*Canis familiaris*) | 1986 | Cosmopolitan – AM3b | Mouse | KX148217^f^ | This study |
| Brazil | 95022BRE | Dog  (*Canis familiaris*) | 1995 | Cosmopolitan – AM3a | Original brain | KX148215^a^ | This study |
| Brazil | 95023BRE | Dog  (*Canis familiaris*) | 1995 | Cosmopolitan – AM3a | Original brain | KX148214^f^ | This study |
| Brazil | BR_AL1 | Bat  (*Artibeus lituratus*) | 1998 | Bat - DR | Original brain | AB519641 | (1) |
| Brazil | BR_DR1 | Bat  (*Desmodus rotundus*) | 2000 | Bat - DR | Original brain | AB519642 | (1) |
| Brazil | BR_Pfx1 | Brazilian wild fox  (*Dusicyon*_sp.) | 2002 | Cosmopolitan – AM3b | Original brain | AB362483 | (2) |
| Brazil | BR_Pfx3 | Brazilian wild fox  (*Dusicyon*_sp.) | 2001 | Cosmopolitan – AM3b | ? | AB517660 | Mochizuki *et al.,* unpublished |
| Brazil | BRdg335 | Dog  (*Canis familiaris*) | 2003 | Cosmopolitan – AM3a | ? | AB517659 | Mochizuki *et al.,* unpublished |
| Burkina Faso | 86036HAV | Dog  (*Canis familiaris*) | 1986 | Africa-2 | Mouse | KX148234^e^ | This study |
| Burkina Faso | 95047HAV | Dog  (*Canis familiaris*) | 1995 | Africa-2 | Original brain | KX148230^a^ | This study |
| Cambodia | 02006CBG | Dog  (*Canis familiaris*) | 1998 | Asian – SEA3 | Original brain | KX148249^a^ | This study |
| Cambodia | 99008CBG | Dog  (*Canis familiaris*) | 1999 | Asian – SEA3 | Original brain | KX148252^a^ | This study |
| Cambodia | 99011CBG | Dog  (*Canis familiaris*) | 1998 | Asian – SEA3 | Original brain | KX148251^a^ | This study |
| Cambodia | 99012CBG | Dog  (*Canis familiaris*) | 1998 | Asian – SEA3 | Original brain | KX148253^a^ | This study |
| Cambodia | 99016CBG | Dog  (*Canis familiaris*) | 1999 | Asian – SEA3 | Original brain | KX148250^a^ | This study |
| Cameroon | 88003CAM | Dog  (*Canis familiaris*) | 1987 | Africa-2 | Original brain | KX148243^a^ | This study |
| Cameroon | 95002CAM | Dog  (*Canis familiaris*) | 1994 | Africa-2 | Original brain | KX148242^a^ | This study |
| Canada | RRV_ON_99 | Raccoon  *(Procyon lotor)* | 1999 | RAC-SK – RAC | Original brain | EU311738 | (3) |
| Central African Republic | 92029CAR | Dog  (*Canis familiaris*) | 1992 | Cosmopolitan – AF1b | Original brain | KX148208^a^ | This study |
| Central African Republic | CAR_11_001 | Human  (*Homo sapiens*) | 2011 | Africa-2 | Saliva | KF977826 | (4) |
| Chad | 90021TCH | Dog  (*Canis familiaris*) | 1990 | Africa-2 | Original brain | KX148240^a^ | This study |
| Chad | 96009TCH | Dog  (*Canis familiaris*) | 1996 | Africa-2 | Original brain | KX148241^a^ | This study |
| China | 02046CHI | Dog  (*Canis familiaris*) | 1994 | Asian – SEA2a | Mouse | KX148264^e^ | This study |
| China | 02050CHI | Human  (*Homo sapiens*) | 1992 | Asian – SEA1a | Mouse | KX148267^e^ | This study |
| China | 98011CHI | Dog  (*Canis familiaris*) | 1998 | Asian – SEA1b | Original brain | KX148265^a^ | This study |
| China | JX09_18 | Ferret badger  *(Melogale moschata)* | 2009 | Asian – SEA2b | Original brain | KF726852 | (5) |
| China | JX10_37 | Ferret badger  *(Melogale moschata)* | 2010 | Asian – SEA1b | Original brain | KF726853 | (5) |
| China | JX08_45 | Ferret badger  *(Melogale moschata)* | 2008 | Asian – SEA2b | ? | GU647092 | Zhao *et al.,* unpublished |
| China | JX09_17 | Ferret badger  *(Melogale moschata)* | 2009 | Asian – SEA1b | Original brain | KC762941 | (6) |
| China | BJ2011E | Horse  (*Equus ferus*) | 2011 | Asian – SEA1b | ? | JQ423952 | Chen *et al.,* unpublished |
| China | CHN0802D | Dog  (*Canis familiaris*) | 2008 | Asian – SEA2a | Original brain | JQ970480 | Tang *et al.,* unpublished |
| China | CJS0621D | Dog  (*Canis familiaris*) | 2006 | Asian – SEA1b | Original brain | JQ970481 | Tang *et al.,* unpublished |
| China | CJS0636D | Dog  (*Canis familiaris*) | 2006 | Asian – SEA1b | Original brain | JQ970482 | Tang *et al.,* unpublished |
| China | CJS0848D | Dog  (*Canis familiaris*) | 2008 | Asian – SEA1b | Original brain | JQ970483 | Tang *et al.,* unpublished |
| China | CJX0903D | Dog  (*Canis familiaris*) | 2009 | Asian – SEA1b | Original brain | JQ970484 | Tang *et al.,* unpublished |
| China | CJX0906D | Dog  (*Canis familiaris*) | 2009 | Asian – SEA1b | Original brain | JQ970485 | Tang *et al.,* unpublished |
| China | CNM1101C | Cow  (*Bos taurus*) | 2011 | Asian – SEA1b | Original brain | KC193267 | Yin *et al.,* unpublished |
| China | CNM1104D | Dog  (*Canis familiaris*) | 2011 | Asian – SEA1b | Original brain | KC252634 | Yin *et al.,* unpublished |
| China | CQ92 | Dog  (*Canis familiaris*) | 1992 | Asian – SEA1a | ? | GU345746 | (7) |
| China | CQH1202D | Dog  (*Canis familiaris*) | 2012 | Arctic-related – AL2 | ? | KM272192 | Guo *et al.,* unpublished |
| China | CSD0708D | Dog  (*Canis familiaris*) | 2007 | Asian – SEA1b | Original brain | JQ970486 | Tang *et al.,* unpublished |
| China | CSX0904D | Dog  (*Canis familiaris*) | 2009 | Asian – SEA1b | Original brain | JQ970487 | Tang *et al.,* unpublished |
| China | CYN1009D | Dog  (*Canis familiaris*) | 2010 | Asian – SEA3 | Original brain | JQ730682 | (8) |
| China | DRV_AH08 | Dog  (*Canis familiaris*) | 2008 | Asian – SEA2b | Mouse | HQ450385 | (9) |
| China | F02 | Ferret badger  *(Melogale moschata)* | 2008 | Asian – SEA2b | Original brain | FJ712195 | Wang *et al.,* unpublished |
| China | F04 | Ferret badger  *(Melogale moschata)* | 2008 | Asian – SEA1b | Original brain | J712196 | Wang *et al.,* unpublished |
| China | FJ008 | Dog  (*Canis familiaris*) | 2008 | Asian – SEA1b | ? | FJ866835 | Zhang *et al.,* unpublished |
| China | FJ009 | Dog  (*Canis familiaris*) | 2008 | Asian – SEA1b | ? | FJ866836 | Zhang *et al.,* unpublished |
| China | FJDRV | Dog  (*Canis familiaris*) | 2008 | Asian – SEA1b | ? | JN609295 | Qi, unpublished |
| China | GD_SH_01 | Pig  *(Sus domesticus)* | 2011 | Asian – SEA2a | Original brain | JX088694 | (10) |
| China | GX4 | Dog  (*Canis familiaris*) | 1994 | Asian – SEA2a | ? | GU358653 | (7) |
| China | GXHXN | Cattle  (*Bos taurus*) | 2009 | Asian – SEA1b | Original brain | KC169986 | (11) |
| China | HN10 | Human  (*Homo sapiens*) | 2006 | Asian – SEA2a | ? | EU643590 | (12) |
| China | IMDRV_13 | Fallow deer  (*Dama dama*) | 2013 | Asian – SEA1b | Original brain | KJ564280 | (13) |
| China | JZ13_Lv | Human  (*Homo sapiens*) | 2013 | Asian – SEA1b | ? | KJ004416 | Wang *et al.,* unpublished |
| China | RV_J | Human  (*Homo sapiens*) | 1986 | Asian – SEA1a | ? | GU345747 | (7) |
| China | SH06 | Dog  (*Canis familiaris*) | 2006 | Asian – SEA1b | ? | GU345748 | (7) |
| China | WH11 | Donkey  *(Equus asinus)* | 2011 | Asian – SEA1b | Original brain | JQ647510 | (14) |
| China | WQ14_RF | Red fox  (*Vulpes vulpes*) | 2014 | Cosmopolitan – CA1 | Original brain | KM016899 | Liu *et al.,* unpublished |
| China | Shaanxi_HZ | Dog  (*Canis familiaris*) | 2009 | Asian – SEA1b | ? | KC977995 | (15) |
| Egypt | 86092EGY | Human  (*Homo sapiens*) | 1979 | Cosmopolitan – AF4 | Mouse | KX148101^d^ | This study |
| Estonia | 93038EST | Red fox  (*Vulpes vulpes*) | 1992 | Cosmopolitan – NEE | Original brain | KX148148^a^ | This study |
| Estonia | 93039EST | Raccoon dog  *(Nyctereutes procyonoides)* | 1991 | Cosmopolitan – NEE | Original brain | KX148149^a^ | This study |
| Estonia | 93041EST | Red fox  (*Vulpes vulpes*) | 1993 | Cosmopolitan – NEE | Original brain | KX148155^a^ | This study |
| Estonia | 93042EST | Raccoon dog  *(Nyctereutes procyonoides)* | 1991 | Cosmopolitan – NEE | Original brain | KX148156^a^ | This study |
| Estonia | 93043EST | Raccoon dog  *(Nyctereutes procyonoides)* | 1993 | Cosmopolitan – NEE | Original brain | KX148157^a^ | This study |
| Estonia | 93105EST | Red fox  (*Vulpes vulpes*) | 1993 | Cosmopolitan – NEE | Original brain | KX148158^c^ | This study |
| Estonia | RV437 | Raccoon dog  *(Nyctereutes procyonoides)* | ND | Cosmopolitan – NEE | Mouse | KF154997 | (16) |
| Ethiopia | 88008ETH | Dog  (*Canis familiaris*) | 1988 | Cosmopolitan – AF1a | Original brain | KX148200^a^ | This study |
| Ethiopia | RV2985 | Ethiopian wolf  (*Canis simensis*) | 2014 | Cosmopolitan – AF1a | Original brain | KP723638 | (17) |
| Finland | 93046FIN | Red fox  (*Vulpes vulpes*) | 1993 | Cosmopolitan – NEE | Original brain | KX148146^a^ | This study |
| Finland | 93048FIN | Raccoon dog  *(Nyctereutes procyonoides)* | 1988 | Cosmopolitan – NEE | Original brain | KX148147^a^ | This study |
| France | 91047FRA | Red fox  (*Vulpes vulpes*) | 1991 | Cosmopolitan – WE | Original brain | KX148127^a^ | This study |
| France | 92044FRA | Red fox  (*Vulpes vulpes*) | 1992 | Cosmopolitan – WE | Original brain | KX148128^a^ | This study |
| France | 94029FRA | Red fox  (*Vulpes vulpes*) | 1994 | Cosmopolitan – WE | Original brain | KX148134^a^ | This study |
| France | 96002FRA | Red fox  (*Vulpes vulpes*) | 1996 | Cosmopolitan – WE | Original brain | KX148126^a^ | This study |
| French Guiana | 09035FRA | Bat  (*Phyllostomidae stenodermatinae*) | 2009 | Bat - DR | Original brain | KX148100^g^ | This study |
| French Guiana | 90001FRA | Dog  (*Canis familiaris*) | 1990 | Bat - DR | Original brain | KX148268^b^ | This study |
| Gabon | 95049GAB | Dog  (*Canis familiaris*) | 1995 | Cosmopolitan – AF1a | Original brain | KX148202^a^ | This study |
| Germany | 92001GER | Red fox  (*Vulpes vulpes*) | 1991 | Cosmopolitan – WE | Original brain | KX148135^a^ | This study |
| Germany | 92002GER | Red fox  (*Vulpes vulpes*) | 1991 | Cosmopolitan – CE | Original brain | KX148113^a^ | This study |
| Germany | 92010GER | Red fox  (*Vulpes vulpes*) | 1991 | Cosmopolitan – CE | Original brain | KX148124^a^ | This study |
| Germany | 92012GER | Red fox  (*Vulpes vulpes*) | 1991 | Cosmopolitan – CE | Original brain | KX148122^a^ | This study |
| Germany | 92017GER | Red fox  (*Vulpes vulpes*) | 1991 | Cosmopolitan – CE | Original brain | KX148123^a^ | This study |
| Greenland | 86083GRO | Dog  (*Canis familiaris*) | 1980 | Arctic-related – A | Mouse | KX148105^d^ | This study |
| Guinea | 90024GUI | Dog  (*Canis familiaris*) | 1990 | Africa-2 | Original brain | KX148244^a^ | This study |
| Hungary | 92015HON | Human  (*Homo sapiens*) | 1991 | Cosmopolitan – CA3 | Original brain | KX148160^e^ | This study |
| Hungary | 93080HON | Red fox  (*Vulpes vulpes*) | 1993 | Cosmopolitan – EE | Original brain | KX148143^a^ | This study |
| Hungary | 93083HON | Red fox  (*Vulpes vulpes*) | 1993 | Cosmopolitan – EE | Original brain | KX148136^a^ | This study |
| Hungary | 93089HON | Red fox  (*Vulpes vulpes*) | 1993 | Cosmopolitan – EE | Original brain | KX148137^a^ | This study |
| Hungary | 93091HON | Red fox  (*Vulpes vulpes*) | 1993 | Cosmopolitan – EE | Original brain | KX148138^a^ | This study |
| Hungary | 93093HON | Red fox  (*Vulpes vulpes*) | 1993 | Cosmopolitan – EE | Original brain | KX148139^a^ | This study |
| India | 97002IND | Human  (*Homo sapiens*) | 1997 | Indian Subcontinent | Original brain | KX148246^b^ | This study |
| India | NNV_RAB_H | Human  (*Homo sapiens*) | 2006 | Arctic-related – AL1a | Original brain | EF437215 | Desai *et al.,* unpublished |
| India | RV61 | Human  (*Homo sapiens*) | 1987 | Arctic-related – AL1a | Mouse | KF154996 | (16) |
| India | Serotype_1 | Human  (*Homo sapiens*) | 2004 | Arctic-related – AL1a | Saliva? | AY956319 | Pfefferle *et al.,* unpublished |
| Indonesia | 03003INDO | Dog  (*Canis familiaris*) | 2003 | Asian – SEA1a | Mouse | KX148266^c^ | This study |
| Irak | RV2516 | Cow  (*Bos taurus*) | 2010 | Cosmopolitan – CA2 | Original brain | KF155000 | (16) |
| Iran | 86081IRA | Dog  (*Canis familiaris*) | 1985 | Cosmopolitan – ME1a | Mouse | KX148189^f^ | This study |
| Iran | 86082IRA | Sheep  *(Ovis aries)* | 1974 | Cosmopolitan – CA1 | Mouse | KX148159^e^ | This study |
| Iran | 87002IRA | Wolf  *(Canis pallipes)* | 1984 | Cosmopolitan – ME1a | Mouse | KX148186^f^ | This study |
| Iran | 93008IRA | Jackal  *(Canis aureus)* | 1976 | Cosmopolitan – ME1a | Original brain | KX148190^a^ | This study |
| Iran | 93009IRA | Wolf  *(Canis pallipes)* | 1991 | Cosmopolitan – ME1a | Original brain | KX148188^a^ | This study |
| Iran | 93019IRA | Jackal  *(Canis aureus)* | 1993 | Cosmopolitan – CA2 | Original brain | KX148212^a^ | This study |
| Iran | 96299IRA | Wolf  *(Canis pallipes)* | 1996 | Cosmopolitan – ME1a | Original brain | KX148185^a^ | This study |
| Iran | 96320IRA | Jackal  *(Canis aureus)* | 1996 | Cosmopolitan – ME1a | Original brain | KX148187^a^ | This study |
| Israel | 93030ISR | Fox  *(Und.)* | 1993 | Cosmopolitan – ME1a | Original brain | KX148180^a^ | This study |
| Israel | 93031ISR | Fox  *(Und.)* | 1993 | Cosmopolitan – ME1a | Original brain | KX148183^a^ | This study |
| Israel | 93032ISR | Jackal  *(Canis aureus)* | 1993 | Cosmopolitan – ME1b | Original brain | KX148191^a^ | This study |
| Israel | 93033ISR | Dog  (*Canis familiaris*) | 1993 | Cosmopolitan – ME1b | Original brain | KX148192^a^ | This study |
| Israel | 96306ISR | Dog  (*Canis familiaris*) | 1996 | Cosmopolitan – ME1a | Original brain | KX148178^e^ | This study |
| Israel | 96307ISR | Dog  (*Canis familiaris*) | 1996 | Cosmopolitan – ME1a | Original brain | KX148182^e^ | This study |
| Israel | 96312ISR | Dog  (*Canis familiaris*) | 1996 | Cosmopolitan – ME1a | Original brain | KX148181^c^ | This study |
| Israel | 96314ISR | Jackal  *(Canis aureus)* | 1996 | Cosmopolitan – ME1a | Original brain | KX148179^a^ | This study |
| Israel | 96317ISR | Fox  *(Und.)* | 1996 | Cosmopolitan – ME1a | Original brain | KX148177^a^ | This study |
| Israel | 96324ISR | Cattle  (*Bos taurus*) | 1996 | Cosmopolitan – ME1a | Original brain | KX148184^a^ | This study |
| Israel | RV2324 | Dog  (*Canis familiaris*) | 1950 | Cosmopolitan – AF4 | ? | KF154998 | (16) |
| Ivory Coast | 01007CI | Dog  (*Canis familiaris*) | 2001 | Africa-2 | Original brain | KX148235^a^ | This study |
| Ivory Coast | 92037CI | Dog  (*Canis familiaris*) | 1992 | Africa-2 | Original brain | KX148232^a^ | This study |
| Ivory Coast | 92038CI | Dog  (*Canis familiaris*) | 1992 | Africa-2 | Original brain | KX148233^a^ | This study |
| Kenya | 14015ITA | Human  (*Homo sapiens*) | 2014 | Cosmopolitan – AF1a | Original brain | KX148207^c^ | This study |
| Laos | 02003LAO | Dog  (*Canis familiaris*) | 2002 | Asian – SEA3 | Original brain | KX148258^a^ | This study |
| Laos | 02004LAO | Dog  (*Canis familiaris*) | 2002 | Asian – SEA3 | Original brain | KX148256^a^ | This study |
| Laos | 02005LAO | Dog  (*Canis familiaris*) | 2002 | Asian – SEA3 | Original brain | KX148257^a^ | This study |
| Laos | 99010LAO | Dog  (*Canis familiaris*) | 1999 | Asian – SEA3 | Original brain | KX148255^a^ | This study |
| Laos | Lao2 | Dog  (*Canis familiaris*) | 2011 | Asian – SEA3 | Original brain | AB981663 | (18) |
| Laos | Lao4 | Dog  (*Canis familiaris*) | 2011 | Asian – SEA3 | Original brain | AB981664 | (18) |
| Madagascar | 04033MAD | Dog  (*Canis familiaris*) | 2004 | Cosmopolitan – AF1c | Original brain | KX148209^e^ | This study |
| Madagascar | 86046MAD | Dog  (*Canis familiaris*) | 1986 | Cosmopolitan – AF1c | Original brain | KX148211^c^ | This study |
| Madagascar | 98002MAD | Human  (*Homo sapiens*) | 1998 | Cosmopolitan – AF1c | Original brain | KX148210^e^ | This study |
| Mauritania | 93011MAU | Dog  (*Canis familiaris*) | 1993 | Africa-2 | Original brain | KX148236^a^ | This study |
| Mauritania | 93012MAU | Dog  (*Canis familiaris*) | 1993 | Africa-2 | Original brain | KX148237^a^ | This study |
| Mexico | 91010MEX | Human  (*Homo sapiens*) | 1991 | Cosmopolitan – AM2a | Mouse | KX148112^f^ | This study |
| Mexico | 91014MEX | Dog  (*Canis familiaris*) | 1991 | Cosmopolitan – AM2a | Original brain | KX148110^f^ | This study |
| Mexico | 91015MEX | Dog  (*Canis familiaris*) | 1991 | Cosmopolitan – AM2a | Original brain | KX148111^a^ | This study |
| Mexico | 91026MEX | Dog  (*Canis familiaris*) | 1991 | Cosmopolitan – AM2a | Mouse | KX148102^d^ | This study |
| Mexico | 3645DR | Human  (*Homo sapiens*) | 2009 | Bat – DR | Mouse | JQ685953 | (19) |
| Mexico | Coati_3639 | Coati  *(Nasua narica)* | 2009 | Bat – TB | Mouse | JQ685963 | (19) |
| Mexico | DRV_Mexico | Dog  (*Canis familiaris*) | ND | Cosmopolitan – AM2a | Mouse | HQ450386 | (20) |
| Mexico | MEXSK13938 | Spotted skunk  *(Spilogale putorius)* | 2007 | RAC-SK – MexSK-1 | Mouse | JQ685954 | (19) |
| Mexico | MEXSK3636 | Spotted skunk  *(Spilogale putorius)* | 2009 | Cosmopolitan – AM2b | Mouse | JQ685975 | (19) |
| Mexico | MEXSK3644 | Spotted skunk  *(Spilogale putorius)* | 2009 | RAC-SK – MexSK-1 | Mouse | JQ685929 | (19) |
| Montenegro | 86058YOU | Cow  (*Bos taurus*) | 1978 | Cosmopolitan | Mouse | KX148104^d^ | This study |
| Mozambique | 86031MOZ | Dog  (*Canis familiaris*) | 1986 | Cosmopolitan – AF1a | Mouse | KX148203^e^ | This study |
| Morocco | 04031MAR | Dog  (*Canis familiaris*) | 2004 | Cosmopolitan – AF1a | Original brain | KX148195^a^ | This study |
| Morocco | 08342MAR | Dog  (*Canis familiaris*) | 2008 | Cosmopolitan – AF1a | Original brain | KX148193^f^ | This study |
| Morocco | 89017MAR | Dog  (*Canis familiaris*) | 1989 | Cosmopolitan – AF1a | Original brain | KX148194^a^ | This study |
| Morocco | 90016MAR | Dog  (*Canis familiaris*) | 1990 | Cosmopolitan – AF1a | Original brain | KX148196^a^ | This study |
| Morocco | RV2627 | Cow  (*Bos taurus*) | 2009 | Cosmopolitan – AF1a | Original brain | KF155001 | (16) |
| Myanmar | 99009BIR | Dog  (*Canis familiaris*) | 1999 | Asian – SEA3 | Original brain | KX148247^a^ | This study |
| Myanmar | 99015BIR | Dog  (*Canis familiaris*) | 1999 | Asian – SEA3 | Original brain | KX148248^a^ | This study |
| NA | PV_RefSeq | Vaccine strain | ND | Cosmopolitan – Vac | NA | NC_001542 | (21) |
| NA | SAD_B19 | Vaccine strain | ND | Cosmopolitan – Vac | NA | M31046 | (22) |
| Namibia | 92030NAM | Dog  (*Canis familiaris*) | 1992 | Cosmopolitan – AF1b | Original brain | KX148204^a^ | This study |
| Namibia | 178J09 | Jackal  *(Und.)* | 2009 | Cosmopolitan – AF1b | ? | JX473838 | Scott *et al.,* unpublished |
| Namibia | 192J09 | Jackal  *(Und.)* | 2009 | Cosmopolitan – AF1b | ? | JX473839 | Scott *et al.,* unpublished |
| Namibia | 239K09 | Namibian kudu  (*Tragelaphus strepsiceros*) | 2009 | Cosmopolitan – AF1b | ? | JX473840 | Scott *et al.,* unpublished |
| Namibia | 240K09 | Namibian kudu  (*Tragelaphus strepsiceros*) | 2009 | Cosmopolitan – AF1b | ? | JX473841 | Scott *et al.,* unpublished |
| Nepal | 09029NEP | Buffalo  *(Und.)* | 2009 | Indian Subcontinent | Original brain | KX148245^c^ | This study |
| Nepal | 11001NEP | Cattle  (*Bos taurus*) | 2011 | Indian Subcontinent | Original brain | KX148108^d^ | This study |
| Nepal | 99001NEP | Dog  (*Canis familiaris*) | 1998 | Arctic-related – AL3 | Original brain | KX148228^a^ | This study |
| Niger | 90010NIG | Dog  (*Canis familiaris*) | 1990 | Africa-2 | Original brain | KX148231^a^ | This study |
| Niger | 90012NIG | Dog  (*Canis familiaris*) | 1990 | Africa-2 | Original brain | KX148229^a^ | This study |
| Nigeria | 86003BRE | Dog  (*Canis familiaris*) | 1986 | Cosmopolitan – AF1a | Original brain | KX148201^e^ | This study |
| Nigeria | DRV_NG11 | Dog  (*Canis familiaris*) | 2011 | Africa-2 | Original brain | KC196743 | (23) |
| Oman | 91034OMA | Camel  *(Camelus dromedarius)* | 1990 | Cosmopolitan – ME1a | Original brain | KX148169^a^ | This study |
| Oman | 91035OMA | Fox  *(Und.)* | 1990 | Cosmopolitan – ME1a | Original brain | KX148170^a^ | This study |
| Pakistan | Pk_23 | Cow  (*Bos taurus*) | 2010 | Arctic-related – AL1a | Mouse | HE802675 | Hussain *et al.,* unpublished |
| Pakistan | Pk_24 | Cow  (*Bos taurus*) | 2007 | Arctic-related – AL1a | Mouse | HE802676 | Hussain *et al.,* unpublished |
| Philippines | 04030PHI | Dog  (*Canis familiaris*) | 2004 | Asian – SEA4 | Mouse | KX148260^e^ | This study |
| Philippines | 94272PHI | Dog  (*Canis familiaris*) | 1994 | Asian – SEA4 | Original brain | KX148259^a^ | This study |
| Philippines | 94275PHI | Dog  (*Canis familiaris*) | 1994 | Asian – SEA4 | Original brain | KX148263^a^ | This study |
| Philippines | 94278PHI | Dog  (*Canis familiaris*) | 1994 | Asian – SEA4 | Original brain | KX148262^f^ | This study |
| Philippines | 94281PHI | Dog  (*Canis familiaris*) | 1994 | Asian – SEA4 | Original brain | KX148261^a^ | This study |
| Poland | 96026POL | Raccoon dog  *(Nyctereutes procyonoides)* | 1986 | Cosmopolitan – NEE | Original brain | KX148150^a^ | This study |
| Poland | 96042POL | Red fox  (*Vulpes vulpes*) | 1996 | Cosmopolitan – NEE | Original brain | KX148151^a^ | This study |
| Poland | 96045POL | Red fox  (*Vulpes vulpes*) | 1993 | Cosmopolitan – CE | Original brain | KX148119^a^ | This study |
| Poland | 96056POL | Red fox  (*Vulpes vulpes*) | 1993 | Cosmopolitan – CE | Original brain | KX148121^a^ | This study |
| Poland | 96077POL | Raccoon dog  *(Nyctereutes procyonoides)* | 1994 | Cosmopolitan – CE | Original brain | KX148118^a^ | This study |
| Poland | 96097POL | Raccoon dog  *(Nyctereutes procyonoides)* | 1996 | Cosmopolitan – NEE | Original brain | KX148153^a^ | This study |
| Poland | 96104POL | Red fox  (*Vulpes vulpes*) | 1995 | Cosmopolitan – CE | Original brain | KX148114^a^ | This study |
| Poland | 96135POL | Red fox  (*Vulpes vulpes*) | 1992 | Cosmopolitan – EE | Original brain | KX148141^a^ | This study |
| Poland | 96140POL | Raccoon dog  *(Nyctereutes procyonoides)* | 1993 | Cosmopolitan – CE | Original brain | KX148120^a^ | This study |
| Poland | 96142POL | Raccoon dog  *(Nyctereutes procyonoides)* | 1996 | Cosmopolitan – NEE | Original brain | KX148152^a^ | This study |
| Poland | 96177POL | Red fox  (*Vulpes vulpes*) | 1994 | Cosmopolitan – EE | Original brain | KX148142^a^ | This study |
| Poland | 96250POL | Red fox  (*Vulpes vulpes*) | 1996 | Cosmopolitan – CE | Original brain | KX148115^a^ | This study |
| Poland | 96256POL | Red fox  (*Vulpes vulpes*) | 1996 | Cosmopolitan – CE | Original brain | KX148116^a^ | This study |
| Poland | 97046POL | Red fox  (*Vulpes vulpes*) | 1997 | Cosmopolitan – CE | Original brain | KX148117^a^ | This study |
| Poland | 97078POL | Raccoon dog  *(Nyctereutes procyonoides)* | 1997 | Cosmopolitan – NEE | Original brain | KX148154^a^ | IP |
| Russia | 1350KRA | Dog  (*Canis familiaris*) | 2008 | Cosmopolitan – CA1 | Original brain | JQ944705 | Chupin *et al.,* unpublished |
| Russia | 1352KRA | Dog  (*Canis familiaris*) | 2008 | Cosmopolitan – CA2 | Original brain | JQ944706 | Chupin *et al.,* unpublished |
| Russia | 1410KOM | Deer  *(Und.)* | 2008 | Arctic-related – A | Original brain | JQ944707 | Chupin *et al.,* unpublished |
| Russia | 1564NNO | Red fox  (*Vulpes vulpes*) | 2008 | Cosmopolitan – CA3 | Original brain | JQ944708 | Chupin *et al.,* unpublished |
| Russia | 184VNO | Raccoon dog  *(Nyctereutes procyonoides)* | 2009 | Cosmopolitan – NEE | Original brain | JQ944704 | Chupin *et al.,* unpublished |
| Russia | 8052f | Red fox  (*Vulpes vulpes*) | 2011 | Cosmopolitan – CA1 | Mouse | KC595280 | (24) |
| Russia | 8053c | Cat  *(Felis catus)* | 2011 | Cosmopolitan – CA1 | Mouse | KC595281 | (24) |
| Russia | 8054f | Red fox  (*Vulpes vulpes*) | 2011 | Cosmopolitan – CA1 | Mouse | KC595282 | (24) |
| Russia | 8057f | Red fox  (*Vulpes vulpes*) | 2011 | Cosmopolitan – CA1 | Mouse | KC595283 | (24) |
| Rwanda | 94289RWA | Dog  (*Canis familiaris*) | 1994 | Cosmopolitan – AF1b | Original brain | KX148205^a^ | This study |
| Saudi Arabia | 04034ARS | Fox  *(Und.)* | 2004 | Cosmopolitan – ME1a | Original brain | KX148175^a^ | This study |
| Saudi Arabia | 87006ARS | Fox  *(Und.)* | 1987 | Cosmopolitan – ME1a | Original brain | KX148168^a^ | This study |
| Saudi Arabia | 87007ARS | Horse  (*Equus ferus*) | 1987 | Cosmopolitan – ME1a | Original brain | KX148172^a^ | This study |
| Saudi Arabia | 97139ARS | Red fox  *(Vulpes vulpes)* | 1997 | Cosmopolitan – ME1a | Original brain | KX148173^a^ | This study |
| Saudi Arabia | 98001ARS | Fox  *(Und.)* | 1998 | Cosmopolitan – ME1a | Original brain | KX148174^a^ | This study |
| Saudi Arabia | 99004ARS | Red fox  *(Vulpes vulpes)* | 1998 | Cosmopolitan – ME1a | Original brain | KX148175^a^ | This study |
| Senegal | 93003SEN | Dog  (*Canis familiaris*) | 1991 | Africa-2 | Original brain | KX148238^c^ | This study |
| Senegal | 93005SEN | Dog  (*Canis familiaris*) | 1992 | Africa-2 | Original brain | KX148239^c^ | This study |
| Serbia | 86109YOU | Red fox  (*Vulpes vulpes*) | 1986 | Cosmopolitan – EE | Original brain | KX148140^a^ | This study |
| Serbia | 86133SER | Red fox  (*Vulpes vulpes*) | 1972 | Cosmopolitan | Mouse | KX148161^e^ | This study |
| Slovenia | 94086SLN | Red fox  (*Vulpes vulpes*) | 1994 | Cosmopolitan – WE | Original brain | KX148131^a^ | This study |
| Slovenia | 94091SLN | Red fox  (*Vulpes vulpes*) | 1994 | Cosmopolitan – WE | Original brain | KX148129^a^ | This study |
| Slovenia | 94094SLN | Red fox  (*Vulpes vulpes*) | 1994 | Cosmopolitan – WE | Original brain | KX148132^a^ | This study |
| Slovenia | 94096SLN | Red fox  (*Vulpes vulpes*) | 1994 | Cosmopolitan – WE | Original brain | KX148133^a^ | This study |
| Slovenia | 94099SLN | Red fox  (*Vulpes vulpes*) | 1994 | Cosmopolitan – EE | Original brain | KX148144^a^ | This study |
| Somalia | 93006SOM | Jackal  *(Canis mesomelas)* | 1993 | Cosmopolitan – AF1a | Original brain | KX148199^e^ | This study |
| Somalia | 93128SOM | Dog  (*Canis familiaris*) | 1993 | Cosmopolitan – AF1a | Original brain | KX148198^a^ | This study |
| South Africa | 14018AFS | Feline  *(Und.)* | 2000 | Africa-3 | Original brain | KX148223^e^ | This study |
| South Africa | 15001AFS | Yellow mongoose *(Cynictis penicillata)* | 2013 | Africa-3 | Original brain | KX148220^b^ | This study |
| South Africa | 15002AFS | Mongoose  *(Und.)* | 2014 | Africa-3 | Original brain | KX148221^b^ | This study |
| South Africa | 15003AFS | Ground squirrel  *(Xerus Inauris)* | 2014 | Africa-3 | Original brain | KX148222^b^ | This study |
| South Africa | 87021AFS | Human  (*Homo sapiens*) | 1981 | Cosmopolitan – AF1b | Mouse | KX148103^d^ | This study |
| South Korea | 08F40 | Cattle  (*Bos taurus*) | 2008 | Arctic-related – AL2 | Original brain | KC171643 | (25) |
| South Korea | BD0406CC | Raccoon dog  *(Nyctereutes procyonoides)* | 2004 | Arctic-related – AL2 | Original brain | KC171644 | (25) |
| South Korea | BV9901PJ | Raccoon dog  *(Nyctereutes procyonoides)* | 1999 | Arctic-related – AL2 | Original brain | KC171645 | (25) |
| Sri Lanka | H_1413_09 | Golden palm civet  *(Paradoxurus zeylonensis)* | 2009 | Indian Subcontinent | Original brain | AB635373 | (26) |
| Sri Lanka | RV2417 | Dog  (*Canis familiaris*) | 2008 | Indian Subcontinent | Original brain | KF154999 | (16) |
| Taiwan | R2012_26 | Ferret badger  *(Melogale moschata)* | 2012 | Asian – SEA5 | Original brain | KF620487 | (27) |
| Taiwan | R2012_88 | Ferret badger  *(Melogale moschata)* | 2012 | Asian – SEA5 | Original brain | KF620488 | (27) |
| Taiwan | R2013_01 | Ferret badger  *(Melogale moschata)* | 2013 | Asian – SEA5 | Original brain | KF620489 | (27) |
| Tanzania | 96013TAN | Dog  (*Canis familiaris*) | 1996 | Cosmopolitan – AF1b | Original brain | KX148206^a^ | This study |
| Tanzania | RV2772 | Dog  (*Canis familiaris*) | 2009 | Cosmopolitan – AF1b | Original brain | KF155002 | (16) |
| Thailand | 8764THA | Human  *(Homo sapiens)* | 1983 | Asian – SEA3 | Mouse | EU293111 | (28) |
| Thailand | QS_05 | Dog  (*Canis familiaris*) | ND | Asian – SEA3 | Mouse | JN786877 | (29) |
| Turkey | 93100TUR | Dog  (*Canis familiaris*) | 1993 | Cosmopolitan – ME2 | Original brain | KX148164^e^ | This study |
| Turkey | 94001TUR | Dog  (*Canis familiaris*) | 1993 | Cosmopolitan – ME2 | Original brain | KX148166^b^ | This study |
| Turkey | 94002TUR | Dog  (*Canis familiaris*) | 1993 | Cosmopolitan – ME2 | Original brain | KX148162^a^ | This study |
| Turkey | 94005TUR | Dog  (*Canis familiaris*) | 1993 | Cosmopolitan – ME2 | Original brain | KX148167^e^ | This study |
| Turkey | 94009TUR | Dog  (*Canis familiaris*) | 1993 | Cosmopolitan – ME2 | Original brain | KX148165^b^ | This study |
| Turkey | 94016TUR | Dog  (*Canis familiaris*) | 1993 | Cosmopolitan – ME2 | Original brain | KX148163^e^ | This study |
| United Arab Emirates | 91024EAU | Fox  *(Und.)* | 1991 | Cosmopolitan – ME1a | Mouse | KX148171^c^ | This study |
| USA | 91001USA | Skunk  *(Und.)* | 1982 | Cosmopolitan – AM1 | Mouse | KX148213^e^ | This study |
| USA | 91004USA | Striped skunk  (*Mephitis mephitis)* | 1991 | Arctic-related – A | Original brain | KX148224^e^ | This study |
| USA | 91005USA | Red fox  *(Vulpes vulpes)* | 1990 | Arctic-related – A | Original brain | KX148106^d^ | This study |
| USA | 1088 | Woodchuck  *(Marmota monax)* | 2008 | Cosmopolitan – AM1 | Mouse | AB645847 | (30) |
| USA | 2401 | Gray Fox  *(Urocyon cinereoargenteus)* | 2009 | Bat – EF-W1 | Original brain | JQ685934 | (19) |
| USA | A02_2971 | Bat  (*Parastrellus Hesperus*) | 2002 | Bat – PH | N, P, M, G - original brain; L and termini - mouse | JQ685952 | (19) |
| USA | A02_2972 | Bat  (*Parastrellus hesperus*) | 2002 | Bat – PH | N, P, M, G - original brain; L and termini - mouse | JQ685965 | (19) |
| USA | A093500 | Bat  (*Eptesicus fuscus*) | 2009 | Bat – EF-W1 | N, P, M, G - original brain; L and termini - mouse | JQ685898 | (19) |
| USA | A093504 | Bat  (*Eptesicus fuscus*) | 2009 | Bat – EF-W1 | N, P, M, G - original brain; L and termini - mouse | JQ685950 | (19) |
| USA | A10_0511 | Gray Fox  *(Urocyon cinereoargenteus)* | 2009 | Cosmopolitan – AM2b | Original brain | JQ685943 | (19) |
| USA | A10_0512 | Striped skunk  *(Mephitis mephitis)* | 2009 | RAC-SK – SCSK | Original brain | JQ685968 | (19) |
| USA | A10_0514 | Striped skunk  *(Mephitis mephitis)* | 2009 | RAC-SK – SCSK | Original brain | JQ685938 | (19) |
| USA | A10_0515 | Gray Fox  *(Urocyon cinereoargenteus)* | 2009 | Cosmopolitan – AM2b | Original brain | JQ685899 | (19) |
| USA | A11_1043 | Coyote  *(Canis latrans)* | 2011 | Bat – EF-W2 | Original brain | JQ685973 | (19) |
| USA | A11_5300 | Human  (*Homo sapiens*) | 2011 | Cosmopolitan – AM3a | Original brain | KC737850 | McGuone *et al.,* unpublished |
| USA | AZ10_140 | Bat  (*Eptesicus fuscus*) | 2010 | Bat – EF-W1 | N, P, M, G - original brain; L and termini - mouse | JQ685961 | (19) |
| USA | AZ3003 | Bat  (*Antrozous pallidus*) | 2009 | Bat – AZ | N, P, M, G - original brain; L and termini - mouse | JQ685971 | (19) |
| USA | AZ4490 | Bat  (*Myotis yumanensis*) | 2005 | Bat – MYu | N, P, M, G - original brain; L and termini - mouse | JQ685955 | (19) |
| USA | AZBAT_6509 | Bat  (*Eptesicus fuscus*) | 1981 | Bat – EF-W2 | Mouse | JQ685942 | (19) |
| USA | AZBAT_6763 | Bat  (*Eptesicus fuscu*s) | 1985 | Bat – EF-W1 | Mouse | JQ685913 | (19) |
| USA | AZBAT_7453 | Bat  (*Eptesicus fuscus*) | 1975 | Bat – EF-W1 | Mouse | JQ685956 | (19) |
| USA | CA04148 | Bat  (*Eptesicus fuscus*) | 2004 | Bat – EF-W2 | N, P, M, G - original brain; L and termini - mouse | JQ685903 | (19) |
| USA | CA100 | Bat  (*Eptesicus fuscus*) | 2005 | Bat – EF-W1 | N, P, M, G - original brain; L and termini - mouse | JQ685909 | (19) |
| USA | CA982 | Striped skunk  *(Mephitis mephitis)* | 1994 | Cosmopolitan – AM4 | Original brain | JQ685894 | (19) |
| USA | CASK2 | Striped skunk  *(Mephitis mephitis)* | 1974 | Cosmopolitan – AM4 | Original brain | JQ685970 | (19) |
| USA | CO_Coyot_2 | Coyote  *(Canis latrans)* | 2010 | Bat – EF-W1 | Original brain | JQ685917 | (19) |
| USA | EF | Bat  (*Eptesicus fuscu*s) | 1984 | Bat – EF | mouse | JQ685920 | (19) |
| USA | FL1010 | Bat  (*Lasiurus intermedius*) | 2002 | Bat – LI | N, P, M, G - original brain; L and termini - mouse | JQ685916 | (19) |
| USA | FL1078 | Bat  (*Myotis autroriparius*) | 2001 | Bat – MYsp | N, P, M, G - original brain; L and termini - mouse | JQ685921 | (19) |
| USA | FL385 | Bat  (*Tadarida brasiliensis*) | 2003 | Bat – TB | N, P, M, G - original brain; L and termini - mouse | JQ685905 | (19) |
| USA | FL769 | Bat  (*Lasiurus seminolus*) | 2003 | Bat – LS | N, P, M, G - original brain; L and termini - mouse | JQ685900 | (19) |
| USA | NC1234 | Striped skunk  *(Mephitis mephitis)* | ND | Cosmopolitan – AM1 | Original brain | JQ685967 | (19) |
| USA | NC839 | Striped skunk  *(Mephitis mephitis)* | 1984 | Cosmopolitan – AM1 | Original brain | JQ685944 | (19) |
| USA | NJ2262 | Bat  (*Lasiurus borealis*) | 2005 | Bat – LB | N, P, M, G - original brain; L and termini - mouse | JQ685919 | (19) |
| USA | OR05455 | Gray Fox  *(Urocyon cinereoargenteus)* | 2010 | Bat – EF-W2 | Original brain | JQ685948 | (19) |
| USA | OR05506 | Gray Fox  *(Urocyon cinereoargenteus)* | 2010 | Bat – MYsp | Original brain | JQ685918 | (19) |
| USA | OR8767 | Gray Fox  *(Urocyon cinereoargenteus)* | 2005 | Bat – MYsp | Original brain | JQ685957 | (19) |
| USA | RAC | Raccoon  *(Procyon lotor)* | 2003 | RAC-SK – RAC | Mouse | JQ685901 | (19) |
| USA | SHBRV_18 | Human  (*Homo sapiens*) | 1983 | Bat – PS | Mouse | AY705373 | (31) |
| USA | SM1545 | Striped skunk  *(Mephitis mephitis)* | 2005 | Bat – EF-W1 | Original brain | JQ685941 | (19) |
| USA | SM3844 | Bat  (*Eptesicus fuscus*) | 1995 | Bat – EF-W1 | N, P, M, G - original brain; L and termini - mouse | JQ685974 | (19) |
| USA | SM3849 | Bat  (*Eptesicus fuscus*) | 1996 | Bat – EF-W1 | N, P, M, G - original brain; L and termini - mouse | JQ685907 | (19) |
| USA | SM4862 | Bat  (*Eptesicus fuscus*) | 1999 | Bat – EF-W1 | N, P, M, G - original brain; L and termini - mouse | JQ685946 | (19) |
| USA | SM4871 | Bat  (*Eptesicus fuscus*) | 1999 | Bat – EF-W1 | N, P, M, G - original brain; L and termini - mouse | JQ685923 | (19) |
| USA | SM4872 | Striped skunk  *(Mephitis mephitis)* | 2001 | Bat – EF-W1 | Original brain | JQ685960 | (19) |
| USA | SM5076 | Striped skunk  *(Mephitis mephitis)* | 2001 | Bat – EF-W1 | Original brain | JQ685932 | (19) |
| USA | SM5077 | Striped skunk  *(Mephitis mephitis)* | 2001 | Bat – EF-W1 | Original brain | JQ685911 | (19) |
| USA | SM5079 | Striped skunk  *(Mephitis mephitis)* | 2001 | Bat – EF-W1 | Original brain | JQ685893 | (19) |
| USA | SM5081 | Striped skunk  *(Mephitis mephitis)* | 2001 | Bat – EF-W1 | Original brain | JQ685904 | (19) |
| USA | SM5100 | Striped skunk  *(Mephitis mephitis)* | 2001 | Bat – EF-W1 | Original brain | JQ685940 | (19) |
| USA | SM5101 | Striped skunk  *(Mephitis mephitis)* | 2001 | Bat – EF-W1 | Original brain | JQ685958 | (19) |
| USA | SM5440 | Striped skunk  *(Mephitis mephitis)* | 2001 | Bat – EF-W1 | Original brain | JQ685969 | (19) |
| USA | SM5441 | Striped skunk  *(Mephitis mephitis)* | 2001 | Bat – EF-W1 | Original brain | JQ685962 | (19) |
| USA | SM5442 | Bat  (*Eptesicus fuscus*) | 2001 | Bat – EF-W1 | N, P, M, G - original brain; L and termini - mouse | JQ685897 | (19) |
| USA | SM5451 | Striped skunk  *(Mephitis mephitis)* | 2001 | Bat – EF-W1 | Original brain | JQ685959 | (19) |
| USA | SM5470 | Striped skunk  *(Mephitis mephitis)* | 2001 | Bat – EF-W1 | Original brain | JQ685966 | (19) |
| USA | SM5596 | Striped skunk  *(Mephitis mephitis)* | 2004 | Bat – EF-W1 | Original brain | JQ685964 | (19) |
| USA | SM5950 | Gray fox  *(Urocyon cinereoargenteus)* | 2004 | Bat – EF-W1 | Original brain | JQ685933 | (19) |
| USA | SM6709 | Cat  *(Felis catus)* | 2005 | Bat – EF-W1 | Original brain | JQ685945 | (19) |
| USA | TN186 | Bat  (*Perimyotis subflavus*) | 2005 | Bat – PS | N, P, M, G - original brain; L and termini - mouse | JQ685922 | (19) |
| USA | TN209 | Bat  (*Lasiurus boreali*s) | 2005 | Bat – LB | N, P, M, G - original brain; L and termini - mouse | JQ685902 | (19) |
| USA | TN310 | Bat  (*Lasiurus cinereus*) | 2004 | Bat – LC | N, P, M, G - original brain; L and termini - mouse | JQ685947 | (19) |
| USA | TX4904 | Bat  (*Lasiurus intermedius*) | 2002 | Bat – LI | N, P, M, G - original brain; L and termini - mouse | JQ685915 | (19) |
| USA | TX5960 | Bat  (*Lasiurus xanthinus*) | 2002 | Bat – LX | N, P, M, G - original brain; L and termini - mouse | JQ685910 | (19) |
| USA | WA0173 | Bat  (*Eptesicus fuscu*s) | 2000 | Bat – EF-W2 | N, P, M, G - original brain; L and termini - mouse | JQ685931 | (19) |
| USA | WA1185 | Bat  (*Lasionycteris noctivagans*) | 2003 | Bat – LN | N, P, M, G - original brain; L and termini - mouse | JQ685895 | (19) |
| USA | WAEF03 | Bat  (*Eptesicus fuscus*) | 2004 | Bat – EF | N, P, M, G - original brain; L and termini - mouse | JQ685925 | (19) |
| Vietnam | 01016VNM | Dog  (*Canis familiaris*) | 2001 | Asian – SEA3 | Mouse | KX148261^e^ | This study |

The full-length genome sequences generated in the present study have been deposited to GenBank under accession numbers KX148100-KX148269 and have been obtained as follow:

^a^ dsDNA are obtained using the 6 PCR fragments strategy and fragmented by fragmentase; libraries were prepared using NEBNext Ultra DNA Library Prep kit and sequenced using an 100 nucleotides single-end strategy on the NextSeq500 platform.

^b^ dsDNA are obtained using the 6 PCR fragments strategy; libraries were constructed using Nextera XT kit and sequenced using a 2 x 150 nucleotides paired-end strategy on the NextSeq500 platform.

^c^ dsDNA are obtained using the 6 PCR fragments strategy and fragmented by ultrasounds; libraries were prepared using NEXTflex PCR-Free DNA-Seq kit and sequenced using an 150 nucleotides single-end strategy on the HiSeq2500 platform.

^d^ dsDNA are obtained using the whole-transcription amplification (WTA) protocol and fragmented by ultrasounds; libraries were prepared using TruSeq protocol and sequenced using an 100 nucleotides single-end strategy on the HiSeq2000 platform.

^e^ dsDNA are obtained using the 6 PCR fragments strategy and fragmented by ultrasounds; libraries were prepared using NEXTflex PCR-Free DNA-Seq kit and sequenced using a 2 x 300 nucleotides paired-end strategy on the MiSeq platform.

^f^ dsDNA are obtained using the 6 PCR fragments strategy and fragmented by ultrasounds; libraries were prepared using NEXTflex PCR-Free DNA-Seq kit and sequenced using an 100 nucleotides single-end strategy on the HiSeq2500 platform.

^g^ sequence is obtained using a shotgun strategy as previously described (28).

*Und.*: undetermined species

**REFERENCES**

1. Mochizuki N, Kobayashi Y, Sato G, Hirano S, Itou T, Ito FH, et al. Determination and molecular analysis of the complete genome sequence of two wild-type rabies viruses isolated from a haematophagous bat and a frugivorous bat in Brazil. J Vet Med Sci. 2011;73(6):759-66.

2. Mochizuki N, Kobayashi Y, Sato G, Itou T, Gomes AA, Ito FH, et al. Complete genome analysis of a rabies virus isolate from Brazilian wild fox. Archives of virology. 2009;154(9):1475-88.

3. Szanto AG, Nadin-Davis SA, White BN. Complete genome sequence of a raccoon rabies virus isolate. Virus research. 2008;136(1-2):130-9.

4. Tricou V, Berthet N, Nakoune E, Kazanji M. Complete genome sequence of a rabies virus isolated from a human in central african republic. Genome Announc. 2014;2(3).

5. Zhao J, Liu Y, Zhang S, Zhang F, Wang Y, Mi L, et al. Molecular characterization of three ferret badger (Melogale moschata) rabies virus isolates from Jiangxi province, China. Archives of virology. 2014;159(8):2059-67.

6. Zhao J, Zhang S, Liu Y, Zhang F, Hu R. Complete Genome Sequence of a Rabies Virus Isolate from a Ferret Badger (Melogale moschata) in Jiangxi, China. Genome Announc. 2013;1(3).

7. He CQ, Meng SL, Yan HY, Ding NZ, He HB, Yan JX, et al. Isolation and identification of a novel rabies virus lineage in China with natural recombinant nucleoprotein gene. PloS one. 2012;7(12):e49992.

8. Zhang J, Zhang HL, Tao XY, Li H, Tang Q, Jiang XY, et al. The full-length genome analysis of a street rabies virus strain isolated in Yunnan province of China. Virologica Sinica. 2012;27(3):204-13.

9. Yu F, Zhang G, Xiao S, Fang L, Xu G, Yan J, et al. Complete genome sequence of a street rabies virus isolated from a rabid dog in China. Journal of virology. 2012;86(19):10890-1.

10. Luo Y, Zhang Y, Liu X, Yang Y, Yang X, Zhang D, et al. Complete genome sequence of a highly virulent rabies virus isolated from a rabid pig in south China. Journal of virology. 2012;86(22):12454-5.

11. Tang HB, He XX, Zhong YZ, Liao SH, Zhong TZ, Xie LJ, et al. Complete genome sequence of a rabies virus isolate from cattle in guangxi, southern china. Genome Announc. 2013;1(1).

12. Ming P, Du J, Tang Q, Yan J, Nadin-Davis SA, Li H, et al. Molecular characterization of the complete genome of a street rabies virus isolated in China. Virus research. 2009;143(1):6-14.

13. Zhu H, Chen X, Shao X, Ba H, Wang F, Wang H, et al. Characterization of a virulent dog-originated rabies virus affecting more than twenty fallow deer (Dama dama) in Inner Mongolia, China. Infection, genetics and evolution : journal of molecular epidemiology and evolutionary genetics in infectious diseases. 2015;31:127-34.

14. Xie T, Yu H, Wu J, Ming P, Huang S, Shen Z, et al. Molecular characterization of the complete genome of a street rabies virus WH11 isolated from donkey in China. Virus genes. 2012;45(3):452-62.

15. Zhao J, Wang S, Zhang S, Liu Y, Zhang J, Zhang F, et al. Molecular characterization of a rabies virus isolate from a rabid dog in Hanzhong District, Shaanxi Province, China. Archives of virology. 2014;159(6):1481-6.

16. Marston DA, McElhinney LM, Ellis RJ, Horton DL, Wise EL, Leech SL, et al. Next generation sequencing of viral RNA genomes. BMC Genomics. 2013;14:444.

17. Marston DA, Wise EL, Ellis RJ, McElhinney LM, Banyard AC, Johnson N, et al. Complete genomic sequence of rabies virus from an ethiopian wolf. Genome Announc. 2015;3(2).

18. Ahmed K, Phommachanh P, Vorachith P, Matsumoto T, Lamaningao P, Mori D, et al. Molecular epidemiology of rabies viruses circulating in two rabies endemic provinces of Laos, 2011-2012: regional diversity in Southeast Asia. PLoS neglected tropical diseases. 2015;9(3):e0003645.

19. Kuzmin IV, Shi M, Orciari LA, Yager PA, Velasco-Villa A, Kuzmina NA, et al. Molecular inferences suggest multiple host shifts of rabies viruses from bats to mesocarnivores in Arizona during 2001-2009. PLoS pathogens. 2012;8(6):e1002786.

20. Zhang G, Fu ZF. Complete genome sequence of a street rabies virus from Mexico. Journal of virology. 2012;86(19):10892-3.

21. Tordo N, Poch O, Ermine A, Keith G, Rougeon F. Completion of the rabies virus genome sequence determination: highly conserved domains among the L (polymerase) proteins of unsegmented negative-strand RNA viruses. Virology. 1988;165(2):565-76.

22. Conzelmann KK, Cox JH, Schneider LG, Thiel HJ. Molecular cloning and complete nucleotide sequence of the attenuated rabies virus SAD B19. Virology. 1990;175(2):485-99.

23. Zhou M, Zhou Z, Kia GS, Gnanadurai CW, Leyson CM, Umoh JU, et al. Complete genome sequence of a street rabies virus isolated from a dog in Nigeria. Genome Announc. 2013;1(1).

24. Poleshchuk EM, Deviatkin AA, Dedkov VG, Sidorov GN, Ochkasova JV, Hodjakova IA, et al. Complete genome sequences of four virulent rabies virus strains isolated from rabid animals in Russia. Genome Announc. 2013;1(3).

25. Oem JK, Kim SH, Kim YH, Lee MH, Lee KK. Complete genome sequences of three rabies viruses isolated from rabid raccoon dogs and a cow in Korea. Virus genes. 2013;47(3):563-8.

26. Matsumoto T, Ahmed K, Wimalaratne O, Nanayakkara S, Perera D, Karunanayake D, et al. Novel sylvatic rabies virus variant in endangered golden palm civet, Sri Lanka. Emerging infectious diseases. 2011;17(12):2346-9.

27. Chiou HY, Hsieh CH, Jeng CR, Chan FT, Wang HY, Pang VF. Molecular characterization of cryptically circulating rabies virus from ferret badgers, Taiwan. Emerging infectious diseases. 2014;20(5):790-8.

28. Delmas O, Holmes EC, Talbi C, Larrous F, Dacheux L, Bouchier C, et al. Genomic diversity and evolution of the lyssaviruses. PloS one. 2008;3(4):e2057.

29. Virojanapirom P, Khawplod P, Sawangvaree A, Wacharapluesadee S, Hemachudha T, Yamada K, et al. Molecular analysis of the mutational effects of Thai street rabies virus with increased virulence in mice after passages in the BHK cell line. Archives of virology. 2012;157(11):2201-5.

30. Yamada K, Park CH, Noguchi K, Kojima D, Kubo T, Komiya N, et al. Serial passage of a street rabies virus in mouse neuroblastoma cells resulted in attenuation: potential role of the additional N-glycosylation of a viral glycoprotein in the reduced pathogenicity of street rabies virus. Virus research. 2012;165(1):34-45.

31. Faber M, Pulmanausahakul R, Nagao K, Prosniak M, Rice AB, Koprowski H, et al. Identification of viral genomic elements responsible for rabies virus neuroinvasiveness. Proceedings of the National Academy of Sciences of the United States of America. 2004;101(46):16328-32.
