# Supplementary material for: Large-Scale Phylogenomic Analysis Reveals the Complex Evolutionary History of Rabies Virus in Multiple Carnivore Hosts
Source: PLoS Pathog. 2016 Dec 15;12(12):e1006041. doi: 10.1371/journal.ppat.1006041 (PMC5158080; doi:10.1371/journal.ppat.1006041)
Supplement: S2 Table — (DOCX) [file ppat.1006041.s008.docx]

**Table S2: Bootstrap values of nodes corresponding to all clades, subclades and lineages of the dog-related group defined in Fig 1.**

| **CLADE** | Bootstrap  values^a^ | Bootstrap values  of the node above^b^ |
| --- | --- | --- |
| **AFRICA 2** | **100** | **100** |
|  |  |  |
| **AFRICA 3** | **100** | **95** |
|  |  |  |
| **ARCTIC RELATED** | **100** | **100** |
| Arctic (A) | 100 | 100 |
| Arctic-like 1 (AL1) | 100 | 100 |
| *Arctic like 1a (AL1a)* | *100* | *100* |
| *Arctic like 1b (AL1b)* | *100* | *100* |
| Arctic like 2 (AL2) | 100 | 100 |
| Arctic like 3 (AL3) | 100 | 100 |
|  |  |  |
| **ASIAN** | **100** | **81** |
| Southeast Asia 1 (SEA1) | 100 | 100 |
| *Southeast Asia 1a (SEA1a)* | *100* | *100* |
| *Southeast Asia 1b (SEA1b)* | *100* | *100* |
| Southeast Asia 2 (SEA2) | 100 | 48 |
| *Southeast Asia 2a* (*SEA2a)* | *100* | *100* |
| *Southeast Asia 2b* (*SEA2b)* | *100* | *100* |
| Southeast Asia 3 (SEA3) | 100 | 99 |
| Southeast Asia 4 (SEA4) | 100 | 48 |
| Southeast Asia 5 (SEA5) | 100 | 100 |
|  |  |  |
| **COSMOPOLITAN** | **100** | **95** |
| Africa 1 (AF1) | 100 | 100 |
| *Africa 1a (AF1a)* | *100* | *100* |
| *Africa 1b (AF1b)* | *100* | *71* |
| *Africa 1c (AF1c)* | *100* | *71* |
| Africa 4 (AF4) | 100 | 100 |
| America 1 (AM1) | 100 | 100 |
| America 2 (AM2) | 100 | 47 |
| *America 2a (AM2a)* | *100* | *100* |
| *America 2b (AM2b)* | *99* | *100* |
| America 3 (AM3) | 100 | 31 |
| *America 3a (AM3a)* | *100* | *100* |
| *America 3b (AM3b)* | *100* | *100* |
| America 4 (AM4) | 100 | 89 |
| Central Asia 1 (CA1) | 100 | 90 |
| Central Asia 2 (CA2) | 100 | 58 |
| Central Asia 3 (CA3) | 100 | 90 |
| Europe | 100 |  |
| *Central Europe (CE)* | *100* | *100* |
| *East Europe (EE)* | *100* | *100* |
| *West Europe (WE)* | *100* | *100* |
| *North East Europe (NEE)* | *100* | *98* |
| Middle East 1 (ME1) | 100 | 71 |
| *Middle East 1a (ME1a)* | *100* | *100* |
| *Middle East 1b (ME1b)* | *100* | *100* |
| Middle East 2 (ME2) | 100 | 100 |
| Vaccine strain (Vac.) | 100 | 100 |
|  |  |  |
| **INDIAN SUB.** | **100** | **100** |

^a^ Bootstrap value of node that defined each clade, subclade or lineage

^b^ Bootstrap value of previous node of each clade, subclade or lineage
