# Supplementary material for: Large-Scale Phylogenomic Analysis Reveals the Complex Evolutionary History of Rabies Virus in Multiple Carnivore Hosts
Source: PLoS Pathog. 2016 Dec 15;12(12):e1006041. doi: 10.1371/journal.ppat.1006041 (PMC5158080; doi:10.1371/journal.ppat.1006041)
Supplement: S3 Table — (DOCX) [file ppat.1006041.s009.docx]

**Table S3: Evolutionary characteristics of dog-related RABV group clades, subclades and lineages.**

| **RABV clade /**  **subclade / lineage** | **TMRCA (95% HPD)** | **Posterior** | **TMRCA of the node above (95% HPD)** | **Posterior** |
| --- | --- | --- | --- | --- |
| **AFRICA 2** | **1802 (1750-1852)** | **1** | **1508 (1424-1596)** | **1** |
|  |  |  |  |  |
| **AFRICA 3** | **1756 (1710-1815)** | **1** | **1615 (1549-1684)** | **1** |
|  |  |  |  |  |
| **ARCTIC RELATED** | **1770 (1725-1815)** | **1** | **1598 (1520-1661)** | **1** |
| Arctic (A) | 1942 (1929-1954) | 1 | 1815 (1777-1853) | 1 |
| Arctic-like 1 (AL1) | 1918 (1901-1935) | 1 | 1881 (1856-1906) | 1 |
| *Arctic-like 1a (AL1a)* | *1940 (1927-1953)* | *1* | *1918 (1901-1935)* | *1* |
| *Arctic-like 1b (AL1b)* | *1936 (1919-1953)* | *1* | *1918 (1901-1935)* | *1* |
| Arctic-like 2 (AL2) | 1886 (1852-1921) | 1 | 1770 (1725-1815) | 1 |
| Arctic-like 3 (AL3) | 1881 (1856-1906) |  | 1770 (1725-1815) | 1 |
|  |  |  |  |  |
| **ASIAN** | **1604 (1535-1677)** | **1** | **1459 (1355-1563)** | **0.77** |
| Southeast Asia 1 (SEA1) | 1735 (1677-1795) | 1 | 1604 (1535-1677) | 1 |
| *Southeast Asia 1a (SEA1a)* | *1973 (1967-1978)* | *1* | *1735 (1677-1795)* | *1* |
| *Southeast Asia 1b (SEA1b)* | *1830 (1789-1873)* | *1* | *1735 (1677-1795)* | *1* |
| Southeast Asia 2 (SEA2) | 1864 (1830-1898) | 1 | 1725 (1670-1782) | 0.86 |
| *Southeast Asia 2a* (*SEA2a)* | *1956 (1945-1967)* | *1* | *1864 (1830-1898)* | *1* |
| *Southeast Asia 2b* (*SEA2b)* | *1951 (1937-1968)* | *1* | *1864 (1830-1898)* | *1* |
| Southeast Asia 3 (SEA3) | 1898 (1876-1920) | 1 | 1640 (1574-1708) | 1 |
| Southeast Asia 4 (SEA4) | 1925 (1904-1946*)* | 1 | 1725 (1670-1782) | 0.86 |
| Southeast Asia 5 (SEA5) | 1957 (1939-1973) | 1 | 1711 (1654-1769) | 1 |
|  |  |  |  |  |
| **COSMOPOLITAN** | **1730 (1687-1773)** | **1** | **1615 (1549-1684)** | **1** |
| Africa 1 (AF1) | 1854 (1830-1877) | 1 | 1830 (1804-1857) | 1 |
| *Africa 1a (AF1a)* | *1872 (1851-1895)* | *1* | *1854 (1830-1877)* | *1* |
| *Africa 1b (AF1b)* | *1907 (1890-1925)* | *1* | *1855 (1832-1879)* | *0.64* |
| *Africa 1c (AF1c)* | *1983 (1980-1985)* | *1* | *1855 (1832-1879)* | *0.64* |
| Africa 4 (AF4) | 1932 (1923-1940) | 1 | 1730 (1687-1773) | 1 |
| America 1 (AM1) | 1908 (1886-1928) | 1 | 1799 (1767-1832) | 1 |
| America 2 (AM2) | 1815 (1781-1852) | 1 | 1756 (1717-1794) | 0.73 |
| *America 2a (AM2a)* | *1890 (1861-1920)* | *1* | *1815 (1781-1852)* | *1* |
| *America 2b (AM2b)* | *1830 (1781-1852)* | *1* | *1815 (1781-1852)* | *1* |
| America 3 (AM3) | 1836 (1804-1869) | 1 | 1753 (1711-1790) | 0.33 |
| *America 3a (AM3a)* | *1912 (1890-1936)* | *1* | *1836 (1804-1869)* | *1* |
| *America 3b (AM3b)* | *1890 (1863-1916)* | *1* | *1836 (1804-1869)* | *1* |
| America 4 (AM4) | 1846 (1811-1883) | 1 | 1751 (1711-1789) | 1 |
| Central Asia 1 (CA1) | 1946 (1936-1957) | 1 | 1892 (1875-1909) | 1 |
| Central Asia 2 (CA2) | 1944 (1927-1960) | 1 | 1833 (1807-1859) | 0.59 |
| Central Asia 3 (CA3) | 1942 (1926-1956) | 1 | 1892 (1875-1909) | 1 |
| Europe | 1895 (1877-1912) | 1 | 1888 (1872-1906) | 1 |
| *Central Europe (CE)* | *1967 (1961-1974)* | *1* | *1934 (1924-1945)* | *1* |
| *East Europe (EE)* | *1943 (1934-1954)* | *1* | *1915 (1902-1929)* | *1* |
| *West Europe (WE)* | *1949 (1942-1958)* | *1* | *1934 (1924-1945)* | *1* |
| *North East Europe (NEE)* | *1954 (1926-1964)* | *1* | *1895 (1877-1912)* | *1* |
| Middle East 1 (ME1) | 1919 (1905-1933) | 1 | 1876 (1858-1896) | 0,71 |
| *Middle East 1a (ME1a)* | *1938 (1927-1948)* | *1* | *1919 (1905-1933)* | *1* |
| *Middle East 1b (ME1b)* | *1987 (1984-1990)* | *1* | *1919 (1905-1933)* | *1* |
| Middle East 2 (ME2) | 1986 (1984-1989) | 1 | 1874 (1855-1894) | 1 |
|  |  |  |  |  |
| **INDIAN SUB.** | **1785 (1733-1840)** | **1** | **1459 (1355-1563)** | **0.77** |

The time of most recent common ancestor (TMRCA) of all clades, subclades and lineages from the dog-related RABV group, as defined in Fig.1, as for the nodes above, are indicated with the 95% high posterior density (HPD) estimates. The posterior probability values of each node are also indicated.
