# Supplementary material for: Large-Scale Phylogenomic Analysis Reveals the Complex Evolutionary History of Rabies Virus in Multiple Carnivore Hosts
Source: PLoS Pathog. 2016 Dec 15;12(12):e1006041. doi: 10.1371/journal.ppat.1006041 (PMC5158080; doi:10.1371/journal.ppat.1006041)
Supplement: S4 Table — (DOCX) [file ppat.1006041.s010.docx]

**Table S4: Substitution rates of the N and G genes in different host species in the dog-related RABV group.**

| **Data set** | **GTR+Γ+I** | **SDR06** | **HPM – GTR+Γ+I** | **HPM – SDR06** |
| --- | --- | --- | --- | --- |
| N-All | 2,18 x 10^-4^ (1,68 – 2,71) | 2,34 x 10^-4^ (1,80 – 2,91) | ND | ND |
| N-FB | 7,82 x 10^-4^ (3,13 - 13,17) | 8,00 x 10^-4^ (3,18 – 13,62) | 7,86 x 10^-4^ (2,92 – 13,26) | 7,70 x 10^-4^ (2,79 – 13,17) |
| N-MG-AF3 | 6,37 x 10^-4^ (3,95 – 8,87) | 6,54 x 10^-4^ (4,05 – 9,05) | 6,37 x 10^-4^ (3,94 – 8,88) | 6,52 x 10^-4^ (4,10 – 9,06) |
| N-MG-Caribbean | 3,31 x 10^-4^ (1,55 – 5,07) | 3,29 x 10^-4^ (1,61 – 5, 09) | 3,25 x 10^-4^ (1,65 – 4,98) | 3,26 x 10^-4^ (1,65 – 4,99) |
| N-Dog-Asia | 2,93 x 10^-4^ (1,44 – 4,39) | 3,08 x 10^-4^ (1,54 – 4,64) | 2,88 x 10^-4^ (1,39 – 4,43) | 3,09 x 10^-4^ (1,58 – 4,56) |
| N-Dog-Africa | 2,05 x 10^-4^ (1,26 – 2,87) | 2,03 x 10^-4^ (1,25 – 2,82) | 2,03 x 10^-4^ (1,21 – 2,82) | 2,02 x 10^-4^ (1,25 – 2,82) |
| G-All | 3,20 x 10^-4^ (2,60 – 3,80) | 3,40 x 10^-4^ (2,70 – 4,10) | ND | ND |
| G-FB | 6,43 x 10^-4^ (3,03 – 10,11) | 6,46 x 10^-4^ (2,93 – 10,21) | 6,13 x 10^-4^ (2,70 - 10,06) | 6,39 x 10^-4^ (2,88 – 10,01) |
| G-MG-AF3 | 2,74 x 10^-4^ (1,29 – 4,25) | 2,81 x 10^-4^ (1,34 – 4,31) | 2,70 x 10^-4^ (1,23 – 4,20) | 2,77 x 10^-4^ (1,28 – 4,33) |
| G-Dog-Asia | 3,40 x 10^-4^ (1,85 – 5,21) | 3,69 x 10^-4^ (1,97 – 5,51) | 3,51 x 10^-4^ (1,92 – 5,23) | 3,70 x 10^-4^ (1,93 – 5,53) |
| G-Dog-Africa | 2,90 x 10^-4^ (2,08 – 3,80) | 2,99 x 10^-4^ (2,16 – 3,88) | 2,92 x 10^-4^ (2,07 – 3,79) | 2,99 x 10^-4^ (2,15 – 3,83) |

The substitution rates in the nucleoprotein (N) and glycoprotein (G) genes of the dog-related group RABV, a sub-set of RABV circulating in mongooses (MG) in Africa-3 clade (AF3) or in the Caribbean area, in ferret-badgers (FB) in Asia, and in dogs in Asia and Africa. The substitution rates were estimated using two substitutions models the GTR+Γ+I and the SDR06 [1]. To assess the robustness of our rates estimates we also used a hierarchical phylogenetic model (HPM) [2]. Numbers in parentheses are the 95% highest posterior density around the median rate. ND = not done.

**REFERENCES:**

1. Shapiro B, Rambaut A, Drummond AJ. Choosing appropriate substitution models for the phylogenetic analysis of protein-coding sequences. Molecular biology and evolution. 2006;23(1):7-9. doi: 10.1093/molbev/msj021. PubMed PMID: 16177232.

2. Suchard MA, Kitchen CM, Sinsheimer JS, Weiss RE. Hierarchical phylogenetic models for analyzing multipartite sequence data. Systematic biology. 2003;52(5):649-64. PubMed PMID: 14530132.
