# Supplementary material for: Large-Scale Phylogenomic Analysis Reveals the Complex Evolutionary History of Rabies Virus in Multiple Carnivore Hosts
Source: PLoS Pathog. 2016 Dec 15;12(12):e1006041. doi: 10.1371/journal.ppat.1006041 (PMC5158080; doi:10.1371/journal.ppat.1006041)
Supplement: S5 Table — (DOCX) [file ppat.1006041.s011.docx]

**Table S5: Selection pressures in the N and G genes among different host species in the dog-related RABV group.**

|  | *d_N_/d_S_* | |
| --- | --- | --- |
|  | N | G |
| Dog-related group | **0.032** (n=254) | **0.099** (n=254) |
| Mongoose - AF3 | **0.090** (n=47) | **0.131** (n=32) |
| Mongoose - Caribbean | **0.047** (n=64) | **ND** |
| Ferret badger | **0.049** (n=81) | **0.123** (n=80) |
| Dog - Asia | **0.028** (n=51) | **0.089** (n=51) |
| Dog - Africa | **0.019** (n=46) | **0.089** (n=46) |

The *d_S_/d_N_* ratios are estimated using SLAC method. These values are calculated for the nucleoprotein (N) and glycoprotein (G) genes of the dog-related RABV group, a sub-set of RABV circulating in mongooses in Africa-3 clade or in Caribbean area, in ferret badgers in Asia and in dogs in Asia and Africa. ND = not done.
