# Supplementary material for: Large-Scale Phylogenomic Analysis Reveals the Complex Evolutionary History of Rabies Virus in Multiple Carnivore Hosts
Source: PLoS Pathog. 2016 Dec 15;12(12):e1006041. doi: 10.1371/journal.ppat.1006041 (PMC5158080; doi:10.1371/journal.ppat.1006041)
Supplement: S6 Table — (DOCX) [file ppat.1006041.s012.docx]

**Table S6: Amino acid substitutions specific to mongoose-related RABV (Africa-3 clade) or ferret-badger-related RABV (SEA5 subclade and the SEA2b lineage).**

| **Protein and codon position** | **Africa-3^a^** | **SEA5 and SEA2b^b^** |
| --- | --- | --- |
| **Nucleoprotein** |  |  |
| 88 | Asp -> Asn |  |
| 108 | Leu -> Ile |  |
| 374 |  | Leu -> Ser |
| **Glycoprotein** |  |  |
| 223 | Ser ^c^ -> Asn |  |
| 386 | Pro -> Ser |  |
| **Polymerase** |  |  |
| 200 |  | Lys -> Arg |

^a^ Comparison was performed with all dog-related RABV

^b^ Comparison was performed with all dog-related RABV belonging to the Asian clade

^c^ this amino acid is found in more than 79 % of the full length genome sequences of dog-related RABV
