# Supplementary material for: Large-Scale Phylogenomic Analysis Reveals the Complex Evolutionary History of Rabies Virus in Multiple Carnivore Hosts
Source: PLoS Pathog. 2016 Dec 15;12(12):e1006041. doi: 10.1371/journal.ppat.1006041 (PMC5158080; doi:10.1371/journal.ppat.1006041)
Supplement: S7 Table — (DOCX) [file ppat.1006041.s013.docx]

**Table S7: List of primers used in this study.**

| **Primer Name** | **Primer sequence (5’ to 3’)^a^** |
| --- | --- |
| N127 | F: ATGTAACACCTCTACAATGG |
| N8m | R: CAGTCTCYTCNGCCATCTC |
| N1304-S3 | F: AAYGGAGGTCGACTVAARAGATC |
| G3393-AS3 | R: CADGGRCCNAGYTTGTCTGGTAT |
| M220 | F: TGGTGTATCAACATGRAYTC |
| L1 | R: GAGTTNAGRTTGTARTCAGAG |
| G4836-S3 | F: GGRARRGTYATATCTTCNTGGGA |
| L7386-AS3 | R: CTRTCBGARTARTADAYCCANGACTT |
| PVO8 | R: GGTCTGATCTRTCWGARYAATA |
| Taq3long | F: ATGAGAAGTGGAAYAAYCATCA |
| L9633-AS3 | R: TGCYRTATATGTTGACAGG |
| L9267-S3 | F: ATGTTYCAGCCNTTGATGCT |
| L9129-S3 | R: TCNGCCTTGCAYAGGTTCAA |
| L11872-AS3 | R: AAAYAATCAARCARHCAGAGG |
| L9267-S2 | F: ATGTTYCARCCNYTGATGCT |
| L9129-S2 | F: TCNGCNYTRCAYAGGTTCAA |
| L11872-AS2 | R: AAAYAATCAADCARHYAGAGG |
| N1304-S2 | F: AAYGGRGGBMGAYTVAARAGATC |
| G3393-AS2 | R: CADGGDCCNAGYTTGTCTGGTAT |
| N1309-AF2-S | F: AGGGCGACTVAAGAGATCTCACAT |
| G3424-AF2-AS | R: TGGACAGCTGAGRTGATGTATGTC |
| L9267-MADA-S1 | F: ATGTTYCAGCCRYTGATGCT |
| L11872-MADA-AS1 | R: AAAYAATCAANCAGCCAGAGG |
| L9267-9702-S1 | F: ATGTTCCARCCRYTGATGCT |
| L11872-9702-AS1 | R: AAACAATCAAACAATCAGAGG |
| L9267-9001FRA-S1 | F: ATGTTCCAACCATTGATGCT |
| L11872-9001FRA-AS1 | R: AAACAATCGAGCCGACAGAGG |

^a^ F: Forward; R: reverse
