# Supplementary material for: Large-Scale Phylogenomic Analysis Reveals the Complex Evolutionary History of Rabies Virus in Multiple Carnivore Hosts
Source: PLoS Pathog. 2016 Dec 15;12(12):e1006041. doi: 10.1371/journal.ppat.1006041 (PMC5158080; doi:10.1371/journal.ppat.1006041)
Supplement: S8 Table — (DOCX) [file ppat.1006041.s014.docx]

**Table S8: List of additional nucleotide and glycoprotein sequences used to estimate the evolution rates among the different hosts.**

| **Group** | **Gene** | **Accession Number** | **Virus Name** | **Date** | **Country** | **Reference** |
| --- | --- | --- | --- | --- | --- | --- |
| Mongoose – AF3 | Nucleoprotein | FJ392391 | 22017 | 1994 | Zimbabwe | (1) |
| Mongoose – AF3 | Nucleoprotein | FJ392367 | 22/01 | 2001 | South Africa | (1) |
| Mongoose – AF3 | Nucleoprotein | FJ392392 | SN0080 | 1980 | Namibia | (1) |
| Mongoose – AF3 | Nucleoprotein | FJ392382 | 389/02 | 2002 | South Africa | (1) |
| Mongoose – AF3 | Nucleoprotein | FJ392372 | 113/91 | 1991 | South Africa | (1) |
| Mongoose – AF3 | Nucleoprotein | FJ392386 | 718/98 | 1998 | South Africa | (1) |
| Mongoose – AF3 | Nucleoprotein | FJ392374 | 211/98 | 1998 | South Africa | (1) |
| Mongoose – AF3 | Nucleoprotein | FJ392381 | 385/06 | 2006 | South Africa | (1) |
| Mongoose – AF3 | Nucleoprotein | JQ692994 | 696-06 | 2006 | South Africa | Ngoepe *et al.,* unpublished |
| Mongoose – AF3 | Nucleoprotein | JQ692985 | 18-06 | 2006 | South Africa | Ngoepe *et al.,* unpublished |
| Mongoose – AF3 | Nucleoprotein | FJ392380 | 381/06 | 2006 | South Africa | (1) |
| Mongoose – AF3 | Nucleoprotein | FJ392373 | 155/03 | 2003 | South Africa | (1) |
| Mongoose – AF3 | Nucleoprotein | JQ692996 | 13-07 | 2007 | South Africa | Ngoepe *et al.,* unpublished |
| Mongoose – AF3 | Nucleoprotein | JQ692992 | 265-06 | 2006 | South Africa | Ngoepe *et al.,* unpublished |
| Mongoose – AF3 | Nucleoprotein | FJ392378 | 344/99 | 1999 | South Africa | (1) |
| Mongoose – AF3 | Nucleoprotein | FJ392377 | 279/99 | 1999 | South Africa | (1) |
| Mongoose – AF3 | Nucleoprotein | JQ692987 | 27-08 | 2008 | South Africa | Ngoepe *et al.,* unpublished |
| Mongoose – AF3 | Nucleoprotein | FJ392383 | 420/90 | 1990 | South Africa | (1) |
| Mongoose – AF3 | Nucleoprotein | JQ692982 | 06-07 | 2007 | South Africa | Ngoepe *et al.,* unpublished |
| Mongoose – AF3 | Nucleoprotein | FJ392371 | 32/02 | 2002 | South Africa | (1) |
| Mongoose – AF3 | Nucleoprotein | JQ692990 | 57-06 | 2006 | South Africa | Ngoepe *et al.,* unpublished |
| Mongoose – AF3 | Nucleoprotein | JQ692988 | 456-06 | 2006 | South Africa | Ngoepe *et al.,* unpublished |
| Mongoose – AF3 | Nucleoprotein | FJ392389 | 878/92 | 1992 | South Africa | (1) |
| Mongoose – AF3 | Nucleoprotein | FJ392388 | 767/95 | 1995 | South Africa | (1) |
| Mongoose – AF3 | Nucleoprotein | FJ392384 | 485/94 | 1994 | South Africa | (1) |
| Mongoose – AF3 | Nucleoprotein | FJ392376 | 228/01 | 2001 | South Africa | (1) |
| Mongoose – AF3 | Nucleoprotein | FJ392369 | 28/00 | 2000 | South Africa | (1) |
| Mongoose – AF3 | Nucleoprotein | FJ392368 | 23/01 | 2001 | South Africa | (1) |
| Mongoose – AF3 | Nucleoprotein | JQ692991 | 956-06 | 2006 | South Africa | Ngoepe *et al.,* unpublished |
| Mongoose – AF3 | Nucleoprotein | FJ392379 | 364/96 | 1996 | South Africa | (1) |
| Mongoose – AF3 | Nucleoprotein | FJ392366 | 19/92 | 1992 | South Africa | (1) |
| Mongoose – AF3 | Nucleoprotein | JQ692986 | 200-06 | 2006 | South Africa | Ngoepe *et al.,* unpublished |
| Mongoose – AF3 | Nucleoprotein | JQ692995 | 257-05 | 2005 | South Africa | Ngoepe *et al.,* unpublished |
| Mongoose – AF3 | Nucleoprotein | JQ692993 | 418-06 | 2006 | South Africa | (1) |
| Mongoose – AF3 | Nucleoprotein | FJ392370 | 30/00 | 2000 | South Africa | (1) |
| Mongoose – AF3 | Nucleoprotein | FJ392375 | 221/98 | 1998 | South Africa | (1) |
| Mongoose – AF3 | Nucleoprotein | FJ392387 | 759/96 | 1996 | South Africa | (1) |
| Mongoose – AF3 | Nucleoprotein | JQ692983 | 116-08 | 2008 | South Africa | Ngoepe *et al.,* unpublished |
| Mongoose – AF3 | Nucleoprotein | FJ392390 | 926/93 | 1993 | South Africa | (1) |
| Mongoose – AF3 | Nucleoprotein | FJ392385 | 669/90 | 1990 | South Africa | (1) |
| Mongoose – AF3 | Nucleoprotein | JQ692989 | 50-06 | 2006 | South Africa | Ngoepe *et al.,* unpublished |
| Mongoose – AF3 | Glycoprotein | FJ465385 | 22/01 | 2001 | South Africa | (1) |
| Mongoose – AF3 | Glycoprotein | FJ465408 | 22017 | 1994 | Zimbabwe | (1) |
| Mongoose – AF3 | Glycoprotein | FJ465392 | 211/98 | 1998 | South Africa | (1) |
| Mongoose – AF3 | Glycoprotein | FJ465403 | 718/98 | 1998 | South Africa | (1) |
| Mongoose – AF3 | Glycoprotein | FJ465400 | 389/02 | 2002 | South Africa | (1) |
| Mongoose – AF3 | Glycoprotein | FJ465409 | SN0080 | 1980 | Namibia | (1) |
| Mongoose – AF3 | Glycoprotein | FJ465390 | 113/91 | 1991 | South Africa | (1) |
| Mongoose – AF3 | Glycoprotein | FJ465399 | 385/06 | 2006 | South Africa | (1) |
| Mongoose – AF3 | Glycoprotein | FJ465391 | 155/03 | 2003 | South Africa | (1) |
| Mongoose – AF3 | Glycoprotein | FJ465398 | 381/06 | 2006 | South Africa | (1) |
| Mongoose – AF3 | Glycoprotein | FJ465401 | 420/90 | 1990 | South Africa | (1) |
| Mongoose – AF3 | Glycoprotein | FJ465395 | 279/99 | 1999 | South Africa | (1) |
| Mongoose – AF3 | Glycoprotein | FJ465396 | 344/99 | 1999 | South Africa | (1) |
| Mongoose – AF3 | Glycoprotein | FJ465389 | 32/02 | 2002 | South Africa | (1) |
| Mongoose – AF3 | Glycoprotein | FJ465406 | 878/92 | 1992 | South Africa | (1) |
| Mongoose – AF3 | Glycoprotein | FJ465405 | 767/95 | 1995 | South Africa | (1) |
| Mongoose – AF3 | Glycoprotein | FJ465387 | 28/00 | 2000 | South Africa | (1) |
| Mongoose – AF3 | Glycoprotein | FJ465394 | 228/01 | 2001 | South Africa | (1) |
| Mongoose – AF3 | Glycoprotein | FJ465386 | 23/01 | 2001 | South Africa | (1) |
| Mongoose – AF3 | Glycoprotein | FJ465384 | 19/92 | 1992 | South Africa | (1) |
| Mongoose – AF3 | Glycoprotein | FJ465397 | 364/96 | 1996 | South Africa | (1) |
| Mongoose – AF3 | Glycoprotein | FJ465388 | 30/00 | 2000 | South Africa | (1) |
| Mongoose – AF3 | Glycoprotein | FJ465393 | 221/98 | 1998 | South Africa | (1) |
| Mongoose – AF3 | Glycoprotein | FJ465404 | 759/96 | 1996 | South Africa | (1) |
| Mongoose – AF3 | Glycoprotein | FJ465407 | 926/93 | 1993 | South Africa | (1) |
| Mongoose – AF3 | Glycoprotein | FJ465402 | 669/90 | 1990 | South Africa | (1) |
| Mongoose – Caribbean | Nucleoprotein | AY854576 | V1040 | 2006 | Cuba | (2) |
| Mongoose – Caribbean | Nucleoprotein | AY854575 | V1039 | 2000 | Cuba | (2) |
| Mongoose – Caribbean | Nucleoprotein | AY854573 | V1090 | 2001 | Cuba | (2) |
| Mongoose – Caribbean | Nucleoprotein | AY854569 | V1124 | 2002 | Cuba | (2) |
| Mongoose – Caribbean | Nucleoprotein | AY854567 | V1036 | 2000 | Cuba | (2) |
| Mongoose – Caribbean | Nucleoprotein | AY854574 | V1072 | 2001 | Cuba | (2) |
| Mongoose – Caribbean | Nucleoprotein | AY854564 | V1178 | 2002 | Cuba | (2) |
| Mongoose – Caribbean | Nucleoprotein | AY854562 | V1061 | 2000 | Cuba | (2) |
| Mongoose – Caribbean | Nucleoprotein | AY854561 | V1115 | 2002 | Cuba | (2) |
| Mongoose – Caribbean | Nucleoprotein | AY854560 | V1114 | 2002 | Cuba | (2) |
| Mongoose – Caribbean | Nucleoprotein | AY854578 | V1095 | 2001 | Cuba | (2) |
| Mongoose – Caribbean | Nucleoprotein | AY854554 | V1127 | 2002 | Cuba | (2) |
| Mongoose – Caribbean | Nucleoprotein | AY854552 | V1069 | 2000 | Cuba | (2) |
| Mongoose – Caribbean | Nucleoprotein | AY854558 | V1101 | 2002 | Cuba | (2) |
| Mongoose – Caribbean | Nucleoprotein | AY854559 | V1129 | 2002 | Cuba | (2) |
| Mongoose – Caribbean | Nucleoprotein | AY854544 | V1067 | 2000 | Cuba | (2) |
| Mongoose – Caribbean | Nucleoprotein | AY854545 | V1065 | 2000 | Cuba | (2) |
| Mongoose – Caribbean | Nucleoprotein | AY854547 | V1125 | 2002 | Cuba | (2) |
| Mongoose – Caribbean | Nucleoprotein | AY854546 | V1122 | 2002 | Cuba | (2) |
| Mongoose – Caribbean | Nucleoprotein | AY854536 | V1056 | 2000 | Cuba | (2) |
| Mongoose – Caribbean | Nucleoprotein | AY854548 | V1088 | 2001 | Cuba | (2) |
| Mongoose – Caribbean | Nucleoprotein | AY854530 | V1052 | 2000 | Cuba | (2) |
| Mongoose – Caribbean | Nucleoprotein | AY854529 | V1085 | 2001 | Cuba | (2) |
| Mongoose – Caribbean | Nucleoprotein | AY854534 | V1077 | 2001 | Cuba | (2) |
| Mongoose – Caribbean | Nucleoprotein | AY854531 | V1121 | 2002 | Cuba | (2) |
| Mongoose – Caribbean | Nucleoprotein | AY854527 | V1050 | 2002 | Cuba | (2) |
| Mongoose – Caribbean | Nucleoprotein | AY854525 | V1075 | 2001 | Cuba | (2) |
| Mongoose – Caribbean | Nucleoprotein | AY854524 | V1109 | 2002 | Cuba | (2) |
| Mongoose – Caribbean | Nucleoprotein | AY854506 | V1082 | 2001 | Cuba | (2) |
| Mongoose – Caribbean | Nucleoprotein | AY854504 | V1044 | 2000 | Cuba | (2) |
| Mongoose – Caribbean | Nucleoprotein | AY854515 | V1074 | 2001 | Cuba | (2) |
| Mongoose – Caribbean | Nucleoprotein | AY854520 | V1103 | 2002 | Cuba | (2) |
| Mongoose – Caribbean | Nucleoprotein | AY854507 | V1139 | 2000 | Cuba | (2) |
| Mongoose – Caribbean | Nucleoprotein | AY854505 | V1079 | 2001 | Cuba | (2) |
| Mongoose – Caribbean | Nucleoprotein | KJ957438 | RV2854N | 2011 | Grenada | (3) |
| Mongoose – Caribbean | Nucleoprotein | KJ957437 | RV2853N | 2011 | Grenada | (3) |
| Mongoose – Caribbean | Nucleoprotein | KJ957434 | RV2850N | 2011 | Grenada | (3) |
| Mongoose – Caribbean | Nucleoprotein | KM067274 | RV2926 | 2012 | Grenada | (3) |
| Mongoose – Caribbean | Nucleoprotein | KJ957452 | RV2873N | 2013 | Grenada | (3) |
| Mongoose – Caribbean | Nucleoprotein | KJ957444 | RV2965N | 2012 | Grenada | (3) |
| Mongoose – Caribbean | Nucleoprotein | KJ957442 | RV2928N | 2012 | Grenada | (3) |
| Mongoose – Caribbean | Nucleoprotein | KJ957451 | RV2972N | 2013 | Grenada | (3) |
| Mongoose – Caribbean | Nucleoprotein | KJ957433 | RV2849N | 2011 | Grenada | (3) |
| Mongoose – Caribbean | Nucleoprotein | KJ957449 | RV2970N | 2013 | Grenada | (3) |
| Mongoose – Caribbean | Nucleoprotein | KJ957445 | RV2966N | 2012 | Grenada | (3) |
| Mongoose – Caribbean | Nucleoprotein | KJ957450 | RV2971N | 2013 | Grenada | (3) |
| Mongoose – Caribbean | Nucleoprotein | KJ957440 | RV2925N | 2011 | Grenada | (3) |
| Mongoose – Caribbean | Nucleoprotein | KJ957432 | RV2848N | 2011 | Grenada | (3) |
| Mongoose – Caribbean | Nucleoprotein | KJ957436 | RV2852N | 2011 | Grenada | (3) |
| Mongoose – Caribbean | Nucleoprotein | KJ957431 | RV2847N | 2011 | Grenada | (3) |
| Mongoose – Caribbean | Nucleoprotein | KJ957441 | RV2927N | 2012 | Grenada | (3) |
| Mongoose – Caribbean | Nucleoprotein | KJ957435 | RV2851N | 2011 | Grenada | (3) |
| Mongoose – Caribbean | Nucleoprotein | KJ957443 | RV2964N | 2012 | Grenada | (3) |
| Mongoose – Caribbean | Nucleoprotein | FJ228497 | 2840PuertoRicodg2006 | 2006 | Puerto Rico | Velasco-Villa *et al.,* unpublished |
| Mongoose – Caribbean | Nucleoprotein | JQ513538 | V525 | 1997 | Puerto Rico | Nadin-Davis *et al.,* unpublished |
| Mongoose – Caribbean | Nucleoprotein | FJ228495 | Prmongoose2004 | 2004 | Puerto Rico | Velasco-Villa *et al.,* unpublished |
| Mongoose – Caribbean | Nucleoprotein | FJ228496 | 2839dgPuertoRico2006 | 2006 | Puerto Rico | Velasco-Villa *et al.,* unpublished |
| Mongoose – Caribbean | Nucleoprotein | JQ513535 | V520 | 1997 | Puerto Rico | Nadin-Davis *et al.,* unpublished |
| Mongoose – Caribbean | Nucleoprotein | JQ513537 | V524 | 1997 | Puerto Rico | Nadin-Davis *et al.,* unpublished |
| Mongoose – Caribbean | Nucleoprotein | JQ513528 | V470 | 1996 | Puerto Rico | Nadin-Davis *et al.,* unpublished |
| Mongoose – Caribbean | Nucleoprotein | JQ513536 | V522 | 1997 | Puerto Rico | Nadin-Davis *et al.,* unpublished |
| Mongoose – Caribbean | Nucleoprotein | JQ513534 | V516 | 1997 | Puerto Rico | Nadin-Davis *et al.,* unpublished |
| Mongoose – Caribbean | Nucleoprotein | JQ513533 | V514 | 1997 | Puerto Rico | Nadin-Davis *et al.,* unpublished |
| Mongoose – Caribbean | Nucleoprotein | FJ228498 | 4355Perudg2004 | 2004 | Peru | Velasco-Villa *et al.,* unpublished |
| Ferret Badger | Nucleoprotein | JQ950452 | JX12-234 | Feb-2012 | China: Jiangxi | Zhao *et al.,* unpublished |
| Ferret Badger | Nucleoprotein | JQ950450 | JX12-102 | Feb-2012 | China: Jiangxi | Zhao *et al.,* unpublished |
| Ferret Badger | Nucleoprotein | JQ950448 | JX12-67 | Feb-2012 | China: Jiangxi | Zhao *et al.,* unpublished |
| Ferret Badger | Nucleoprotein | FJ719755 | JX08-58 | Nov-2008 | China: Jiangxi | Zhang *et al,* unpublished |
| Ferret Badger | Nucleoprotein | FJ719753 | JX08-48 | Nov-2008 | China: Jiangxi | Zhang *et al.* unpublished |
| Ferret Badger | Nucleoprotein | HQ118118 | ZJF5 | 2008 | China: Zhejiang | (4) |
| Ferret Badger | Nucleoprotein | HQ118117 | ZJF4 | 2008 | China: Zhejiang | (4) |
| Ferret Badger | Nucleoprotein | HQ118116 | ZJF3 | 2008 | China: Zhejiang | (4) |
| Ferret Badger | Nucleoprotein | HQ118115 | ZJF2 | 2008 | China: Zhejiang | (4) |
| Ferret Badger | Nucleoprotein | HQ118114 | ZJF1 | 2008 | China: Zhejiang | (4) |
| Ferret Badger | Nucleoprotein | FJ598135 | ZJ-LA | Oct-2008 | China | Zhang *et al.,* unpublished |
| Ferret Badger | Nucleoprotein | JN974877 | CZJ0803F | 2008 | China | (5) |
| Ferret Badger | Nucleoprotein | FJ719751 | JX08-47 | Nov-2008 | China: Jiangxi | Zhang *et al.,* unpublished |
| Ferret Badger | Nucleoprotein | KF501183 | TW-1683 | Jul-2013 | Taiwan: Nantou | Tsai *et al.,* unpublished |
| Ferret Badger | Nucleoprotein | KF501182 | TW-1682 | Jul-2013 | Taiwan: Nantou | Tsai *et al.,* unpublished |
| Ferret Badger | Nucleoprotein | KF501181 | TW-1680 | Jul-2013 | Taiwan: Taichung | Tsai *et al.,* unpublished |
| Ferret Badger | Nucleoprotein | KF501185 | TW-1694 | Jul-2013 | Taiwan: Kaohsiung | Tsai *et al.,* unpublished |
| Ferret Badger | Nucleoprotein | KF501184 | TW-1685 | Jul-2013 | Taiwan: Tainan | Tsai *et al.,* unpublished |
| Ferret Badger | Nucleoprotein | KF501180 | TW-1614 | Jul-2013 | Taiwan: Taitung | Tsai *et al.,* unpublished |
| Ferret Badger | Nucleoprotein | KP860137 | Cy2143 | Aug-2013 | Taiwan: Chiayi | (6) |
| Ferret Badger | Nucleoprotein | KP860138 | Cy2263 | Aug-2013 | Taiwan: Chiayi | (6) |
| Ferret Badger | Nucleoprotein | KP860139 | Cy2717 | Mar-2013 | Taiwan: Chiayi | (6) |
| Ferret Badger | Nucleoprotein | KP860140 | HL1956 | Aug-2013 | Taiwan: Hualien | (6) |
| Ferret Badger | Nucleoprotein | KP860141 | Kh1710 | Jul-2013 | Taiwan: Kaohsiung | (6) |
| Ferret Badger | Nucleoprotein | KP860142 | Kh1830 | Jul-2013 | Taiwan: Kaohsiung | (6) |
| Ferret Badger | Nucleoprotein | KP860143 | Kh1879 | Aug-2013 | Taiwan: Kaohsiung | (6) |
| Ferret Badger | Nucleoprotein | KP860144 | Kh2261 | Aug-2013 | Taiwan: Kaohsiung | (6) |
| Ferret Badger | Nucleoprotein | KP860145 | Kh2774 | Aug-2013 | Taiwan: Kaohsiung | (6) |
| Ferret Badger | Nucleoprotein | KP860146 | Kh3206 | Sep-2013 | Taiwan: Kaohsiung | (6) |
| Ferret Badger | Nucleoprotein | KP860147 | Kh3597 | Sep-2013 | Taiwan: Kaohsiung | (6) |
| Ferret Badger | Nucleoprotein | KP860148 | Kh3975 | Oct-2013 | Taiwan: Kaohsiung | (6) |
| Ferret Badger | Nucleoprotein | KP860149 | Nt1938 | Aug-2013 | Taiwan: Nantou | (6) |
| Ferret Badger | Nucleoprotein | KP860150 | Nt1983 | Aug-2013 | Taiwan: Nantou | (6) |
| Ferret Badger | Nucleoprotein | KP860151 | Nt2169 | Aug-2013 | Taiwan: Nantou | (6) |
| Ferret Badger | Nucleoprotein | KP860152 | Nt2269 | Aug-2013 | Taiwan: Nantou | (6) |
| Ferret Badger | Nucleoprotein | KP860153 | Nt2270 | Aug-2013 | Taiwan: Nantou | (6) |
| Ferret Badger | Nucleoprotein | KP860154 | Nt2274 | Aug-2013 | Taiwan: Nantou | (6) |
| Ferret Badger | Nucleoprotein | KP860155 | Nt2702 | Jul-2010 | Taiwan: Nantou | (6) |
| Ferret Badger | Nucleoprotein | KP860156 | Nt2704 | Jul-2010 | Taiwan: Nantou | (6) |
| Ferret Badger | Nucleoprotein | KP860157 | Nt2706 | Aug-2012 | Taiwan: Nantou | (6) |
| Ferret Badger | Nucleoprotein | KP860158 | Nt2710 | Jan-2013 | Taiwan: Nantou | (6) |
| Ferret Badger | Nucleoprotein | KP860159 | Nt4951 | Nov-2013 | Taiwan: Nantou | (6) |
| Ferret Badger | Nucleoprotein | KP860160 | Pt2485 | Aug-2013 | Taiwan: Pingtung | (6) |
| Ferret Badger | Nucleoprotein | KP860161 | Pt2946 | Sep-2013 | Taiwan: Pingtung | (6) |
| Ferret Badger | Nucleoprotein | KP860162 | Pt3977 | Oct-2013 | Taiwan: Pingtung | (6) |
| Ferret Badger | Nucleoprotein | KP860163 | Th1749 | Jul-2013 | Taiwan: Taichung | (6) |
| Ferret Badger | Nucleoprotein | KP860164 | Th2170 | Aug-2013 | Taiwan: Taichung | (6) |
| Ferret Badger | Nucleoprotein | KP860165 | Th2229 | Aug-2013 | Taiwan: Taichung | (6) |
| Ferret Badger | Nucleoprotein | KP860166 | Th2284 | Aug-2013 | Taiwan: Taichung | (6) |
| Ferret Badger | Nucleoprotein | KP860167 | Th2408 | Aug-2013 | Taiwan: Taichung | (6) |
| Ferret Badger | Nucleoprotein | KP860168 | Th2484 | Aug-2013 | Taiwan: Taichung | (6) |
| Ferret Badger | Nucleoprotein | KP860169 | Th4957 | Dec-2013 | Taiwan: Taichung | (6) |
| Ferret Badger | Nucleoprotein | KP860170 | Th5114 | Dec-2013 | Taiwan: Taichung | (6) |
| Ferret Badger | Nucleoprotein | KP860171 | Tn1766 | Jul-2013 | Taiwan: Tainan | (6) |
| Ferret Badger | Nucleoprotein | KP860172 | Tn1832 | Jul-2013 | Taiwan: Tainan | (6) |
| Ferret Badger | Nucleoprotein | KP860173 | Tn1950 | Aug-2013 | Taiwan: Tainan | (6) |
| Ferret Badger | Nucleoprotein | KP860174 | Tn2058 | Aug-2013 | Taiwan: Tainan | (6) |
| Ferret Badger | Nucleoprotein | KP860175 | Tn2392 | Aug-2013 | Taiwan: Tainan | (6) |
| Ferret Badger | Nucleoprotein | KP860176 | Tn2740 | Aug-2013 | Taiwan: Tainan | (6) |
| Ferret Badger | Nucleoprotein | KP860177 | Tn3106 | Sep-2013 | Taiwan: Tainan | (6) |
| Ferret Badger | Nucleoprotein | KP860178 | Tt2329 | Aug-2013 | Taiwan: Taitung | (6) |
| Ferret Badger | Nucleoprotein | KP860179 | Tt2514 | Aug-2013 | Taiwan: Taitung | (6) |
| Ferret Badger | Nucleoprotein | KP860180 | Tt4931 | Nov-2013 | Taiwan: Taitung | (6) |
| Ferret Badger | Nucleoprotein | KP860181 | Tt5037 | Dec-2013 | Taiwan: Taitung | (6) |
| Ferret Badger | Nucleoprotein | KP860182 | Tt5040 | Dec-2013 | Taiwan: Taitung | (6) |
| Ferret Badger | Nucleoprotein | KP860183 | Tt5249 | Dec-2013 | Taiwan: Taitung | (6) |
| Ferret Badger | Nucleoprotein | KP860184 | YL2167 | Aug-2013 | Taiwan: Yunlin | (6) |
| Ferret Badger | Nucleoprotein | KP860185 | YL5154 | Dec-2013 | Taiwan: Yunlin | (6) |
| Ferret Badger | Nucleoprotein | KP881353 | TW-1955 | Aug-2013 | Taiwan: Hualien | (6) |
| Ferret Badger | Nucleoprotein | KP881354 | TW-2700 | Aug-2013 | Taiwan: Nantou | (6) |
| Ferret Badger | Nucleoprotein | KP881355 | TW-1907 | Aug-2013 | Taiwan: Taichung | (6) |
| Ferret Badger | Nucleoprotein | KP881356 | TW-1944 | Aug-2013 | Taiwan: Yunlin | (6) |
| Ferret Badger | Glycoprotein | JQ950453 | JX12-234 | Feb-2012 | China: Jiangxi | Zhao *et al.,* unpublished |
| Ferret Badger | Glycoprotein | JQ950451 | JX12-102 | Feb-2012 | China: Jiangxi | Zhao *et al.,* unpublished |
| Ferret Badger | Glycoprotein | JQ950449 | JX12-67 | Feb-2012 | China: Jiangxi | Zhao *et al.,* unpublished |
| Ferret Badger | Glycoprotein | FJ719749 | JX08-47 | Nov-2008 | China: Jiangxi | Zhang *et al.,* unpublished |
| Ferret Badger | Glycoprotein | FJ719752 | JX08-48 | Nov-2008 | China: Jiangxi | Zhang *et al.,* unpublished |
| Ferret Badger | Glycoprotein | GQ857469 | 08ZL13 | 2008 | China: Zhejiang | (7) |
| Ferret Badger | Glycoprotein | GQ857468 | 08ZL11 | 2008 | China: Zhejiang | (7) |
| Ferret Badger | Glycoprotein | FJ825135 | F05 | 2008 | China: Zhejiang | (7) |
| Ferret Badger | Glycoprotein | FJ825134 | F03 | 2008 | China: Zhejiang | (7) |
| Ferret Badger | Glycoprotein | FJ719756 | ZJ-LA | Oct-2008 | China | Zhang *et al.,* unpublished |
| Ferret Badger | Glycoprotein | JN936788 | CZJ0803F | 2008 | China | (8) |
| Ferret Badger | Glycoprotein | KF501177 | TW-1683 | Jul-2013 | Taiwan: Nantou | Tsai *et al.,* unpublished |
| Ferret Badger | Glycoprotein | KF501176 | TW-1682 | Jul-2013 | Taiwan: Nantou | Tsai *et al.,* unpublished |
| Ferret Badger | Glycoprotein | KF501175 | TW-1680 | Jul-2013 | Taiwan: Taichung | Tsai *et al.,* unpublished |
| Ferret Badger | Glycoprotein | KF501179 | TW-1694 | Jul-2013 | Taiwan: Kaohsiung | Tsai *et al.,* unpublished |
| Ferret Badger | Glycoprotein | KF501178 | TW-1685 | Jul-2013 | Taiwan: Tainan | Tsai *et al.,* unpublished |
| Ferret Badger | Glycoprotein | KF501174 | TW-1614 | Jul-2013 | Taiwan: Taitung | Tsai *et al.,* unpublished |
| Ferret Badger | Glycoprotein | FJ825133 | F01 | 2008 | China | (7) |
| Ferret Badger | Glycoprotein | KP860186 | Cy2143 | Aug-2013 | Taiwan: Chiayi | (6) |
| Ferret Badger | Glycoprotein | KP860187 | Cy2263 | Aug-2013 | Taiwan: Chiayi | (6) |
| Ferret Badger | Glycoprotein | KP860188 | Cy2717 | Mar-2013 | Taiwan: Chiayi | (6) |
| Ferret Badger | Glycoprotein | KP860189 | HL1956 | Aug-2013 | Taiwan: Hualien | (6) |
| Ferret Badger | Glycoprotein | KP860190 | Kh1710 | Jul-2013 | Taiwan: Kaohsiung | (6) |
| Ferret Badger | Glycoprotein | KP860191 | Kh1830 | Jul-2013 | Taiwan: Kaohsiung | (6) |
| Ferret Badger | Glycoprotein | KP860192 | Kh1879 | Aug-2013 | Taiwan: Kaohsiung | (6) |
| Ferret Badger | Glycoprotein | KP860193 | Kh2261 | Aug-2013 | Taiwan: Kaohsiung | (6) |
| Ferret Badger | Glycoprotein | KP860194 | Kh2774 | Aug-2013 | Taiwan: Kaohsiung | (6) |
| Ferret Badger | Glycoprotein | KP860195 | Kh3206 | Sep-2013 | Taiwan: Kaohsiung | (6) |
| Ferret Badger | Glycoprotein | KP860196 | Kh3597 | Sep-2013 | Taiwan: Kaohsiung | (6) |
| Ferret Badger | Glycoprotein | KP860197 | Kh3975 | Oct-2013 | Taiwan: Kaohsiung | (6) |
| Ferret Badger | Glycoprotein | KP860198 | Nt1938 | Aug-2013 | Taiwan: Nantou | (6) |
| Ferret Badger | Glycoprotein | KP860199 | Nt1983 | Aug-2013 | Taiwan: Nantou | (6) |
| Ferret Badger | Glycoprotein | KP860200 | Nt2169 | Aug-2013 | Taiwan: Nantou | (6) |
| Ferret Badger | Glycoprotein | KP860201 | Nt2269 | Aug-2013 | Taiwan: Nantou | (6) |
| Ferret Badger | Glycoprotein | KP860202 | Nt2270 | Aug-2013 | Taiwan: Nantou | (6) |
| Ferret Badger | Glycoprotein | KP860203 | Nt2274 | Aug-2013 | Taiwan: Nantou | (6) |
| Ferret Badger | Glycoprotein | KP860204 | Nt2702 | Jul-2010 | Taiwan: Nantou | (6) |
| Ferret Badger | Glycoprotein | KP860205 | Nt2704 | Jul-2010 | Taiwan: Nantou | (6) |
| Ferret Badger | Glycoprotein | KP860206 | Nt2706 | Aug-2012 | Taiwan: Nantou | (6) |
| Ferret Badger | Glycoprotein | KP860207 | Nt2710 | Jan-2013 | Taiwan: Nantou | (6) |
| Ferret Badger | Glycoprotein | KP860208 | Nt4951 | Nov-2013 | Taiwan: Nantou | (6) |
| Ferret Badger | Glycoprotein | KP860209 | Pt2485 | Aug-2013 | Taiwan: Pingtung | (6) |
| Ferret Badger | Glycoprotein | KP860210 | Pt2946 | Sep-2013 | Taiwan: Pingtung | (6) |
| Ferret Badger | Glycoprotein | KP860211 | Pt3977 | Oct-2013 | Taiwan: Pingtung | (6) |
| Ferret Badger | Glycoprotein | KP860212 | Th1749 | Jul-2013 | Taiwan: Taichung | (6) |
| Ferret Badger | Glycoprotein | KP860213 | Th2170 | Aug-2013 | Taiwan: Taichung | (6) |
| Ferret Badger | Glycoprotein | KP860214 | Th2229 | Aug-2013 | Taiwan: Taichung | (6) |
| Ferret Badger | Glycoprotein | KP860215 | Th2284 | Aug-2013 | Taiwan: Taichung | (6) |
| Ferret Badger | Glycoprotein | KP860216 | Th2408 | Aug-2013 | Taiwan: Taichung | (6) |
| Ferret Badger | Glycoprotein | KP860217 | Th2484 | Aug-2013 | Taiwan: Taichung | (6) |
| Ferret Badger | Glycoprotein | KP860218 | Th4957 | Dec-2013 | Taiwan: Taichung | (6) |
| Ferret Badger | Glycoprotein | KP860219 | Th5114 | Dec-2013 | Taiwan: Taichung | (6) |
| Ferret Badger | Glycoprotein | KP860220 | Tn1766 | Jul-2013 | Taiwan: Tainan | (6) |
| Ferret Badger | Glycoprotein | KP860221 | Tn1832 | Jul-2013 | Taiwan: Tainan | (6) |
| Ferret Badger | Glycoprotein | KP860222 | Tn1950 | Aug-2013 | Taiwan: Tainan | (6) |
| Ferret Badger | Glycoprotein | KP860223 | Tn2058 | Aug-2013 | Taiwan: Tainan | (6) |
| Ferret Badger | Glycoprotein | KP860224 | Tn2392 | Aug-2013 | Taiwan: Tainan | (6) |
| Ferret Badger | Glycoprotein | KP860225 | Tn2740 | Aug-2013 | Taiwan: Tainan | (6) |
| Ferret Badger | Glycoprotein | KP860226 | Tn3106 | Sep-2013 | Taiwan: Tainan | (6) |
| Ferret Badger | Glycoprotein | KP860227 | Tt2329 | Aug-2013 | Taiwan: Taitung | (6) |
| Ferret Badger | Glycoprotein | KP860228 | Tt2514 | Aug-2013 | Taiwan: Taitung | (6) |
| Ferret Badger | Glycoprotein | KP860229 | Tt4931 | Nov-2013 | Taiwan: Taitung | (6) |
| Ferret Badger | Glycoprotein | KP860230 | Tt5037 | Dec-2013 | Taiwan: Taitung | (6) |
| Ferret Badger | Glycoprotein | KP860231 | Tt5040 | Dec-2013 | Taiwan: Taitung | (6) |
| Ferret Badger | Glycoprotein | KP860232 | Tt5249 | Dec-2013 | Taiwan: Taitung | (6) |
| Ferret Badger | Glycoprotein | KP860233 | YL2167 | Aug-2013 | Taiwan: Yunlin | (6) |
| Ferret Badger | Glycoprotein | KP860234 | YL5154 | Dec-2013 | Taiwan: Yunlin | (6) |
| Ferret Badger | Glycoprotein | KP881357 | TW-1955 | Aug-2013 | Taiwan: Hualien | (6) |
| Ferret Badger | Glycoprotein | KP881358 | TW-2700 | Aug-2013 | Taiwan: Nantou | (6) |
| Ferret Badger | Glycoprotein | KP881359 | TW-1907 | Aug-2013 | Taiwan: Taichung | (6) |
| Ferret Badger | Glycoprotein | KP881360 | TW-1944 | Aug-2013 | Taiwan: Yunlin | (6) |

**REFERENCES :**

1. Van Zyl N, Markotter W, Nel LH. Evolutionary history of African mongoose rabies. Virus research. 2010;150(1-2):93-102.

2. Nadin-Davis SA, Torres G, Ribas Mde L, Guzman M, De La Paz RC, Morales M, et al. A molecular epidemiological study of rabies in Cuba. Epidemiology and infection. 2006;134(6):1313-24.

3. Zieger U, Marston DA, Sharma R, Chikweto A, Tiwari K, Sayyid M, et al. The phylogeography of rabies in Grenada, West Indies, and implications for control. PLoS neglected tropical diseases. 2014;8(10):e3251.

4. Meng S, Sun Y, Wu X, Tang J, Xu G, Lei Y, et al. Evolutionary dynamics of rabies viruses highlights the importance of China rabies transmission in Asia. Virology. 2011;410(2):403-9.

5. Guo Z, Tao X, Yin C, Han N, Yu J, Li H, et al. National borders effectively halt the spread of rabies: the current rabies epidemic in China is dislocated from cases in neighboring countries. PLoS neglected tropical diseases. 2013;7(1):e2039.

6. Tsai KJ, Hsu WC, Chuang WC, Chang JC, Tu YC, Tsai HJ, et al. Emergence of a sylvatic enzootic formosan ferret badger-associated rabies in Taiwan and the geographical separation of two phylogenetic groups of rabies viruses. Veterinary microbiology. 2016;182:28-34.

7. Meng S, Xu G, Wu X, Lei Y, Yan J, Nadin-Davis SA, et al. Transmission dynamics of rabies in China over the last 40 years: 1969-2009. J Clin Virol. 2010;49(1):47-52.

8. Tao XY, Tang Q, Rayner S, Guo ZY, Li H, Lang SL, et al. Molecular phylodynamic analysis indicates lineage displacement occurred in Chinese rabies epidemics between 1949 to 2010. PLoS neglected tropical diseases. 2013;7(7):e2294.
